# A new species of rorqual whale (Cetacea, Mysticeti, Balaenopteridae) from the Late Miocene of the Southern North Sea Basin and the role of the North Atlantic in the paleobiogeography of *Archaebalaenoptera*

**DOI:** 10.7717/peerj.8315

**Published:** 2020-01-13

**Authors:** Michelangelo Bisconti, Dirk K. Munsterman, René H.B. Fraaije, Mark E.J. Bosselaers, Klaas Post

**Affiliations:** 1 Paleobiology Department, San Diego Natural History Museum, San Diego, California; 2 Dipartimento di Scienze della Terra, Università degli Studi di Torino, Torino, Italia; 3 Toegepast Natuurwetenschappelijk Onderzoek (TNO-Netherlands Organization for Applied Scientific Research), Geological Survey of The Netherlands, Utrecht, The Netherlands; 4 Oertijdmuseum, Boxtel, The Netherlands; 5 Royal Belgian Institute of Natural Sciences, Brussels, Belgium; 6 Natuurhistorisch Museum Rotterdam, Rotterdam, The Netherlands

**Keywords:** Antitropicality, Archaebalaenoptera, Balaenopteridae, Mediterranean salinity crisis, Miocene, North Atlantic Ocean, North Sea Basin, Paleobiogeography, Phylogeny

## Abstract

**Background:**

The rich fossil record of rorqual and humpback whales (Cetacea, Mysticeti, Balaenopteridae) is mainly characterized by monotypic genera since genera including more than one species are extremely rare. The discovery of new species belonging to known genera would be of great importance in order to better understand ancestor-descendant relationships and paleobiogeographic patterns in this diverse group. Recent discoveries in the southern North Sea Basin yielded a number of reasonably well preserved fossil balaenopterids from the Late Miocene; this sample includes a balaenopterid skull from Liessel, The Netherlands, which shares key characters with *Archaebalaenoptera castriarquati* from the Pliocene of Mediterranean. This skull is permanently held by Oertijdmuseum, Boxtel, The Netherlands, with the number MAB002286 and is investigated here.

**Methods:**

A detailed comparative anatomical analysis of the skull MAB002286 is performed in order to understand its relationships. The age of the skull is determined by dinocyst analysis of the associated sediment. A paleobiogeographic analysis is performed to understand paleobiogeographic patterns within the balaenopterid clade the new skull belongs to.

**Results:**

Our work resulted in the description of *Archaebalaenoptera liesselensis* new species. The geological age of the holotype skull is between 8.1 and 7.5 Ma. The phylogenetic relationships of this species reveals that it is monophyletic with *Archaebalaenoptera castriarquati* from the Italian Pliocene. Moreover, in combination with a more basal species of *Archaebalaenoptera* from the late Miocene of Peru, our paleobiogeographic analysis suggests that the North Atlantic ocean played a major role as a center of origin of a number of balaenopterid clades including *Protororqualus*, * Archaebalaenoptera* and more advanced balaenopterid taxa. From a North Atlantic center of origin, two dispersal events are inferred that led to the origins of *Archaebalaenoptera* species in the South Pacific and Mediterranean. The distribution of *Archaebalaenoptera* was antitropical in the late Miocene. The role played by the Mediterranean salinity crisis is also investigated and discussed.

## Introduction

The fossil record of baleen bearing whales (Mammalia, Cetacea, Mysticeti) is mainly characterized by genera known only by one specimen (Bisconti, 2010). Only a few examples exist of chaeomysticete genera with multiple species found at different localities (e.g., *Parietobalaena*, *Diorocetus*, *Balaena*, *Eubalaena*, *Balaenula*). This fact represents a problem for morphologists as it makes it hard or impossible to assess the degree of morphological variation in extinct mysticete species and does not provide safe information about the past distributions of these cetaceans. This situation is even more problematic for the family Balaenopteridae (rorqual and humpback whales) whose extant taxa have a worldwide distribution (Gaskin, 1986). In fact, the balaenopterid fossil record includes a number of monospecific genera known by a limited number of specimens (often only one) distributed in one or a very small number of localities. The discovery of new species of known balaenopterid genera would add important data about the past distribution of these whales and could help reconstructing past speciation patterns eventually linking the evolution of balaenopterid diversity to geodynamic and paleoenvironmental events. The discovery of additional species in known genera may assist in reconstructing the different paths of morphological transformations occurring in separate rorqual lineages. Moreover, discoveries of such kind would also help in assessing paleobiogeographic patterns of single balaenopterid clades. In particular, extant balaenopterids include species with antitropical distribution patterns in which populations living in the northern hemisphere have small-to-no gene flow with co-specific populations living in the southern hemisphere (e.g., Hoeltzel, 1994). Moreover, within Balaenopteridae, a couple of antitropically distributed species is known, i.e., *Balaenoptera acutorostrata* and *Balaenoptera bonaerensis*. Also in Balaenidae, the distribution of the three *Eubalaena* species suggests some sort of antitropicality (Bisconti, Munsterman & Post, 2019). The origin of this kind of distribution represents an interesting zoogeographic problem whose solution could help reconstructing the history of trophic web changes in entire ocean basins (e.g., Briggs, 1987).

Here, we describe a new species of *Archaebalaenoptera* based on a partial skull held by Oertijdmuseum, Boxtel, The Netherlands (hereinafter: MAB), collection number 002286. The skull shows characters of the supraoccipital that support its inclusion within *Archaebalaenoptera* and represents, therefore, the second formally described species of this genus. The only other species of this genus known up to now was *Archaebalaenoptera castriarquati* from the lower Piacenzian (3.55–3.1 Ma) of northern Italy (Bisconti, 2007).

In this paper, we describe the skull MAB 002286 and compare it with a large sample of fossil and living balaenopterid species. The phylogenetic relationships of this specimen were analysed by *Bisconti, Munsterman & Post (2019)* resulting in its inclusion within the genus *Archaebalaenoptera* as sister group of *A. castriarquati*. The analysis also revealed the existence of an additional *Archaebalaenoptera* species from the late Miocene of Peru that will be described elsewhere (Bisconti et al., 2020, unpublished data). Based on this phylogenetic hypothesis, we perform a palaeobiogeographic analysis of Balaenopteridae with the focus on understanding the biogeographic origin and the evolution of the distribution of *Archaebalaenoptera*. Linking phylogenetic relationships and palaeobiogeographic patterns allows us to discuss eventual connections between morphology, distribution and palaeoenvironmental events in this genus.

## Materials & Methods

### Materials

The specimen described in the present work is a single, moderately well preserved skull held by Oertijdmuseum, Boxtel, The Netherlands with the collection number MAB002286. The specimen has been compared with most of the formally described balaenopterid Operational Taxonomic Units (hereinafter: OTU; plural: OTUs); we use the term OTU because some specimens of the balaenopterid fossil record could represent new taxa but did not receive any taxonomic designation (e.g., the Japanese specimens Shimajiri-kujira and the Pliocene balaenopterid from Maesawa-cho; see (Kimura et al., 2015; Oishi, Kawakami & Hasegawa, 1985). The list of the taxa used for comparative purposes is the same provided by Bisconti (2011), Bisconti & Bosselaers (2016) and Bisconti, Munsterman & Post (2019).

### Anatomy

Anatomical terminology follows *Mead & Fordyce (2009)* with additional terms implemented from *Ekdale, Berta & Deméré (2011)* and Kellogg (1965, 1968) where necessary. Specimens used for comparative analysis are presented, together with their repositories and numbers, in the Supplementary Information file.

### Locality and succession

**Figure 1:**
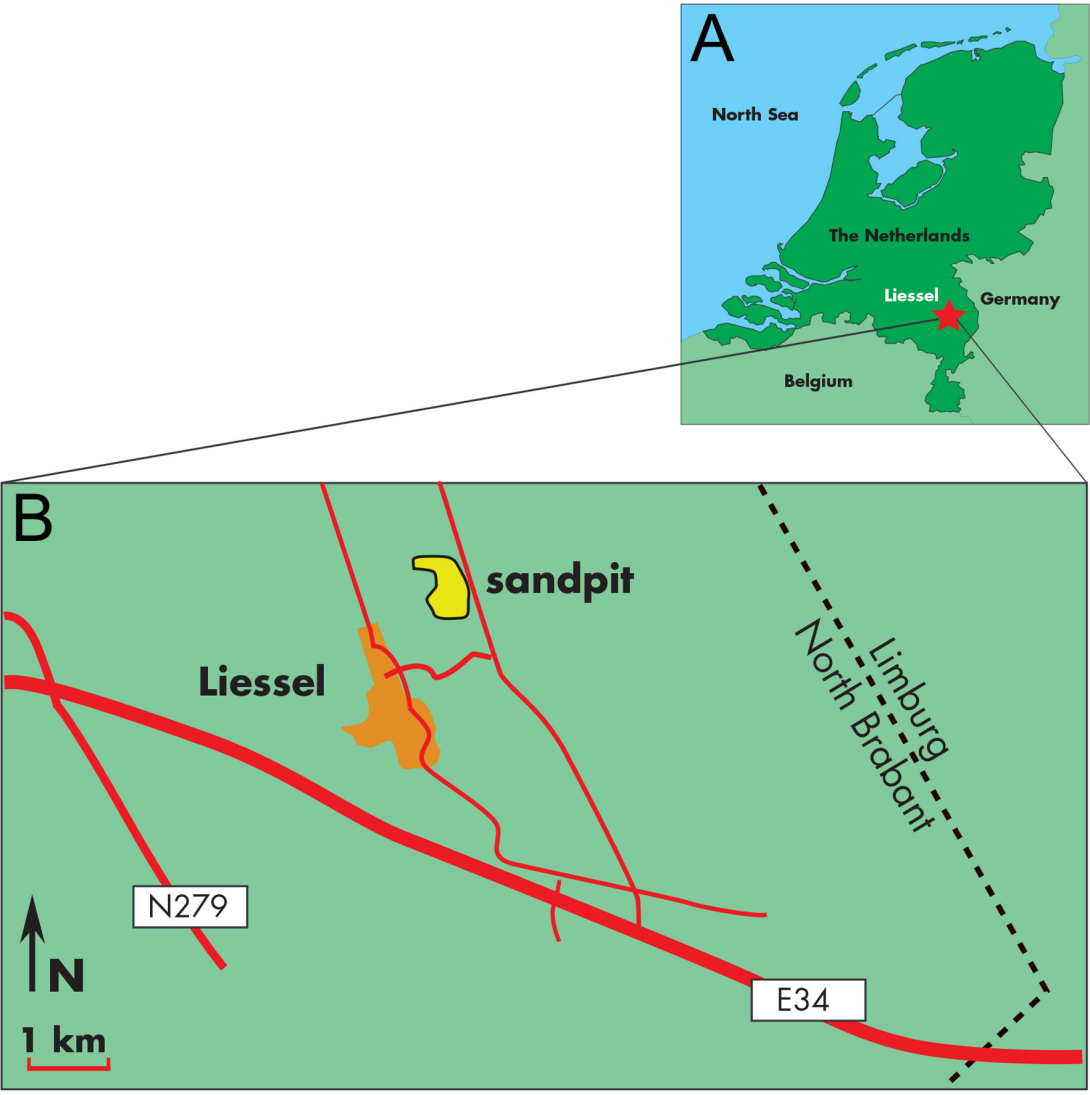
Type locality of *Archaebalaenoptera liesselensis*. Locality of the discovery of the holotype skull of Archaebalaenoptera liesselensis. (A) Map of The Netherlands where a red star shows the locality. (B) close-up view of the locality of the discovery near the town of Liessel.

The skull MAB 002286 is from the sand pit of the brick producing factory Hoogdonk, situated in between the villages Liessel and Deurne, Noord Brabant, the Netherlands (Fig. 1). Borehole B52C1978, interval 0-44.5 m, directly adjacent to the sand pit (at RD-coordinates: (X) 185.627 and (Y) 382.024) was drilled in 2001. The results of the lithostratigraphic and palynological interpretations are shown in Table S1 (Munsterman, 2007).

### Palynological preparation and analysis

Fine-grained glauconitic sands (the matrix) cemented on the inner part of the present balaenopterid skull were prepared at Palynological Laboratory Services (PLS, UK). The standard sample processing procedures are used, which involves HCl and HF treatment, heavy liquid separation, and sieving over a 15 µm mesh sieve. The organic residue was mounted with glycerin-gelatin on microscopic slides. The palynological analysis was carried out at the Geological Survey of the Netherlands (TNO) according to standard procedures. This involves a microscopic slide count using 500×magnification, until approximately a total of 200 sporomorphs (pollen and spores) and marine dinoflagellate cysts is reached. The main miscellaneous categories (e.g., marine acritarchs, test linings of foraminifers and brackish water algae *Botryococcus*) are calculated separately. The remainder of the slide is thereafter scanned for any (rarer) dinocyst species. Diagnostic species are discussed in a following chapter, a complete distribution chart including all species recorded is given as Table S2. The age interpretation is based on the Last Occurrence Datum (LOD) and First Occurrence Datum (FOD) of dinoflagellate cysts. Dinoflagellate cyst taxonomy follows Williams, Fensome & MacRae (2017). Palynological interpretation is based on key-references concerning the palynostratigraphy of the Neogene from the North Sea region such as: *Dybkjær & Piasecki (2010), Köthe (2012), Kuhlmann et al. (2006), Louwye, Head & De Schepper (2004), Louwye & De Schepper (2010), Munsterman & Brinkhuis (2004)* and *Powell (1992)*. The Geological Time Scale 2016 is used (Ogg, Ogg & Gradstein, 2016). For the dinozones is referred to Munsterman & Brinkhuis (2004) recalibrated to Ogg, Ogg & Gradstein (2016).

### Nomenclatural act

The electronic version of this article in Portable Document Format (PDF) will represent a published work according to the International Commission on Zoological Nomenclature (ICZN), and hence the new names contained in the electronic version are effectively published under that Code from the electronic edition alone. This published work and the nomenclatural acts it contains have been registered in ZooBank, the online registration system for the ICZN. The ZooBank LSIDs (Life Science Identifiers) can be resolved and the associated information viewed through any standard web browser by appending the LSID to the prefix http://zoobank.org/. Publication LSID is urn:lsid:zoobank.org:pub:41E2B619-3E94-4299-956E-CAF7237A147F. The online version of this work is archived and available from the following digital repositories: PeerJ, PubMed Central and CLOCKSS.

### Source of phylogenetic tree

We used the phylogenetic results of Bisconti, Munsterman & Post (2019) that were based on 350 morphological characters scored for 85 taxa. Character descriptions were provided by *Bisconti, Munsterman & Post (2019)* but we commented and clarified 54 characters included in the original dataset for what concerns quantitative interpretations of the data (see Supplementary Information). We preferred using the analysis of *Bisconti, Munsterman & Post (2019)* as it includes more balaenopterid taxa than all the other published analyses. Moreover, it includes a high number of morphological characters and it is based on observation of individual variation in several mysticete groups as detailed by *Bisconti, Munsterman & Post (2019)*. For instance, one of the well-resolved balaenopterid cladogram was provided by *Marx & Kohno (2016),* but we decided to use the *Bisconti, Munsterman & Post (2019)* dataset because in the former the balaenopterid taxa included are 16, and in the latter the balaenopterid taxa are 33, thus doubling the taxonomic sampling of Balaenopteridae.

### Palaeobiogeographic analysis

Geographic occurrences of the OTUs were mapped onto the tree and reconstructions of ancestral occurrences were inferred through Fitch’s (1971) parsimony, maximum parsimony (MP) and maximum likelihood (ML). We used MESQUITE 3.6 Maddison & Maddison, 2009) for MP and ML mappings. Eight geographic characters were added to the matrix that were not used for phylogenetic inference (Table S3). These characters included areas where fossil or living baleen whale species occurred (i.e., Mediterranean, North Atlantic, North Pacific, South Atlantic, South Pacific, Indian ocean, Arctic Polar Circle and Paratethys) (see Supplementary Information file); taxa were scored for presence/absence based on occurrence data presented in Table S4. The matrix with mapped characters is provided in Table S5. We considered that taxa described from the Miocene and Pliocene outcrops of the southern North Sea basin to be widespread in the North Atlantic ocean as it is hard-to-impossible to demonstrate that living or fossil balaenopterid species may have had a range reduced only to the southern North Sea basin because of their ability to swim across ocean basins of today.

In order to reconstruct the paleobiogeographic history of *Archaebalaenoptera*, reconstructions of ancestral occurrences at nodes was made through Fitch’s (1971) parsimony, and MP and ML approaches with Mk1 distribution model and parameters estimated by MESQUITE 3.6 Maddison & Maddison, 2009) with default settings. This reconstruction process allows us to infer the most likely geographic distribution at ancestral nodes; this datum is necessary to infer eventual dispersal or vicariance patterns associated to the terminal clades of the phylogenetic tree.

## Systematic Palaeontology

**Table:** 

Mammalia *Linnaeus, 1758*
Artiodactyla Owen, 1848
Cetacea Brisson, 1762
Pelagiceti Uhen, 2008
Neoceti Fordyce & De Muizon, 2001
Mysticeti Flower, 1864
Chaeomysticeti Mitchell, 1989
Thalassotherii Bisconti, Lambert & Bosselaers, 2013
Balaenopteridae Gray, 1864
*Archaebalaenoptera*Bisconti, 2007

**Emended diagnosis of genus.** Supraoccipital with rounded anterior border and strong transverse constriction; posterior apex of nuchal crest triangular in dorsal view and directed posteriorly; supraoccipital horizontally bent approximately at mid-length; zygomatic process of squamosal projecting anterolaterally and with sharply defined supramastoid crest; interorbital region of the frontal with transversely rounded sides; posterolateral corner of exoccipital protruding posteriorly.

**Discussion**. *Archaebalaenoptera* differs from other balaenopterids in having a unique mix of morphological characters. In particular, while the transverse constriction of the supraoccipital is observed also in *‘Balaenoptera’ cortesii* var. *portisi*, in *Archaebalaenoptera* the constriction is associated with a rounded anterior border of the supraoccipital; in *‘B.’ cortesii* var. *portisi* the anterior border of the supraoccipital is narrow-to-pointed. The extended posteriorly protruding posterolateral corner of the exoccipital is also observed in *Fragilicetus velponi* but in that taxon, the supraoccipital is largely different being more squared at its anterior border and the being transverse constriction very reduced. In cross section, the round sides of the interorbital region of the frontal are typical of this genus and represent a marked difference with other balaenopterid genera where the sides of the interorbital region of the frontal are squared or concave.

**Type species.** *Archaebalaenoptera castriarquati Bisconti, 2007*

*Archaebalaenoptera liesselensis* sp. nov.

**Diagnosis of species.** Presently, autapomorphic characters are not observed in *Archaebalaenoptera liesselensis*; this could be due to the fact that some of the anatomical districts preserved in the holotype cannot be investigated in the other *Archaebalaenoptera* species (e.g., periotic, endocranial cavity etc.). *Archaebalaenoptera liesselensis* differs from *Archaebalaenoptera castriarquati* in lacking the dorsally rounded dome at the anterior portion of the supraoccipital (Table S8 and Fig. S5), it lacks the strong tubercles for the attachment of neck muscles on the lateral borders of the supraoccipital, and lacks narial processes of the interorbital region of the frontal. *Archaebalaenoptera liesselensis* differs from the undescribed new *Archaebalaenoptera* species (MHNL 1610; Bisconti et al., 2020, unpublished data; see Bisconti & Bosselaers, 2016 and Bisconti, Munsterman & Post, 2019 for an overview) in that it is larger (supraoccipital length of *A. liesselensis* is 310 mm and that of MHNL 1610 is 287 mm) and has a differently shaped interorbital region of the frontal (e.g., it does not have narial process and comparatively wider and shorter interorbital region of the frontal with respect to *A. castriarquati*).

**Holotype.** Skull MAB002286 held by Oertijdmuseum, Bosscheweg 80, 5283 WB Boxtel, The Netherlands.

**Etymology.** The species name, *liesselensis*, is from Liessel, the nearest village to the discovery site.

**Horizon and locality.** The skull MAB 002286 was dredged from a sand pit near the village of Liessel in Noord Brabant, The Netherlands (geographic co-ordinates of the site: 51°25′44″N; 05°49′47″E). The pit contains from 0 to 8 m Late Pleistocene sediments, from 8 to 13 m Early Pleistocene sediments, and from 13 to 45 m Late Miocene sediments (‘groenzanden - greensands’ with some wood and clay lenses) (Peters, 2009) . In The Netherlands, the latter sediments originate from the widespread marine Breda Formation, which, at this site, is deposited in a delta-front setting (Peters, 2009) . The holotype was (and to some extend still is) partly embedded in a dense glauconitic and sandy ‘greensand’ matrix from the Late Miocene (Tortonian; between 8.1 and 7.5 Ma; see below for full explanation) suggesting that it is from the Breda Formation.

## Results

### Palynomorph analysis and age assessment

#### Palynofacies

The preservation of the palynomorph (dinoflagellate cyst and sporomorph) assemblage recovered from the greensands associated with the current balaenopterid fossil is moderate to good. The recovery of the palynomorphs however does not fully meet our standard level of counting at least 200 specimens (here 57%). The dinoflagellate cyst ratio is 42% (of the total sum of dinoflagellate cysts and sporomorphs; standard reference for percentages in this paper). Bisaccate pollen dominate the microflora (51%). Bisaccate pollen are formed by gymnosperms (Gymnospermae). These are usually considered to flourish in a relatively dry environment as higher pine forests. However bisaccate pollen grains have a high buoyancy in both air and water, hence can easily be transported to distal marine settings, even open oceans, excluding any spores (Abbink, 1998). The category spores are here present in relatively low percentages (7%). The highest numbers of marine dinocysts are reached by the taxa *Spiniferites* spp. and *Operculodinium centrocarpum*. The genus *Spiniferites* has a preferential orientation for open marine neritic conditions (e.g., Brinkhuis, 1994). *Operculodinium centrocarpum* is a cosmopolitan, opportunistic taxon, hence influenced by multiple ecological factors Hennissen et al., 2017). The coastal marine taxon *Lingulodinium machaerophorum* is also well-represented. Remarkable are the relatively high values of heterotrophic genera like *Barssidinium*, *Lejeunecysta* and *Selenopemphix*. Heterotrophic dinoflagellate cysts are associated with nutrient-rich water. All indicators combined indicate nutrient-rich neritic conditions. No reworking is noted.

#### Age assessment

The chronostratigraphic range of the dinoflagellate cyst *Impagidinium densiverrucosum* indicates an age in the Late Miocene (Von Daniels et al., 1990; Zevenboom, 1995). The presence of *Operculodinium piasecki* fits with this dating. The most important chronostratigraphic marker in the assemblage is *Hystrichosphaeropsis obscura.* The Last Occurrence (LO) of this taxon defines the top of the *Hystrichosphaeropsis obscura* biozone of Denmark (*Dybkjaer & Piasecki, 2010*) and the DN9 Zone of the Eastern USA and Germany (De Verteuil & Norris, 1996; ; Köthe, 2012). In the Netherlands the event is associated with the top of M14 Zone (Munsterman & Brinkhuis, 2004), in the late Tortonian (excluding the latest part). Marker species for an early-mid Tortonian stage, or older, are all missing. Hence the most probable dating is in the Late Miocene, Zone M14, ca. 8.1–7.5 Ma Munsterman & Brinkhuis, 2004, recalibrated to (Ogg, Ogg & Gradstein, 2016). This fits with earlier interpreted palynological results of the succession belonging to the Breda Formation, from which the specimen comes, in borehole B52C1978 (Hoogdonk).

### Skull description

#### Overview and preservation

**Figure 2:**
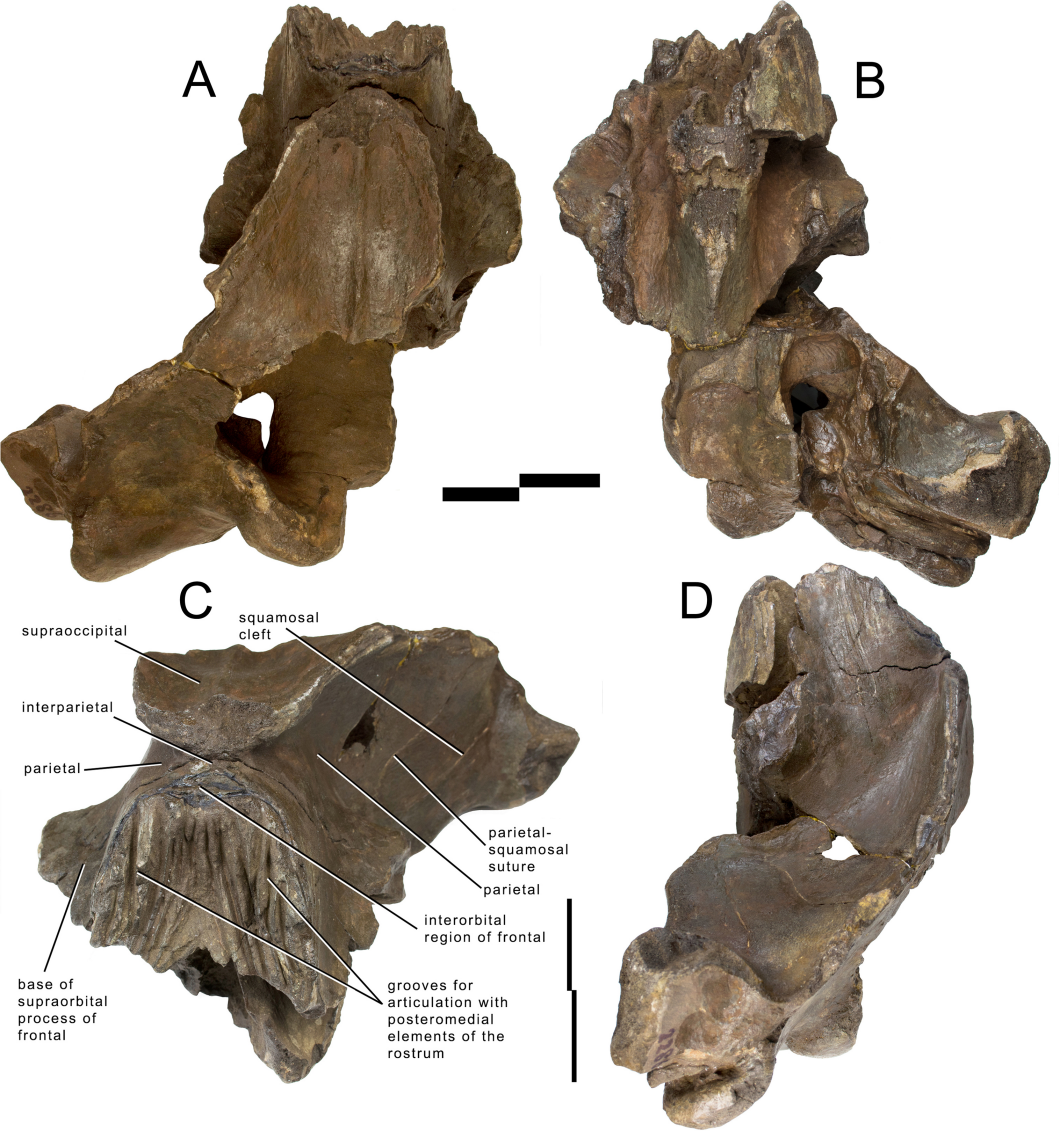
Holotype skull of *Archaebalaenoptera liesselensis*. Photographic representations of the holotype skull of *Archaebalaenoptera liesselensis* (OMB D2286). (A) Dorsal view. (B) Ventral view. (C) Anterolateral view. (D) Lateral view (anterior part is up). Scale bar equals 10 cm. Photography: Jaap Van Leeuwen.

**Figure 3:**
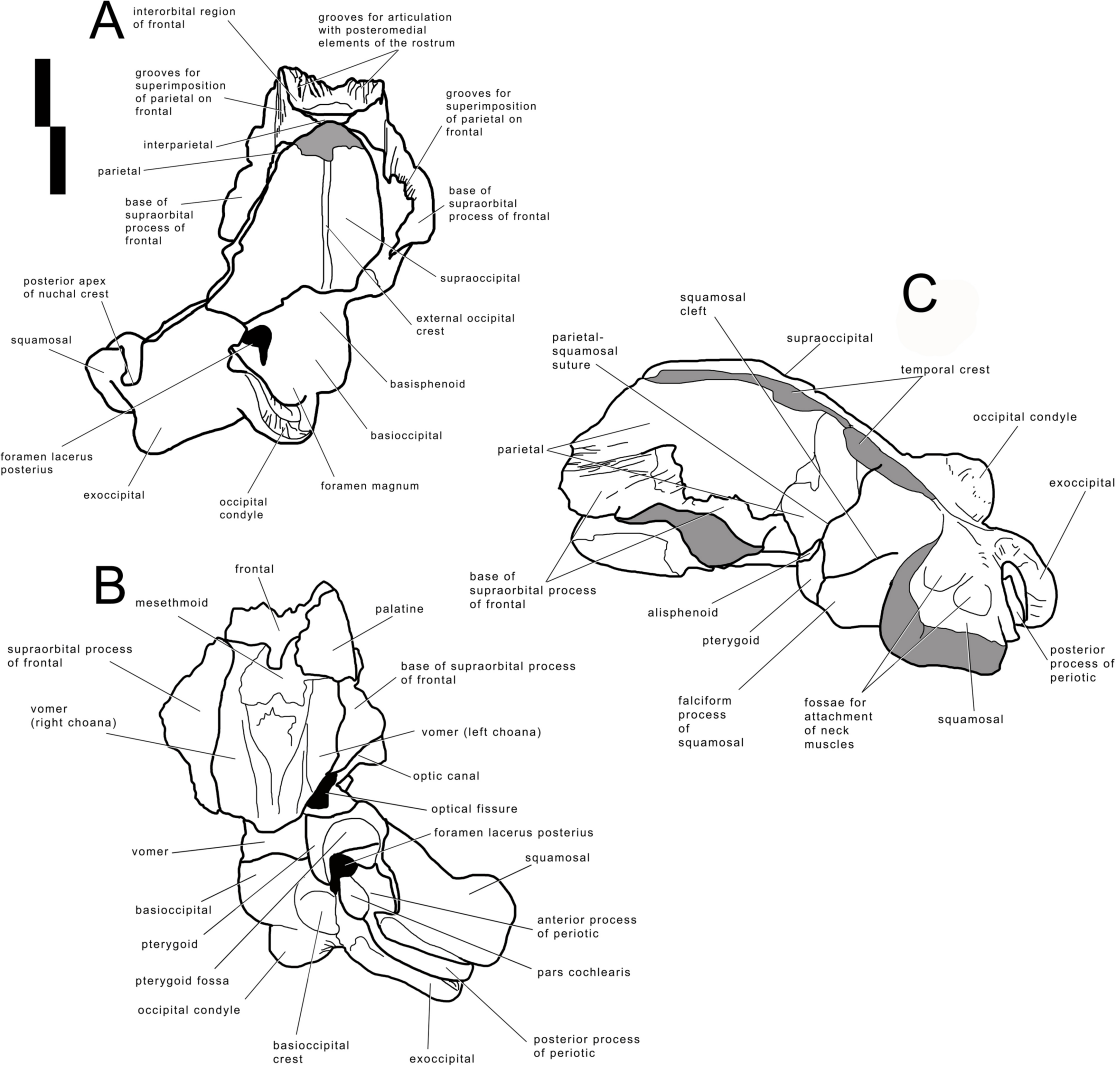
Holotype skull of *Archaebalaenoptera liesselensis*. Interpretation of morphological characters of the holotype skull of *Archaebalaenoptera liesselensis*. (A) Dorsal view. (B) Ventral view. (C) Lateral view. Damaged areas are in grey. Scale bar equals 10 cm.

The skull of *Archaebalaenoptera liesselensis* lacks the rostrum, both supraorbital processes of the frontal, most of the squamosals and the tympanic bullae. One periotic is still in articulation. Most of the right side of the skull is broken and the endocranial cavity is exposed allowing the observation of the medial surface of the periotic. The remainder of the skull is well preserved. Standard views of the skull are presented in Figs. 2 and 3. Measurements are provided in Tables S6 and S7 and discussed in the text below.

The lack of the lateral border of the supraoccipital is particularly problematic in the process of reconstructing the original skull shape. As shown in Fig. 2D, in lateral view, the truncated border of the supraoccipital and of the parietal are evident. Observations of balaenopterid skulls in anterior view shows that the lateral borders of the supraoccipital do not reach the external rim of the temporal crest that is formed by the parietal ventrally and laterally (see, for instance, Marx & Kohno, 2016; Bisconti, Munsterman & Post, 2019). The lateral border of the supraoccipital is located slightly medially to the external border of the skull at the level of the temporal crest. This means that a smaller portion of the supraoccipital was severed by post-mortem processes in the holotype skull of *Archaebalaenoptera liesselensis*. This consideration is further confirmed by the observation of the lateral view of the severed dorsolateral border of this skull where the larger severed component belongs to the parietal. Thus, we expect that the lateral extension of the supraoccipital was a little more developed than in the holotype skull but we do not think that such an extension was massively wider. We expect that only a few mm, maybe up to 10 mm, were lacking from the lateral border of the supraoccipital and this allows us to assess the general shape of the supraoccipital in dorsal view. Our description of the skull is also based upon this assumption.

#### Frontal

Interorbital region of the frontal and bases of supraorbital processes of the frontal are preserved (Figs. 2 and 3). The interorbital region of the frontal shows articulation grooves for the attachment of the posterior end of the posteromedial elements of the rostrum. There are 6 grooves on the left side and 5 on the right side. It is difficult to determine which groove belongs to the articulation with the maxilla, which with the premaxilla (if any) and which with the nasal. It is reasonable to suppose that most of the grooves belonged to the articulation with the maxilla suggesting that the posterior border of the ascending process of the maxilla was rounded and not squared as in the living species; this character is observed in all the *Archaebalaenoptera* species. Laterally to the grooves, the interorbital region of the frontal is a subtle stripe of bone that surrounds the grooves (and the posterior portion of the ascending process of the maxilla). The transverse diameter of the interorbital region of the frontal increases anteriorly. Along the longitudinal axis of the skull, the interorbital region of the frontal is located in between the grooves for the articulation with posteromedial elements of the rostrum but at a much lesser extension than in *Archaebalaenoptera castriarquati*. If the nasals would have been present between the ascending processes of maxillae and premaxillae, then, judging from the directions of the grooves for the articulation with the posteromedial elements of the rostrum, they should have had largely diverging lateral borders.

**Figure 4:**
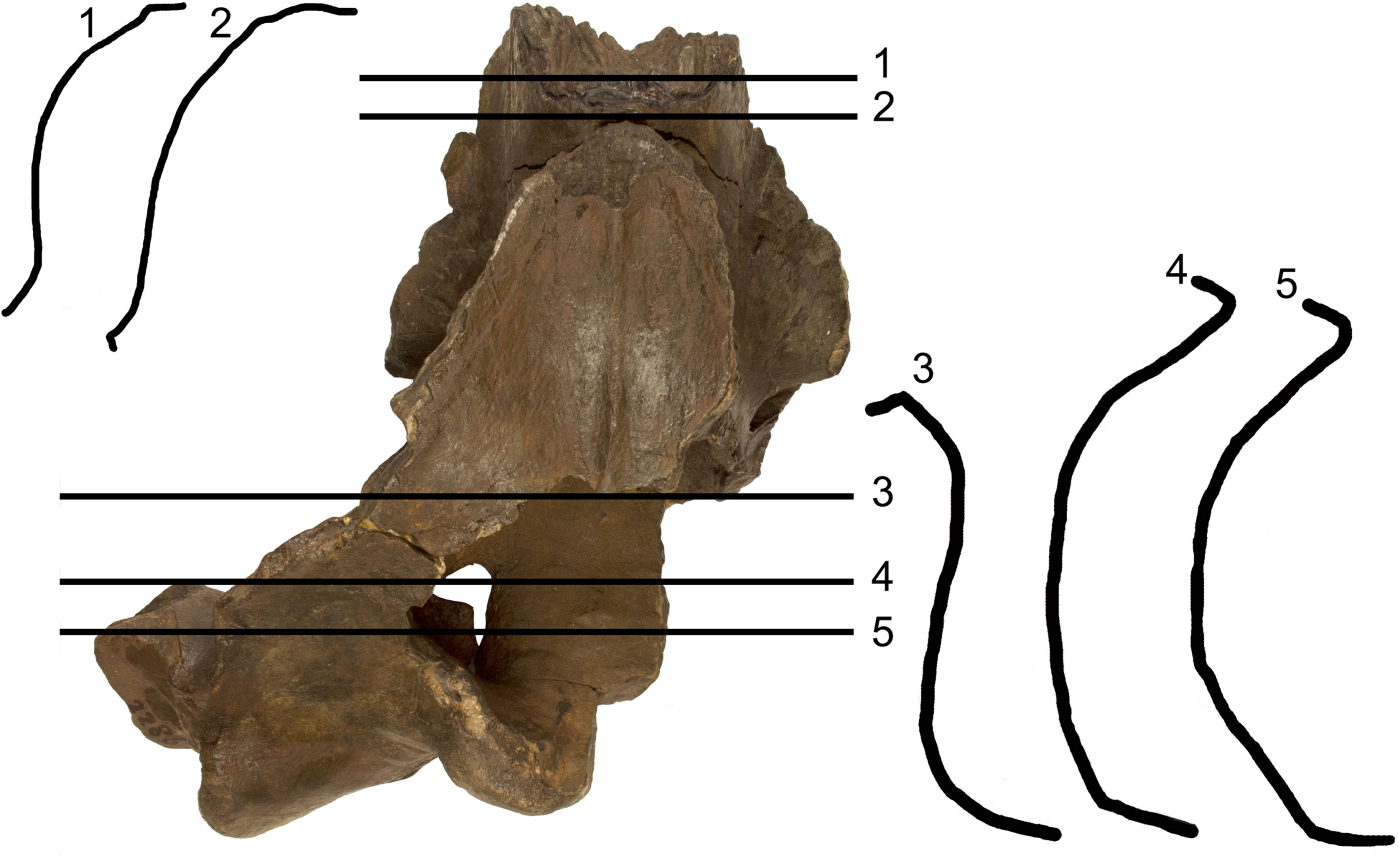
Transverse sections of the holotype skull of *Archaebalaenoptera liesselensis*. Transverse sections of the holotype skull of *Archaebalaenoptera liesselensis*. Sections 1 and 2 are done closely to the vertex and show the round transverse profile of the depression of the supraorbital process of the frontal. Sections 3, 4 and 5 are done across the temporal fossa and show the degree of overhanging of the temporal crest on the parietal. Not to scale. Photography: Jaap Van Leeuwen.

The supraorbital process of the frontal is abruptly depressed from the interorbital region of the frontal (Fig. 4). Judging from the bases of the supraorbital processes, these portions of the frontal were flat. The optic canal is located immediately anteriorly to the posterior border of the supraorbital process of the frontal (Fig. S1). There is no postorbital ridge. Grooves located on the dorsomedial surface of the supraorbital process of the frontal demonstrate that the parietal is spreading onto and covering the frontal along the depression lateral to the interorbital region of the frontal. The depressed portion of the interorbital region of the frontal has a round profile in cross section (Fig. 4). This character is typical and diagnostic of *Archaebalaenoptera* species. The supraorbital process of the frontal was dorsoventrally high (dorsoventral height along the posterior border is 56 mm on the right side and 61 on the left side). A small foramen is observed on the right supraorbital process of the frontal; this foramen is prolonged into an anterolaterally directed groove.

#### Parietal

The anterior portion of the parietal projects anteriorly and interdigitates with the interorbital region of the frontal and the ascending process of the maxilla (Figs. 2 and 3). In its anterior-most portion, the parietal is worn but the anterior point reached by the parietal is documented by the presence of grooves on the frontal. These grooves correspond to the surface of the overlapping of the parietal on the frontal. Anteriorly to the supraoccipital, the parietal is laterally convex along the dorsoventral axis of the skull forming an almost perfect half circle, as it is typical of the genus *Archaebalaenoptera* (Fig. 4). The parietal is externally concave at the base of the supraorbital process of the frontal. The anterior portion of the parietal projects medially and dorsally to surround the posterolateral corner of the interorbital region of the frontal; right and left parietals do not meet along the midline as they are separated by the interposition of an interparietal (Fig. 3). At the level of the maximum projection of the temporal crest, the parietal is strongly concave laterally along the dorsoventral axis of the skull (Fig. 3). The dorsal edge of the parietal is not preserved but appears to have protruded laterally following the lateral borders of the supraoccipital forming the temporal crest. Judging from the current state of preservation, the temporal crest was sufficiently developed to prevent the observation of the medial wall of the temporal fossa in dorsal view. The parietal-squamosal suture starts from the contact point of parietal and pterygoid immediately posterior to the alisphenoid exposure and proceeds dorsally and posteriorly along a sinuous path. A wide and triangular window is open on the medial wall of the temporal fossa 35 mm posteriorly to the supraorbital process of the frontal; this window is caused by damaging post-mortem processes and measures 35 mm in maximum diameter and 34 mm in minimum diameter.

#### Alisphenoid and temporal fossa

The alisphenoid exposure in the temporal fossa is limited to a small surface included between parietal, pterygoid and squamosal (Fig. 3). The dorsoventral diameter of the alisphenoid exposure is 20 mm and the anteroposterior diameter is 34.8 mm so that the bone is rectangular in shape. In the current preservation state, the superior portion of the falciform process of the squamosal terminates more posteriorly than the inferior portion resulting in a wider exposure of the dorsal portion of the pterygoid in the temporal fossa (the dorsoventral diameter of the pterygoid exposure is 62 mm and the anteroposterior diameter is 33 mm). Moreover, the ventral border of the parietal is partially eroded so that a wider portion of the alisphenoid is exposed. The correct reconstruction of this part of the temporal fossa is provided in Fig. 3 where it is possible to observe that the alisphenoid-parietal suture is dorsally concave; the alisphenoid-squamosal suture is limited to a point and the alisphenoid-pterygoid suture is convex. Based on this reconstruction, the alisphenoid exposure in the temporal fossa is limited to a short stripe of bone.

#### Squamosal

The parietal-squamosal suture begins from the posterodorsal corner of the alisphenoid and sinuously projects posterodorsally. Approaching the supraoccipital, the area including the parietal-squamosal suture becomes laterally convex along the anteroposterior axis. This area turns to be concave in close proximity to the posterior apex of the nuchal crest (sensu *Mead & Fordyce, 2010*; this portion is also known as lambdoid crest, see *Mead & Fordyce, 2010*, p. 34). The posterior development of the nuchal crest is rounded in cross-section and its posterior apex reaches a point located more anteriorly than the occipital condyles. The area included between the parietal-squamosal suture and the squamosal cleft is mostly flat along the dorsoventral and the anteroposterior axes; this area becomes convex in its more posterior portion. The squamosal cleft is straight and is 127 mm in length. It ends 62 mm anteroventrally from the posterior apex of the nuchal crest. The lateral surface of the squamosal shows two fossae for insertion of the neck muscles (very probably, the sternocephalicus muscle). These fossae are clearly separated by an anteroposterior crest.

In ventral view, the squamosal is a robust block characterized by a main axis forming nearly a right angle with the anteroposterior axis of the skull. External acoustic meatus and zygomatic process of the squamosal are not preserved entirely. Only the medial portion of the external acoustic meatus is present, it is 31 mm in anteroposterior diameter and shows a strong ventral concavity along its transverse development. Based on this observation, it is expected that the external acoustic meatus was long (probably 120 + mm) and anteroposteriorly narrow.

#### Pterygoid

In lateral view, only a small stripe of pterygoid is visible that corresponds to the dorsal lamina (Fig. 3). This portion is located anteriorly to the squamosal and ventrally to the alisphenoid. The pterygoid fossa is well preserved on the left side of the skull (Fig. 2). The palatal surface of the pterygoid fossa is anteriorly wide and its anterior and posterior borders are posteriorly concave. The posterior border of the palatal surface is obliquely oriented in the sense that it proceeds from an anterolateral point to a posteromedial point. The posterior border of the palatal surface forms the anterior border of the foramen lacerus posterius.

#### Supraoccipital

In dorsal view, the supraoccipital is roughly triangular with a remarkable transverse constriction located approximately at the middle of its anteroposterior length (Figs. 2 and 3). The anterior portion is partially broken but the correct outline may be easily reconstructed by mirroring the contralateral part. As the lateral edges of the supraoccipital are broken, based on the thickness of the preserved borders, we estimate that the supraoccipital border protruded up to 10 mm more laterally in the anterior-most quarter of the supraoccipital, and up to 50 mm in the central portion. Based on these estimates, we reconstructed the extent of the transverse constriction of the supraoccipital and the shape of the temporal crest as shown in Fig. S5. The anterior border is rounded and transversely narrow. The lateral borders project laterally and posteriorly forming protruding temporal crests that prevent the observation of the medial wall of the temporal fossa in dorsal view. The maximum transverse constriction of the supraoccipital is located *c.* 200 mm from the anterior end and *c.* 220 mm from the posterior apex of the nuchal crest being approximately located at the middle of the anteroposterior length of the supraoccipital.

The dorsal surface of the supraoccipital is anteriorly worn for about 58 mm; more posteriorly, it shows the presence of an external occipital crest laterally bordered by a bilateral fossa that is anteroposteriorly and transversely concave. About 190 mm from the posterior apex of the nuchal crest, the supraoccipital inclination changes: in fact, the anterior portion of the supraoccipital is almost horizontal while the posterior portion is posteroventrally bent.

No tubercles for the attachment of neck muscles are observed.

#### Exoccipital

The wide exoccipital projects posteriorly and laterally (Figs. 2 and 3). The posterolateral corner of the exoccipital reaches a point located slightly posteriorly to the posterior articular surface of the occipital condyle. The surface of the exoccipital is convex; the ventral border is straight in posterior view. The occipital condyle is wide and robust; its articular surface is dorsoventrally convex and transversely flat. The articular surface is mainly developed ventrally to the ventral border of the foramen magnum. Only a minimal portion of the foramen magnum is still preserved being bordered by the preserved part of the left occipital condyle, and this prevents us to describe this structure in detail.

#### Basioccipital

The ventral surface of the basioccipital is flat along the longitudinal axis of the skull (Figs. 2 and 3); the surface becomes concave where the basioccipital crest are protruded. The basioccipital crest is tubercle-like and shows a ventrally-faced articular surface for the hyoid. The basioccipital crest forms the medial border of the foramen lacerus posterius; this border has a sinuous outline in ventral view. The occipital condyles and the basioccipital crests are separated by the interposition of a wide transverse groove (anteroposterior diameter of 40 mm). The jugular notch is wide, very concave and lacks any foramen. There is no condyloid foramen.

The endocranial cavity is exposed due to a large breakage of the right posterolateral side of the skull (Fig. S2). The endocranial face of the basioccipital and of the basisphenoid is mostly flat; the sella turcica is a slight and elliptical depression located slightly anteriorly to the anterior edge of the foramen lacerus posterius. The lateral wall of the endocranial cavity is almost uniformly concave with a notably transverse constriction at the level of the foramen magnum.

#### Palatine

A fragment of the left palatine is preserved in the anterior portion of the skull, ventrally (Fig. 2). It shows a convex external surface and is 16 mm in medial thickness. Its thickness decreases at mid-length where it is only eight mm. More posteriorly, the thickness increases again as it becomes 13 mm. The internal surface is highly concave and forms the internal border of the left choana. The choana is relatively wide with its maximum dorsoventral diameter of 62 mm, maximum transverse diameter of 44 mm and maximum length of 170 mm. Medially, the palatine stays on the vomer. Only the dorsal portion of the right choana is preserved. The choana has a dorsoventral diameter of 58 mm and a length of 200 mm.

#### Vomer

In anterior view, the vomer shows a rounded ventral border that corresponds to the posterior-most portion of the bone. The posterior keel is largely worn but is evident along the more posterior portion (around 80 mm in length). Laterally to the keel, the lateral surface of the vomer is slightly concave along the dorsoventral axis. The posterior portion of the vomer is reduced to a very subtle bony lamina that is superimposed to the basioccipital-basisphenoid suture. The posterior border of the vomer is posteriorly concave.

#### Periotic

**Figure 5:**
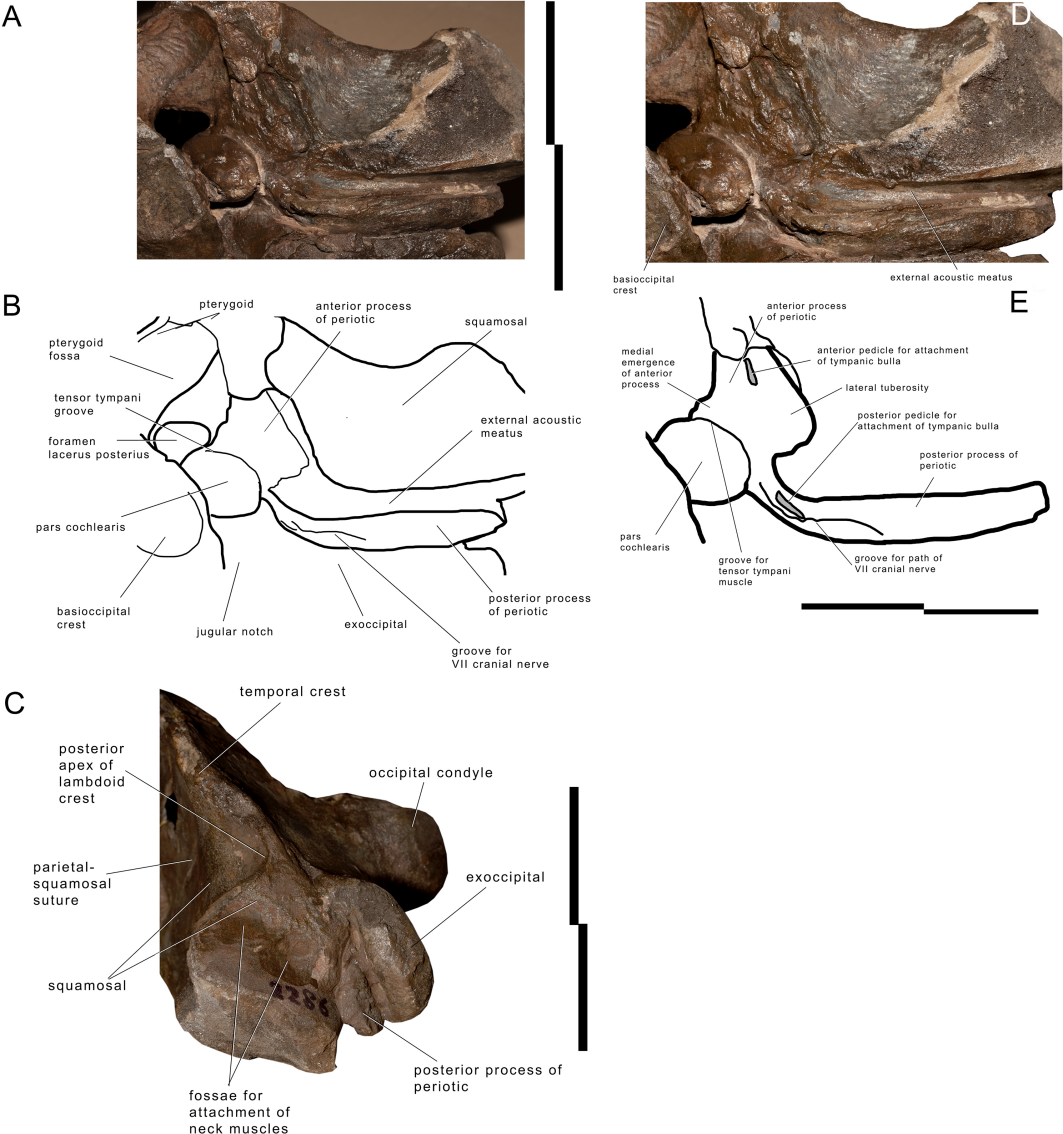
Left periotic and associated structures in * Archaebalaenoptera liesselensis*. Left periotic and associated anatomical structures. (A) Left posterolateral corner of the skull showing the periotic still in articulation. (B) Left posterolateral corner of the skull showing additional periotic characters. (C) Left posterolateral corner of the skull in lateral view showing the exposure of the posterior process of the periotic. Scale bar equals 10 cm. Photography: Michelangelo Bisconti.

The left periotic is still firmly articulated with the skull and parts of its borders are still covered by hard matrix (Fig. 5). Measurements of the periotic are provided in Table S7. Preparation was complete in the ventral surface but it is hard to get access to the medial surface where preparation is complicated and not complete. The posterior process is long and forms a right angle with the anterior process. It is anteroposteriorly compressed but robust. The distal portion is exposed laterally posterior to the postglenoid process of the squamosal and can be observed in the skull in lateral view (Fig. 5). The anterior border is in contact with the external acoustic meatus whose posterior border is formed by the posterior meatal ridge formed by the squamosal. This lamina is very subtle (anteroposterior thickness about 1.2 mm), ventrally concave and transversely elongated. The anteroposterior diameter of the external acoustic meatus decreases from the medial to the lateral portions (medial length, 24 mm; length at mid-length, 11 mm; lateral length, 11 mm).

The (broken) posterior pedicle for the tympanic bulla is located along the anteroventral border of the posterior process at about 122 mm from the posterolateral border and is anteroposteriorly compressed (Fig. 5). Its length is 16 mm. The same border forms the anterior edge of the facial canal that runs along the medial portion of the posterior process; the edge is not visible anymore starting from 81 mm from the lateral border of the posterior process.

Medially, the posterior process forms a curve with anterior concavity and is connected to the pars cochlearis. The length of the posterior process as a whole is *c.* 41 mm and the connecting neck to the pars cochlaris is particularly long. The groove for the facial nerve running along the posterior process is anteroposteriorly concave; it is 62 mm in length and 20 mm in width.

The anterior process is triangular in ventral view and its anterior end is located under the squamosal, just posteriorly to the posterior border of the pterygoid fossa. The posteromedial border of the anterior process shows a medial eminence with a convex medial border (Fig. 5). This eminence is called here Medial Eminence of the Anterior process (hereinafter: MEA). The peculiar shape of the MEA border gives the medial border of the anterior process a sinuous shape. A triangular and protruding lateral tuberosity is evident along the lateral border of the anterior process just laterally to the pars cochlearis. The (broken) anterior pedicle for the tympanic bulla is straight and narrow, and is located along the sharply-edged lateral border of the periotic. The anterior pedicle for the tympanic bulla is 15 mm in length and three mm in width. The ventral surface of the anterior process is flat-to-slightly concave near the lateral tuberosity; this surface is then anteroposteriorly convex approaching the medial border of the anterior process. A medial groove is interposed between the MEA and the anterior-most portion of the anterior process. This groove runs from a posteriormedial point to an anterolateral point and merges in the medial border of the anterior process.

The pars cochlearis is transversely protruded and is separated from the anterior process by an evident groove for the tensor tympani. The pars cochlearis is also ventrally protruding (Fig. S3). The distance between the superior border of the pars cochlearis and the ventral surface of the anterior process is reduced and, therefore, structures such as the distal opening of the facial canal and the oval window are dorsoventrally much smaller than expected based on comparative analysis of other living and fossil balaenopterid whales. The caudal tympanic process is not preserved; however, a foramen is present to allow the passage of the facial nerve that merges into the groove for the facial nerve that is present along the posterior process of the periotic. The oval window is elliptical in shape (Table S7). The fossa for the stapedial muscle is highly compressed dorsoventrally and is not separated from the oval window by a crest. Only the posterior border of the round window is evident, the remainder and the entire posterior face of the pars cochlearis being invaded and covered by matrix.

The endocranial surface of the pars cochlearis cannot be observed in detail. In Fig. S4, it is possible to observe the endocranial opening of the facial canal located in the inferior-most part of the pars cochlearis itself. The suprameatal surface is highly concave but its dorsal border is still obliterated by matrix and cannot be distinguished from the endocranial face of the skull.

**Endocranial cavity.** The endocranial cavity is laterally concave and ventrally flat (Fig. S2). The lateral walls are formed by parietal, squamosal and alisphenoid. The alisphenoid-parietal suture is highly interdigitated from the inside of the skull and terminates dorsally in a wide, triangular hole, and ventrally in the foramen lacerus posterius. The internal side of the alisphenoid is elongated and projects dorsally and anteriorly. It is not clear if it contributes to the posterodorsal portion of the optic channel. The basioccipital-basisphenoid is fused and its original position cannot be determined. The basisphenoid-presphenoid suture is still unfused. The internal surface of the basioccipital is transversely convex and anteroposteriorly flat. The sella turcica is a shallow and elliptical concavity in the endocranial surface of the basisphenoid. Posteriorly and laterally, the exoccipital is highly concave laterally to the foramen magnum.

## Comparisons

### Comparisons to other *Archaebalaenoptera* species

The new *Archaebalaenoptera liesselensis* differs from *Archaebalaenoptera castriarquati* and the MHNL1610 in a number of characters (see Table S8 and Fig. S5). The supraoccipital of *A. castriarquati* shows a dorsal convexity at its anterior end (Fig. S5); we use the term ‘dome’ for this convexity in the following text. Instead of the dome, *A. liesselensis* shows a dorsally concave dorsal surface with an elongated and transversely narrow external occipital crest. The infraorbital region of the frontal of *A. liesselensis* lacks the elongated and triangular narial process observed in *A. castriarquati* as the frontonasal suture of the former is approximately straight (along the transverse axis) and that of the latter is obliquely oriented (from a posterolateral point to an anteromedial point being the anteromedial point located much more anteriorly than the posterolateral point). In *A. castriarquati* the anterior end of the parietal terminates at a protrusion located on the descending portion of the frontal just medial to the emergence of the supraorbital process of the frontal. *Bisconti (2007)* called this protrusion a ‘boss’ and noted that such a boss is also present in other balaenopterid species (e.g., *Balaenoptera acutorostrata*). The boss is absent in *A. liesselensis* and the anterior end of the parietal is not anteriorly bounded by any detectable structure located in the frontal bone. The posterolateral protrusion of the exoccipital of *A. liesselensis* is more marked than that observed in *A. castriarquati* as a distinctive concavity is present between the anterolateral corner of the exoccipital and the squamosal (Fig. S5) in *A. liesselensis* but in *A. castriarquati* the continuity between the exoccipital and the squamosal is more evident in dorsal view. In *A. castriarquati* the nuchal crest does not overwhelm the medial wall of the temporal fossa but in *A. liesselensis* the temporal crest protrudes more laterally than in *A. castriarquati* and overwhelms the medial wall of the temporal fossa.

With respect to the other putative *Archaebalaenoptera* from Peru (MHNL1610), we can observe that the anterior end of the supraocciptal is almost squared in MHNL1610 and round in *A. liesselensis*, the development of the temporal crest is similar to that observed in *A. castriarquati* as the temporal fossa is completely exposed in dorsal view, and that the frontonasal suture is more similar to that observed in *A. liesselensis*. More details will be described and discussed elsewhere.

### Comparisons with other balaenopterid taxa

The supraoccipital of *Archaebalaenoptera* is typical of this genus as, in dorsal view, it exhibits a marked transverse constriction, an elongated anterior portion and a short posterior portion. A transverse constriction is observed also in other balaenopterid taxa and in other mysticetes but it is characterized by different aspects in different taxa. In *Protoroqualus*, for instance, the transverse constriction is located anteriorly, very close to the anterior end of the supraoccipital and the anterior portion of the supraoccipital converges toward the longitudinal axis of the skull forming an anteriorly pointed anterior border. In *‘Balaenoptera’ cortesii* var. *portisi* the transverse constriction is much more marked and the anterior portion of the supraoccipital is transversely narrow and its anterior end is triangular; moreover, in this taxon, anteriorly to the transverse constriction, the lateral edges of the supraoccipital are more straight in dorsal view. In *Nehalaennia devossi* the supraoccipital shows a slight transverse constriction and the anterior end of the supraoccipital is wider and round. Interestingly, in *‘Balaenoptera’ bertae* the transverse constriction is located in the anterior half of the supraoccipital (resembling *Protororqualus* and the portion anterior to the constriction is short and shows an approximately squared anterior end). In modern balaenopterids the transverse constriction is usually slight (see images in Bisconti, 2007 and True, 1904) and the anterior end is often squared (*Megaptera novaeangliae* and *B. acutorostrata* are noteworthy examples in which the anterior end of the supraoccipital may be also rounded; see True, 1904). *Incakujira*, *Diunatans* and *‘Balaenoptera’ siberi* show no constriction at all.

The periotic of *A. liesselensis* shows a high suprameatal area with a strongly protruding dorsal surface. A strong dorsal protrusion is observed in *Plesiobalaenoptera quarantellii* (Bisconti, 2010). In *A. liesselensis*, the endocranial foramina are distributed in a subtle area located in the ventral-most portion of the pars cochlearis; this character is not observed in other balaenopterid taxa. The posterior process is elongated and narrow and shows a wide canal for the route of the facial nerve. In modern balaenopterid species the posterior process is flattened (*Bisconti, 2001*; (Ekdale, Berta & Deméré, 2011) and the route of the facial nerve is not marked as in *A. liesselensis*. In *Incakujira anillodefuego* the pars cochlearis is much more elongated with respect to the anteroposterior length of the anterior process Marx & Kohno, 2016). In *Fragilicetus velponi* and *Diunatans luctoretemergo* the periotic does not show the massive bulging of the dorsal surface and the anterior process is longer (Bisconti & Bosselaers, 2016; Bisconti, Munsterman & Post, 2019).

A posterior protrusion of the exoccipital is observed in a number of fossil balaenopterid taxa, in eschrichtiids and cetotheriids. *Bisconti & Bosselaers (2016)* considered this character as a primitive state in the reconstruction of the morphological transformations of the balaenopterid skull. In particular, a protruded exoccipital is observed in *Fragilicetus velponi*, *Archaebalaenoptera castriarquati* and *Eschrichtius robustus*. *Archaebalaenoptera liesselensis* and *A. castriarquati* share this character with those taxa.

In *A. liesselensis* the alisphenoid is exposed in the medial wall of the temporal fossa for a small and rectangular spot between the parietal, squamosal and pterygoid. In the living balaenopterid species, *Incakujira anillodefuego*, *Diunatans luctoretemergo , Norrisanima miocaena (Leslie, Peredo & Pyenson, 2019)* and *Fragilicetus velponi*, the alisphenoid is exposed as a very small area included between the parietal, squamosal and pterygoid. Exceptions to this pattern are the humpback whale, *Megaptera novaeangliae*, and *Nehalaennia devossi* in which the alisphenoid is not exposed at all *(True, 1904; Bisconti, Munsterman & Post, 2019)*.

## Palaeobiogeographic Analysis

### Introduction: phylogenetic relationships of *Archaebalaenopteraliesselensis*

**Figure 6:**
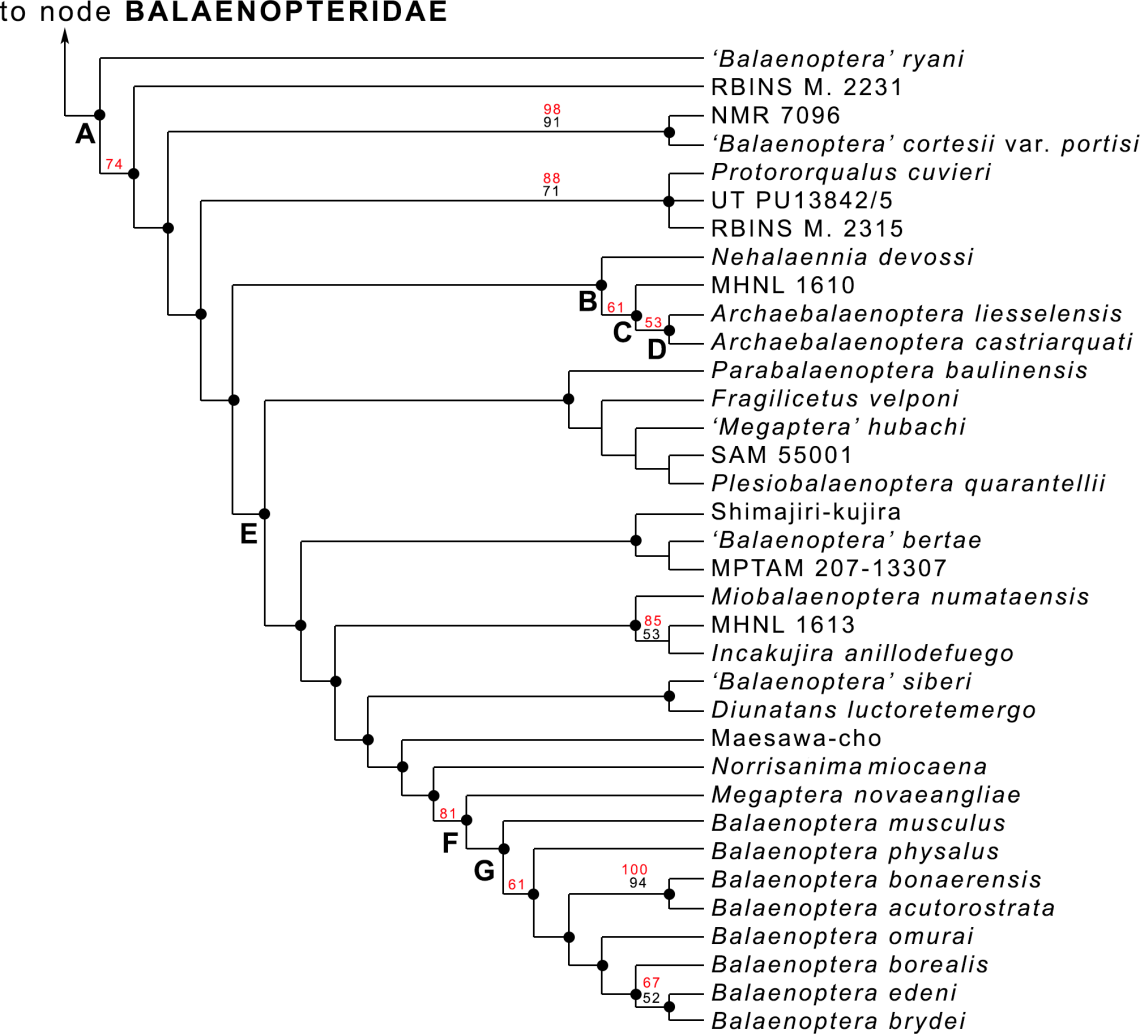
Phylogenetic relationships within Balaenopteridae. Phylogenetic relationships of Balaenopteridae modified from Bisconti, Munsterman & Post (2019). This cladogram represents an expansion of the BALAENOPTERIDAE branch of the tree of Fig. S6. Black numbers are bootstrap support values, red numbers are symmetric resampling values. See Bisconti, Munsterman & Post (2019) for explanations of methods and discussion of the branching pattern.

*Bisconti, Munsterman & Post (2019)* included the skull MAB002286 into their phylogenetic analysis of Mysticeti (general results provided in Fig. S6; balaenopterid phylogeny provided in Fig. 6; see Bisconti, Munsterman & Post, 2019 for materials, methods and resulting details of this phylogenetic analysis) with focus on Balaenopteridae and discovered that the specimen is the sister group of *Archaebalaenoptera castriarquati* (Fig. 6: node D). The clade formed by *A. castriarquati* and *A. liesselensis* is the sister group of a still undescribed *Archaebalaenoptera* species (Bisconti et al., 2020, unpublished data) from the late Miocene of Peru (Fig. 6: node C). Sister group of the genus *Archaebalaenoptera* is *Nehalaennia devossi* (Fig. 6: node B) which differs from *Archaebalaenoptera* in lacking the strong transverse compression of the supraoccipital (character No. 139 in the Bisconti, Munsterman & Post’s (2019) dataset), in having squared posterior end of the ascending process of the maxilla, in lacking the externally convex interorbital region of the frontal, and in lacking strong and protruding sites for attachment of neck muscles on the supraoccipital. All these differences justify generic differentiation between *Nehalaennia* and *Archaebalaenoptera*.

The genus *Protorqualus* is the sister group of the clade formed by *Nehalaennia, Archaebalaenoptera* and all the other advanced balaenopterid whales branching from node E of Fig. 6. *Nehalaennia* and *Archaebalaenoptera* are the sister group of more advanced balaenopterid mysticetes branching from node E (node 10 of (Bisconti, Munsterman & Post, 2019)). Symmetric resampling supporting values provided by Bisconti, Munsterman & Post (2019) were more than 50% for the monophyly of *Archaebalaenoptera* and for the sister group relationship of *A. castriarquati* and *A. liesselensis*.

### Divergence age of the *Archaebalaenoptera* clade

In Fig. S7, the phylogenetic hypothesis discussed above (Fig. 6) is plotted against a temporal scale showing the stratigraphic distribution of the fossil and recent taxa and the hypothesized diverging ages of the balaenopterid clades. While it is evident that Balaenopteridae experienced a major radiation pulse at the beginning of their history (Event 1 = Event *δ* of Bisconti, Munsterman & Post, 2019), *c.* 12–10 Ma, the First Appearance Datum (FAD) of *Archaebalaenoptera* is 8.1 Ma coinciding with the oldest estimated age of *A. liesselensis*. The age of the sister group of *Archaebalaenoptera* (i.e., *Nehalaennia*) is constrained between 8.7 and 8.1 Ma based on dinocyst analysis (Bisconti, Munsterman & Post, 2019) thus suggesting that the earliest divergence age of *Archaebalaenoptera* should occur within this time interval corresponding to the Event 2 of balaenopterid diversification detected by Bisconti, Munsterman & Post (2019). It is also inferred that the origin of the clades including genera *Incakujira*, *Miobalaenoptera*, *Parabalaenoptera*, *‘Balaenoptera’ siberi* and the Japanese specimen known as Shimajiri-kujira occurred in this same time interval (Tanaka & Watanabe, 2019; Marx & Kohno, 2016; Kimura et al., 2015; Zeigler, Chan & Barnes, 1997; Pilleri, 1989).

### Reconstructions of ancestral distributions at selected nodes

#### Fitch’s parsimony

**Figure 7:**
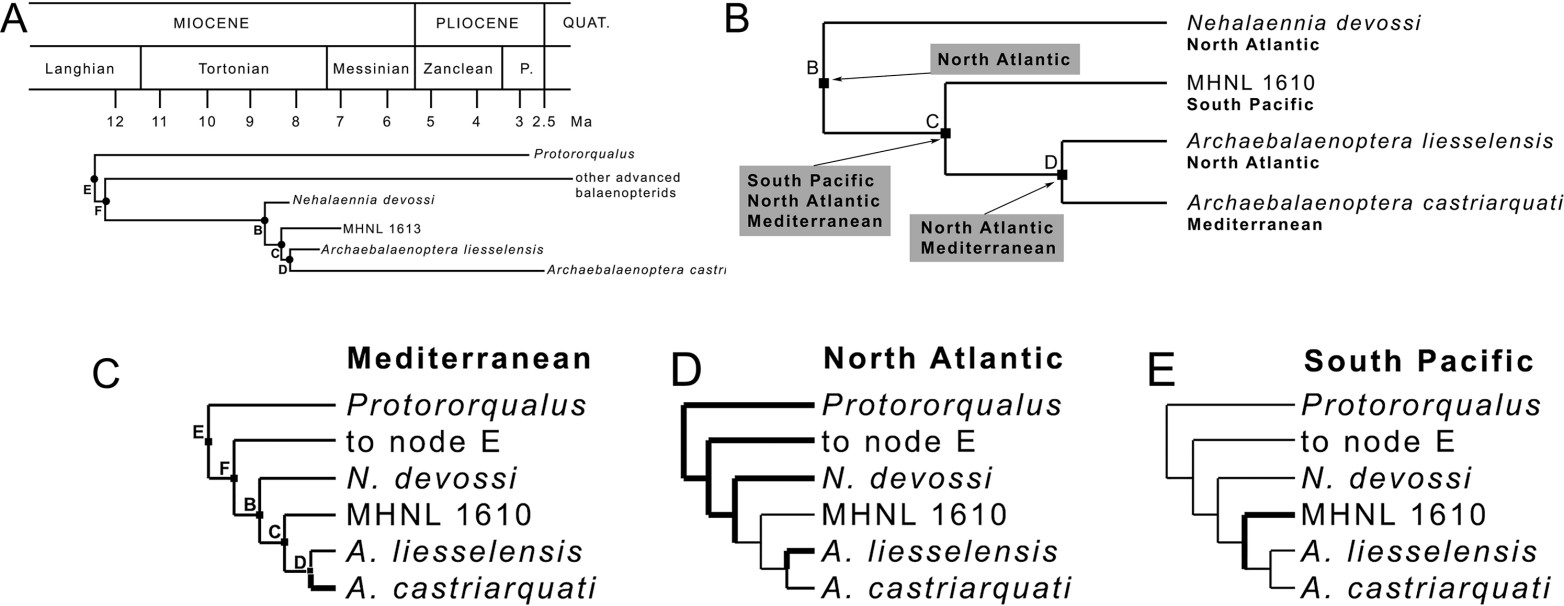
Phylogenetic relationships and paleobiogeography of the *Archaebalaenoptera* clade. (A) Phylogenetic relationships of the *Archaebalaenoptera* clade plotted against a temporal scale to show the OTUs used for the paleobiogeographic analysis. (B) Paleobiogeographic relationships of *Archaebalaenoptera* as resulting from the application of Fitch’s (1971) parsimony by hand; node letters as in (A). (C) Palaebiogeographic relationships of *Archaebalaenoptera* as resulting from the application of Maximum Parsimony by using MESQUITE; node letters as in (A).

Application of Fitch’s (1971) parsimony to the relevant clade of the present study (Fig. 7A) resulted in the inference of a series of distributions at ancestral nodes as shown in Fig. 7B. Strict application of this method results in the inference of a North Atlantic origin of the *Nehalaennia* +*Archaebalaenoptera* clade (clade B). We infer that this event occurred between 8.7 and 8.1 Ma based on the stratigraphic analysis of Fig. S7. The subsequent origin of clade C (genus *Archaebalaenoptera*) is inferred to be linked to a rapid range expansion allowing the earliest *Archaebalaenoptera* species to disperse into South Pacific and Mediterranean. This dispersal event gave rise to the *Archaebalaenoptera* species from the late Miocene of Peru (see Bisconti, Munsterman & Post, 2019 for an outline of this species). The ancestral distribution of node D included both North Atlantic and Mediterranean and is supposed to have occurred before 8 Ma. This distribution pattern suggests that the dispersal to the South Pacific was followed by rapid isolation of the southern population that quickly became a different species.

#### MP mapping of ancestral distributions at nodes

MESQUITE MP mapping of ancestral distributions at nodes of the relevant clades (Fig. 7C) was more restrictive than strict application of Fitch’s (1971) parsimony and, in the end, less useful for the paleobiogeographic history of *Archaebalaenoptera*. A North Atlantic origin is supported by this technique again for clades E, F and B but the subsequent distribution patterns may be interpreted as different dispersal events occurred within *Archaebalaenoptera*.

#### ML mapping of ancestral distributions at nodes

**Figure 8:**
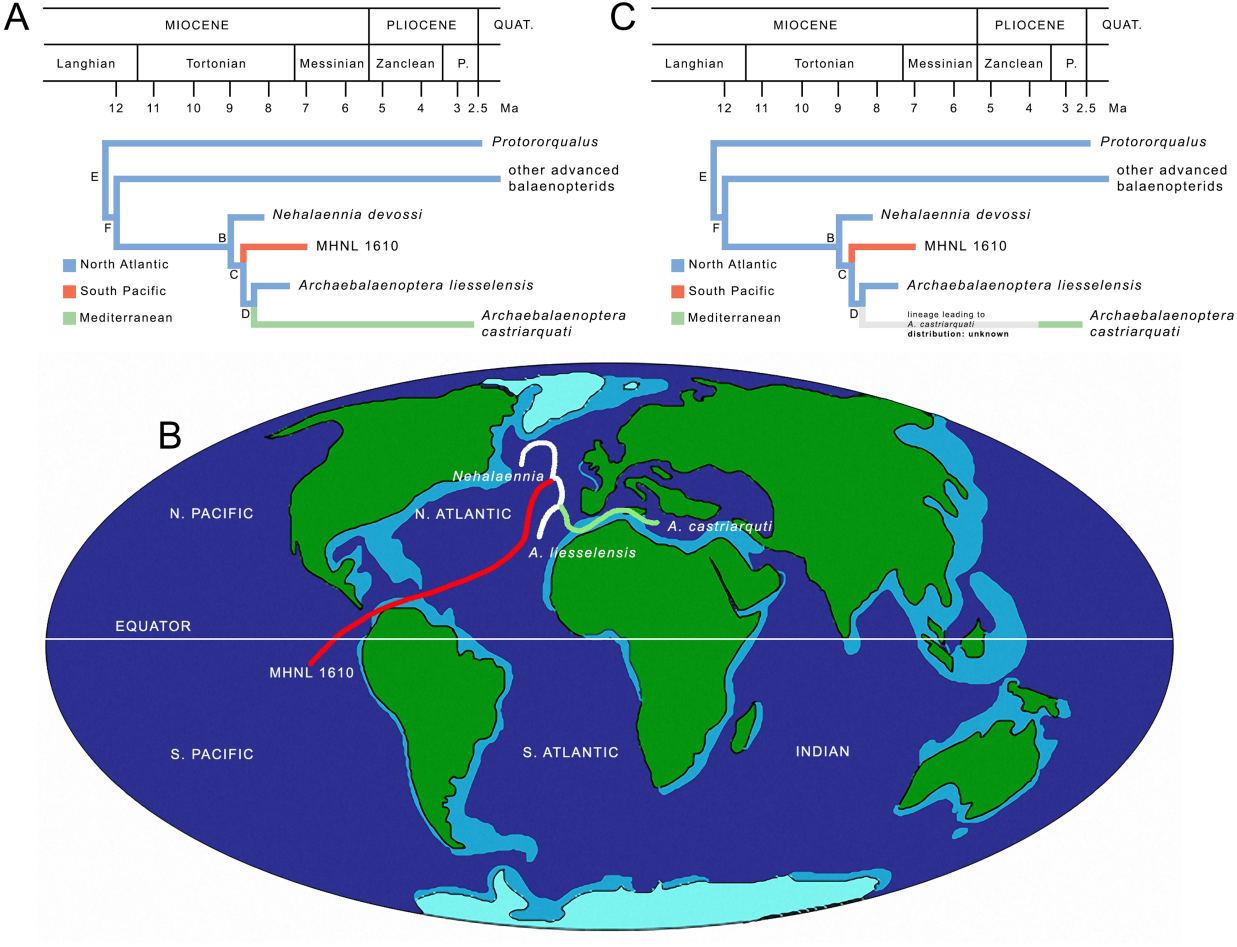
Paleobiogeography of *Archaebalaenoptera*. (A) Paleobiogeographic relationships of *Archaebalaenoptera* as resulting from the application of Maximum Likelihood by using MESQUITE; node letters as in Fig. 7A. (B) Phylogenetic relationships of *Archaebalaenoptera* species and *Nehalaennia* superimposed on a map of the mid-Miocene (modif. From Paleomap Project at http://www.scotese.com/miocene.htm) to show the dispersal events discussed in the text. (C) Revised paleobiogeographic patterns in *Archaebalaenoptera* showing unknown distributions in the branch leading to *A. castriarquati*; node letters as from Fig. 7A.

MESQUITE ML mapping of ancestral distribution at nodes of the relevant clades (Fig. 8A) gave more information. In Table S8, ML probability values for geographic occurrences at ancestral nodes are provided. It is noticeable that the North Atlantic occurrence at all the ancestral nodes received the highest ML probability values suggesting that the North Atlantic was the center of origin of all the clades and that the South Pacific and Mediterranean presence of *Archaebalaenoptera* species depended upon two separate dispersal events. The paleobiogeographic history of *Archaebalaenoptera* based on this interpretation is shown in Fig. 8B. Lower ML probability values are observed for a North Pacific origin of the node F (other advanced balaenopterid taxa), for a Mediterranean origin of node E (genus *Protororqualus*) and for a South Pacific origin of node C suggesting a more widespread distribution of the taxa branching from these nodes. However, these values are much lower than those supporting North Atlantic origins for all the ancestors of nodes E, F and C.

The ages of the paleobiogeographic events are estimated based on the combination of the information present in Fig. S7 and Table S8, and are summarized in Fig. 8B. It is interesting to note that the three rami belonging to *Archaebalaenoptera* originated in a small time interval and that the three lineages leading to the three species of this genus were already in place before 8 Ma thus suggesting an evolutionary radiation at the origin of the genus. However, as *Archaebalaenoptera liesselensis* is differentiated from *A. castriarquati* only by lacking the apomorphic characters observed in the latter, it is not possible to exclude that *A. liesselensis* is the direct ancestor of *A. castriarquati*. This means that there is a possibility that *A. castriarquati* originated at the time of its first appearance. However, as all the known specimens of *A. castriarquati* are from the Late Pliocene (Bisconti, 2007; Bisconti & Francou, 2014), and as there is a 4-million-years gap between the occurrence of *A. liessensis* and the specimens of *A. castriarquati*, it is hard to state that these specimens are contemporaneous with the origin of the species. However, as the stratigraphic occurrences of the relevant taxa are not in contrast each other, we cannot exclude that *A. liesselensis* was the direct ancestor of *A. castriarquati*. We think that a more comprehensive sample of both species with a more complete assessment of the chronological intervals of their existences are needed to better test this hypothesis.

Finally, the largely contemporaneous presence of northern (*A. liesselensis*) and southern species (the Peruvian *Archaebalaenoptera* species represented by MHNL 1610) is a clear indication of antitropical distribution of *Archaebalaenoptera* species in the late Miocene following a dispersal from a North Atlantic center of origin (Fig. 8B).

## Discussion

### On the *Archaebalaenoptera* radiation

The hypothesis of phylogeny for Balaenopteridae presented by Bisconti, Munsterman & Post (2019) shows a complex pattern of diversification within this family (Fig. 6). Several multi-species clades branch from subsequent nodes and form generic- or suprageneric-rank groups. One of these groups is the monophyletic *Balaenoptera* assemblage including only living species. From this analysis, in fact, *Megaptera* is not included within *Balaenoptera*, Eschrichtiidae is not included within Balaenopteridae and *Balaenoptera* is not paraphyletic (see Bisconti, Munsterman & Post, 2019 for additional comments and comparisons with literature).

The *Archaebalaenoptera* clade includes three species characterized by a clear transverse constriction of the supraoccipital with rounded anterior border of the supraoccipital and by externally rounded depressions laterally to the interorbital region of the frontal. Species differentiation within this clade is provided by lack of shared characters (i.e., supraoccipital dome, strong attach sites for neck muscles in the supraoccipital etc.) that does not influence the monophyly of the genus; the monophyly of *Archaebalaenoptera* is reinforced also by symmetric resampling values higher than 50%.

In a sense, the diversification of *Archaebalaenoptera* species seems a small scale evolutionary radiation. In fact, the origin of this genus (stratigraphically constrained between 8.7 and 8.1 Ma) was followed by a quick increase in diversity (shown in Fig. S7).

The *Archaebalaenoptera* radiation is part of a broader scale radiation occurred within Balaenopteridae between *c.* 9 and 7 Ma that included the origins of the Japanese Shimajiri-kujira (Kimura et al., 2015, did not report the number of this specimen that is from the Upper Miocene of the Okamishima Formation, Shimajiri Group, Miyako Island, Okinawa, Japan) and of *Incakujira*, *Miobalaenoptera* and the clade formed by *‘Balaenoptera’ siberi* and *Diunatans luctoretemergo* (Bisconti, Munsterman & Post, 2019). This diversity increase is probably linked to increased nutrient availability due to higher terrestrial erosion patterns associated to a global cooling. In fact, the West Antarctic ice sheet accumulated and ^13^C increased in this time interval thus documenting temperature decline and productivity increase (Zachos et al., 2001). Increased food availability in this period is also supported by palynological analyses in the southern North Sea Basin as reported by Bisconti, Munsterman & Post (2019) and literature therein.

Cool periods have been hypothesized working as triggers for range expansion of mysticete populations (Rosenbaum et al., 2000; McLeod, & Barnes, 1993; Davies, 1963) thus suggesting a link between the paleoenvironmental changes occurred at a global scale in the mid-late Tortonian and the quick radiation of *Archaebalaenoptera*.

### The role of the North Atlantic in balaenopterid evolution

The role played by the North Atlantic in mysticete evolution has been discussed for long time in the light of increasing knowledge about changes in current patterns occurred since the late Miocene time. In particular, *Whitmore Jr (1993)* suggested that the early appearance and establishment of modern mysticete faunas in the northern hemisphere was triggered by the accumulation of the West Antarctic ice sheet between *c.* 8 and 5 Ma. This long-term event “renewed global cooling, caused in part by a north-flowing cold bottom current whose effects eventually reached the North Atlantic” *(Whitmore, 1993, p. 225;* see also Bisconti, 2010). Global cooling associated to increased nutrient availability were concomitant factors with the balaenopterid radiation called Event 2 by *Bisconti, Munsterman & Post (2019)* between 9 and 7 Ma. This global cooling event, in our opinion, had the double effect of (1) triggering the range expansion in *Archaebalaenoptera* and (2) allowing survival of a higher number of clades within the Mysticeti as a whole. While point (1) should be the causal reason for the origin of the antitropical distribution of *Archaebalaenoptera* species, point (2) is supported by the observations of increases in clade numbers in Balaenoidea and Balaenopteridae as documented by *Bisconti, Munsterman & Post (2019)*.

Interestingly, the North Atlantic worked as a center of origin for several balaenopterid clades in the mid-late Tortonian period: Fitch’s (1971) parsimony and ML reconstructions of paleodistributions at ancestral nodes support the hypothesis that the North Atlantic was the center of origin of *Protororqualus*, *Nehalaennia*, *Archaebalaenoptera* and the common ancestor of the taxa branching from node E thus playing a major role in the evolution of balaenopterid diversity (Fig. 8B).

To test the robustness of this hypothesis we performed an experimental analysis in which a New hypothesized find (NHF) from South Atlantic is part of the *Archaebalaenoptera clade*. We applied Fitch’s (1984) parsimony to see if the NHF would add significant discrepancies with the results based on true fossil record. We made analyses in which the NEF was positioned, alternatively, as sister group of *A. castriarquati*, of *A. liesselensis* and *A. castriarquati*, and of MHNL1610, *A. liesselensis* and *A. castriarquati*. Results are shown in Fig. S8. In all cases, the origin of the *Archaebalaenoptera+Nehalaennia* clade was in North Atlantic. In all cases a massive range expansion was observed after the origin of *Archaebalaenoptera* and NHF. In all cases the subsequent range reduction events led to the origins of the different *Archaebalaenoptera* species. We conclude that a new find in South Atlantic would not significantly change the paleobiogeographic hypothesis proposed here.

However, we must admit that more comprehensive work on the paleobiogeography of extinct balaenopterid clades is necessary to fully understand the geographic aspects of the evolutionary radiations occurred in Balaenopteridae.

### The impact of the Messinian salinity crisis

A point worth discussing is the invasion of the Mediterranean basin by one *Archaebalaenoptera* species in the Pliocene. Based on the hypothesis of phylogeny adopted in the present paper, we estimate the time of the origin of the lineage leading to the Mediterranean *A. castriarquati* in about 8 Ma in the North Atlantic. At the moment, we are not able to support a hypothesis of invasion from the southern hemisphere as the published evidence does not allow us to do that. Moreover, in the last ten years, one of us (Michelangelo Bisconti) had the possibility to study a high number of fossil balaenopterids from California, South Africa and Peru (see Supplementary Information file) where he did not found any evidence of *Archaebalaenoptera-* like specimens (apart from the single specimen MHNL 1610 cited in this paper). The invasion of the Mediterranean was a subsequent event documented only in the time interval from *c.* 3.8 and 2.6 Ma (Bisconti, 2007); this paleobiogeographic pattern is shown in Fig. 8C. Unfortunately, presently, it is not possible to break the long branch leading to *A. castriarquati* mainly developed during the latest Miocene and it is not possible to assume that the evolution of *A. castriarquati* occurred entirely within the Mediterranean for two reasons. First, there is no fossil record supporting an intra-basin evolution of *Archaebalaenoptera* and, second, the Messinian salinity crisis is thought to have erased the marine biodiversity in Mediterranean starting from *c.* 5.96 Ma. As far as the fossil record is concerned, Miocene sediments from the Stirone river (northern Italy) and from the Pietra Leccese Formation (southern Italy) have been intensely investigated as well as the Neogene outcrops in Piedmont (Landini et al., 2005a; Landini et al., 2005b) without any evidence of *Archaebalaenoptera* specimens. As far as the Messinian salinity crisis is concerned, even if the dynamics of the events that led to the local extinction of the marine faunas are not yet completely understood (see Vai, 2014 for a synthesis), it is clear that for most of the late Messinian, life conditions in the Mediterranean were prohibitive for cetaceans. Interesting data are coming from Mallorca Island where a balaenopterid fossil was recently discovered that died in a phase of mitigation of the salinity crisis Mas et al., 2018a; Mas et al., 2018b). Presently, the genus- and species-level taxonomy of this specimen are not known and further investigations are highly desirable to understand the phylogenetic and paleobiogeographic relationships of this species in order to get more data about the routes that connected the Mediterranean to other basins before or during the Messinian salinity crisis. Following a similar line of reasoning, the study of a recently discovered balaenopterid from the Early Pliocene of Tuscany might help to better constrain the relationships of the Mediterranean mysticete fauna before, during and after the salinity crisis and help discover the center(s) of origin(s) of the diverse Pliocene Mediterranean balaenopterids Scotton et al., 2018).

In any case, presently, we must admit that the invasion of the Mediterranean realized by the lineage leading to *Archaebalaenoptera castriarquati* occurred in the Pliocene after the Messinian salinity crisis and independently from the inferred times of origins of the branch. This means that ghost lineages must be postulated to interpret the long evolutionary history of *Archaebalaenoptera castriarquati*. The geographic distributions of these ghost lineages are presently unknown (Fig. 8C).

## Conclusions

*Archaebalaenoptera liesselensis* is a new balaenopterid species whose description is based on a moderately well preserved skull from the southern border of the North Sea basin. The stratigraphic age of the holotype specimen is constrained between 8.1 and 7.5 Ma based on the analysis of the dinocyst assemblage present in the associated sediment. The new species differs from two additional *Archaebalaenoptera* species in details of the supraoccipital and the frontal but these small differences do not put into questions the monophyly of the genus *Archaebalaenoptera* as demonstrated by a recent phylogenetic analysis of extant and extinct Balaenopteridae (Bisconti, Munsterman & Post, 2019).

Based on the phylogenetic results of Bisconti, Munsterman & Post (2019), a paleobiogeographic analysis revealed that the center of origin of *Archaebalaenoptera* was the North Atlantic ocean where also other balaenopterid clades originated in the mid-late Tortonian. The origination pattern corresponds to the Event 2 of the analysis of balaenopterid diversity evolution of Bisconti, Munsterman & Post (2019). The trigger of this evolutionary radiation is supposed to be an increased nutrient availability due to higher erosion rates allowing more clades to survive in this time interval.

Judging from the distribution of the three *Archaebalaenoptera* species, an antitropical distribution is inferred for this genus at least during the mid-late Tortonian. In particular, the origin of a South Pacific *Archaebalaenoptera sp.* (documented by specimen MHNL 1610) is here suggested to be caused by a dispersal event from a North Atlantic center of origin during the late Tortonian. This period was characterized by global cooling due to the establishment of the West Antarctic ice sheet and cooling event are suggested to trigger range expansion in mysticete populations thus allowing *Archaebalaenoptera* individuals to cross the Equator and to settle in the South Pacific ocean.

The invasion of the Mediterranean basin by *Archaebalaenoptera* is more difficult to understand because it is usually thought that the Mediterranean salinity crisis was able to erase most of the marine biodiversity in this basin by the end of the Miocene. However, new fossils found in the latest Miocene of Mallorca and in the earliest Pliocene of Italy will hopefully shed light in the complex re-establishment of a balaenopterid fauna in the Pliocene of the Mediterranean basin.

##  Supplemental Information

10.7717/peerj.8315/supp-1 Supplemental Information 1 Supplementary Information Taxa and characters used for phylogenetic and paleobiogeographic analyses, ages of the specimens, supplementary illustrations and measurements of the holotype of *Archaebalaenoptera liesselensis.*

## Institutional abbreviations

MAB: Oertijdmuseum, Boxtel, The Netherlands
NMR: Natuurhistorisch Museum Rotterdam, Rotterdam, The Netherlands
SAM: Iziko Natural History Museum, Cape Town, South Africa
USNM: United States National Museum, Smithsonian Institution, Washington DC, United States of America

## Additional Information and Declarations

### Data Availability

The following information was supplied regarding data availability: Data is available in a Supplementary File.

### New Species Registration

The following information was supplied regarding the registration of a newly described species: Publication LSID: urn:lsid:zoobank.org:pub:41E2B619-3E94-4299-956E-CAF7237A147F Archaebalaenoptera liesselensis sp. nov. LSID: urn:lsid:zoobank.org:act:D6EF2A5E-B8C4-4D58-A141-633A72C177F2
