# Supplementary material for: A new species of rorqual whale (Cetacea, Mysticeti, Balaenopteridae) from the Late Miocene of the Southern North Sea Basin and the role of the North Atlantic in the paleobiogeography of Archaebalaenoptera
Source: PeerJ. 2020 Jan 13;8:e8315. doi: 10.7717/peerj.8315 (PMC6964694; doi:10.7717/peerj.8315)
Supplement: Supplemental Information 1 — Taxa and characters used for phylogenetic and paleobiogeographic analyses, ages of the specimens, supplementary illustrations and measurements of the holotype of Archaebalaenoptera liesselensis. [file peerj-08-8315-s001.pdf]

Supplementary Information to:

A new species of rorqual whale (Cetacea, Mysticeti, Balaenopteridae) from the Late Miocene of the Southern North Sea Basin and the paleobiogeography of *Archaebalaenoptera*

Michelangelo Bisconti[1], Dirk K. Munsterman[2], René H. B. Fraaye[3], Mark Bosselaers[4], Klaas Post[5]

[1]Dipartimento di Scienze della Terra, Università degli Studi di Torino, Torino, Italia
[2]Paleobiology Department, San Diego Natural History Museum, 1788 El Prado, 90122 CA, United States of America
[3]Toegepast Natuurwetenschappelijk Onderzoek (TNO-Netherlands Organization for Applied Scientific Research), Geological Survey of The Netherlands, P.O. Box 80015, 3508 TA, Utrecht, The Netherlands
[4] Oertijdmuseum, Bosscheweg 80, 5283 WB Boxtel, The Netherlands.
[5]Royal Belgian Institute of Natural Sciences, 29 Vautierstraat, 1000, Brussels, Belgium
[6]Het Natuurhistorisch Museum, Westzeedijk 345, 3015AA Rotterdam, The Netherlands

Corresponding Author:
Michelangelo Bisconti[1]
Dipartimento di Scienze della Terra, Università degli Studi di Torino, Via Valperga Caluso 35, 10125, Torino, Italia
Email address: michelangelo.bisconti@unito.it

Index

p. 2 *Table S1. Lithostratigraphy of the borehole B52C1978*
p. 3 *Table S2. Palynomorph assemblage*
p. 4 *Table S3. Geographic states*
p. 5 *Institutional abbreviations*
p. 6 *Specimens used in the comparative analysis*
p. 12 *Outline of undescribed specimens*
p.13 *Table S4. Geographic occurrences and ages of the taxa*
p. 16 *Table S5. Matrix for paleobiogeographic analyses*
p. 18 *Table S6. Skull measurements*
p. 19 *Table S7. Measurements of the left periotic of the holotype skull*
p. 20 *Table S8. Comparisons between* A. liesselensis, A. castriarquati and N. devossi.
p. 21 *Commented character states used in the phylogenetic analysis of Bisconti et al. (2019)*
p. 49 *Table S9.* ML results
p. 50 *Supplementary Figure S1*. Optic canal of *Archaebalaenoptera liesselensis*
p. 51 *Supplementary Figure S2*. Endocranial face of *Archaebalaenoptera liesselensis*
p. 52 *Supplementary Figure S3*. Left periotic of *Archaebalaenoptera liesselensis*
p. 53 *Supplementary Figure S4*. Medial side of the periotic of *Archaebalaenoptera liesselensis*
p. 54 *Supplementary Figure S5*. Comparative analysis of balaenopterid skulls.
p. 55 *Supplementary Figure S6*. Phylogenetic relationships of Mysticeti
p. 56 *Supplementary Figure S7*. Stratigraphic consistency of the balaenopterid phylogeny
p. 57 *Supplementary Figure S8*. Experimental analyses of paleobiogeographic relationships
p. 58 *Literature cited in the Supplementary Information*

Table S1

Lithostratigraphy of the borehole B52C1978

Lithostratigraphical and age interpretations of borehole B52C1978 (Hoogdonk).

**Depth in m MD**	**Lithostratigraphy**	**Age**	**Facies**
9.2-9.5 m	Waalre Formation	Pleistocene, Calabrian	Estuarine<br>3 % marine dinocysts, including fresh- and brackish algae
16-27 m	Breda Formation	Late Miocene, late Tortonian-Messinian	Shallow-open marine<br>16 m: 20 % dinocysts<br>22-27 m: 38-39 % dinocysts
31 m		Late Miocene, late Tortonian, Zone M14	Open marine<br>49 % dinocysts
37-44.5 m		Late Miocene, early-mid Tortonian, Zone M12	Open marine<br>32-38 % dinocysts

Table S2

Palynomorph assemblage

Palynomorph assemblage associated with the holotype of Archaebalaenoptera liesselensis.

**Dinoflagellate cysts**	**Total abundance**
Achomosphaera spp.	1
Barssidinium graminosum	1
Dinocyst indet.	1
Habibacysta tectata	3
Hystrichosphaeropsis obscura	1
Impagidinium densiverrucosum	1
Lejeunecysta spp.	3
Lingulodinium machaerophorum	6
Melitasphaeridium choanophorum	1
Operculodinium centrocarpum	10
Operculodinium janduchenei	1
Selenopemphix brevispinosa	1
Selenopemphix nephroides	1
Spiniferites spp.	15
Tectatodinium pellitum	1
**Sporomorphs**	
Bisaccates	58
Sporomorphs (excl. bisaccates)	8
**Miscellaneous fossils**	
Cyclopsiella spp.	1
Foraminifera	5

Table S3

Geographic states
Geographic states used in the paleobiogeographic analysis.

Character 1: Mediterranean
 (0) Absent
 (1) Present
Character 2: North Atlantic
 (0) Absent
 (1) Present
Character 3: South Atlantic
 (0) Absent
 (1) Present
Character 4: North Pacific
 (0) Absent
 (1) Present
Character 5: South Pacific
 (0) Absent
 (1) Present
Character 6: Indian Ocean
 (0) Absent
 (1) Present
Character 7: Arctic Polar Circle
 (0) Absent
 (1) Present
Character 8: Paratethys
 (0) Absent
 (1) Present

Institutional abbreviations

AMNH, American Museum of Natural History, New York, USA;
CASG, California Academy of Sciences, Department of Geology, San Francisco, California, USA;
ChM, The Charleston Museum, Charleston, USA;
CM, Condom Museum, University of Oregon, Eugene, Oregon; USA;
GNHM, Gamagori Natural History Museum, Gamagori, Japan;
GMNH, Gunma Museum of Natural History, Gunma, Japan;
KMNH, Kitakyushu Museum of Natural History and Human History, Kitakyushu, Japan;
LACM, Natural History Museum Los Angeles County, Los Angeles, California, USA
MAB, Oertijdmuseum Boxtel, Bosscheweg 80, 5283 WB Boxtel, The Netherlands;
MAUL, Museo dell'Ambiente, Università di Lecce, Lecce, Italy;
MB, Museum f¨ur Naturkunde, Humboldt–Universitat zu Berlin;
MGB, Museo Geopalaeontologico 'G. Capellini', Bologna, Italy;
MCA, Museo Geopalaeontologico 'G. Cortesi', Castell'Arquato, Italy;
MHNL, Museo de Historia Natural, Lima, Peru;
MLP, Museo de La Plata, La Plata, Argentina;
MNHL, Muséum national d'Histoire naturelle, Paris, France;
MPTAM, Ente Gestione Aree Protette Artigiane, Asti, Italy and Museo Paleontologico Territoriale dell'Astigiano e del Monferrato, Asti, Italy;
MRSN, Museo Regionale di Scienze Naturali, Torino, Italy;
MSM, Museum Sønderjylland, Department Natural History and Palaeontology, Gram, Denmark;
MSNT, Museo di Storia Naturale del Territorio, Calci, Italy;
MPST, Museo Palaeontologico, Salsomaggiore Terme, Italy;
NBC, Naturalis Biodiversity Center, Leiden, Holland.
NFL, Numata Fossil Museum, Hokkaido, Japan;
NHG, Natuurlijke Historie Genootschap, Zeeuws Museum, Middelburg, The Netherlands;
NMNH-P, Academician V.A. Topachevsky Paleontological Museum of the National Museum of Natural History of the National Academy of Sciences;
of Ukraine, Kiev, Ukraine;
NMB, NatuurMuseum Brabant, Tilburg, Holland;
NMR, Natuurhistorisch Museum, Rotterdam, Holland;
NMV, Museum Victoria Palaeontology Collection, Melbourne, Australia;
NSMT, National Science Museum, Tokyo, Japan;
OU, Otago University, Dunedin, New Zealand;
PIN, A.A. Borisyak Paleontological Institute, Russian Academy of Sciences, Moscow, Russia;
RBINS, Royal Belgian Institute of Natural Sciences, Brussels, Belgium;
SDNHM, San Diego Natural History Museum, San Diego, California, USA;
SKKC, Suginami Kagaku Kyoiku Center, Tokyo;
SMSN, Staatliches Museum für Naturkunde, Stuttgart, Germany;
UCMP, Museum of Paleontology, University of California, Berkeley, California, USA;
UM, University of Michigan Museum of Paleontology, Ann Arbor, Michigan, USA;
USNM, United States National Museum of Natural History, Smithsonian Institution, Washington, DC, USA;
UWBM, Burke Museum of Natural History and Culture, University ofWashington, Seattle,WA, USA;
ZMA, Instituut voor Systematiek en Populatiebiologie/Zoölogisch Museum, Amsterdam, Holland (the zoological and paleontological collections of ZMA recently moved to NBC).

Specimens used in the comparative analysis

The specimens listed below were examined by one or all the authors. In some cases, it was not possible to directly examine the specimens; in those cases, the relevant literature is provided in the list below. The list includes 8 undescribed taxa that are included in a phylogenetic analysis for the first time in this paper. These taxa are from Italy (MPTAM 207.13307 and UT PU13842/5), Belgium (RBINS M. 2231, M. 2315, NMR 7096, MAB002286) and Peru (MHNL 1610 and 1613). All these new taxa are balaenopterids and their publications are in progress. Relative ages of the species listed below are from the Cetacea section of the Paleobiology Database mostly compiled by Mark Uhen and, for undescribed taxa, from the cited literature.

1. Protocetidae
We compiled the matrix by using the following taxa:
(i) Protocetus atavus: SMNS 11084 (holotype); middle Eocene.
(ii) Georgiacetus vogtlensis: Hulbert *et al*. (1996), Hulbert (1998); middle Eocene.
(iii) Maiacetus inuus: Gingerich et al. (2009); middle Eocene.
(iv) Gaviacetus razai: Luo & Gingerich (1998); middle Eocene.

2. *Basilosaurus cetoides*
USNM 4674, 6087 as described by Kellogg (1936); Uhen (1998); late Eocene.

3. *Cynthiacetus peruvianus*
MNHN.F.PRU10 (holotype) as described in Martinez-Caceres & Muizon (2017); late Eocene-to-early Oligocene.

4. Dorudon atrox
UM 101215, 101222, 100139, 93220 as described by Uhen (2004); late Eocene.

5. *Zygorhiza kochii*
USNM 4748, 16638, 449538; Kellogg (1936), Uhen (1998); late Eocene.

6. *Aetiocetus weltoni*
UCMP 122900 (holotype) as described in Barnes *et al.* (1994), Deméré & Berta (2008); late Oligocene.

7. Mammalodontidae
We compiled the matrix by using the following taxa:
(i) Mammalodon colliveri NMV P199986 (holotype) as described in Fitzgerald (2010); late Oligocene.
(ii) Janjucetus hunderi; NMV P216929 (holotype) as described in Fitzgerald (2006); late Oligocene.

8. Fucaia buelli
UWBM 84024 (holotype) as described in Marx et al. (2015); early Oligocene.

9. Waharowa ruwhenua
OU 22044 (holotype) as described in Boessenecker & Fordyce (2015); late Oligocene.

10. Yamatocetus canaliculatus
KMNH VP 000,017 (holotype) as described in Okazaki (2012); late Oligocene.

11. *Micromysticetus rothauseni*
ChM PV4844 (holotype), Sanders & Barnes (2002a); late Oligocene.

12. *Eomysticetus whitmorei*
ChM PV4253 (holotype), Sanders & Barnes (2002b); late Oligocene.

13. *Horopeta umarere*
OU21982 (holotype) as described in Tsai & Fordyce (2015); late Oligocene.

14. *Sitsqwayk cornishorum*
UWBM 82916 (holotype) as described in Peredo & Uhen (2016); late Oligocene.

15. *Morenocetus parvus*
MLP 5–11 (holotype) as described in Buono et al. (2018): early Miocene.

16. *Caperea marginata*
AMNH AMO 36692; RBINS 1536; Baker (1985), Beddard (1901); Recent.

17. *Miocaperea pulchra*
SMNS 46978 (holotype); Bisconti (2012); late Miocene.

18. *Balaena mysticetus*
USNM 257513; ZML 1680, 3997, 2563, 2001; Bisconti (2003), Burns *et al.* (1993), Reeves & Leatherwood (1985); Recent.

19. *Balaenula astensis*
MSNT MC CF 35 (holotype); Bisconti (2000); early Pliocene.

20. *Balaenella brachyrhynus*
Natuurmuseum Brabaant (Tilburg), specimen 42001 (holotype); Bisconti (2005); early Pliocene.

21. *Eubalaena glacialis*
AMNH 42752, 256803, 90241; MSNT 264; USNM 267612, 3339990, 23077, 301637; Bisconti (2003), Cummings (1985a), True (1904); Recent.

22. *Tiucetus rosae*
MNHN.F. PPI261 (holotype) as described by Marx et al. (2017); middle-to-late Miocene.

23. *Pelocetus calvertensis*
USNM 11976 (holotype); Kellogg (1965); middle Miocene.

24. *'Aglaocetus' patulus*
USNM 13472; Kellogg (1968c); middle Miocene.

25. *Uranocetus gramensis*
MSM p 813 (holotype) as described by Steeman (2009); middle-to-late Miocene.

26. *Isanacetus laticephalus*
MFM 28501 (holotype) as described by Kimura & Ozawa (2002); early Miocene.

27. *Joumocetus shimizui*
GMNH-PV-2401 (holotype) as described by Kimura & Hasegawa (2010); late Miocene.

28. *Parietobalaena palmeri*
AMNH 128885; USNM 10677, 16570, 24883, 10909; Kellogg (1968d); middle Miocene.

29. ***Parietobalaena campiniana***
RBINS M.399-R.4018 (holotype); Bisconti *et al.* (2013); middle Miocene.

30. ***Diorocetus hiatus***
USNM 16783 (holotype), 205990; Kellogg (1968b); middle Miocene.

31. USNM 187416; middle Miocene.

32. ***Herpetocetus morrowi***
UCMP 129450 (holotype), SDNHM 65781, SDNHM 130390, SDNHM 34155, as described by El Adli et al. (2014); late Pliocene.

33. ***Piscobalaena nana***
MNHN SAS 892, 1616-1618, 1623, 1624, PPI 259, PPI 260 as described by Bouetel & De Muizon (2006); late Miocene-to-early Pliocene.

34. ***Cetotherium rathkei***
PIN 1840/1 (type) as described by Pilleri (1986) and Gol'Din (2014); middle Miocene.

35. ***Cetotherium riabinini***
NMNH-P 668/1 (holotype) as described by Gol'Din et al. (2014); late Miocene.

36. ***Mixocetus elysius***
LACM 3882 (holotype) as described by Kellogg (1934b); late Miocene.

37. ***Metopocetus hunteri***
NMR 9991-07729; Marx et al. (2015); late Miocene.

38. ***Metopocetus durinasus***
USNM 60460 (holotype); Kellogg (1968a); late Miocene.

39. ***Herentalia nigra***
NMR ZMA 5069; Bisconti (2015); late Miocene.

40. ***Cophocetus oregonensis***
CM UO 305 (holotype) as described by Packard & Kellogg (1934); early Miocene.

41. Aglaocetus moreni
MLP 5-14 (holotype) as described byKellogg (1934a); early Miocene.

42. Thinocetus arthritus
USNM 23794 (holotype) as described by Kellogg (1969a); late Miocene.

43. Halicetus ignotus
USNM 23636 (holotype) as described Kellogg (1969b); late Miocene.

44. ***Eschrichtius robustus***
AMNH 181374, 34260, 1750 ('*Eschrichtius cephalum*'), A; NMB 42001; USNM 364969, 364580, 571931, 364969, 364977, 364970, 364973, 504305; ZML St20350, St13130, 630. Andrews (1914).

45. ***Eschrichtioides gastaldii***
MGPT 13802 (holotype); Bisconti (2008); early Pliocene.

45. ***Archaeschrichtius ruggieroi***
MAUL 230/1; Bisconti & Varola (2006); late Miocene.

46. ***Titanocetus sammarinensis***
MGB 9073 1CMC172 (1-6) (holotype); Bisconti (2006); middle Miocene.

47. ***'Balaenoptera' ryani***
CASG 1733 (holotype); Hannah & McLellan (1924); late Miocene.

48. ***Archaebalaenoptera castriarquati***
holotype (inventory of the Soprintendenza per i Beni Archeologici dell'Emilia Romagna item No. 240536; MCA): Bisconti (2007a); late Pliocene.

49. ***Protororqualus cuvieri***
Specimen lost; data as described byBisconti (2007b); late Pliocene.

50. ***'Balaenoptera' cortesi* var. *portisi***
PU13803 (holotype); Sacco (1890); Portis (1884); early Pliocene.

51. ***Plesiobalaenoptera quarantellii***
holotype (inventory of the Soprintendenza per i Beni Archeologici dell'Emilia Romagna item No. 240505; MPST); Bisconti (2010); late Miocene.

52. ***Parabalaenoptera baulinensis***
CASG 66660 (holotype) as described by Zeigler *et al*. (1997); late Miocene.

53. ***Fragilicetus velponi***
NMR 999100007727; Bisconti & Bosselaers (2016); early Pliocene.

54. UT PU13842/5
Caretto (1970); early Pliocene.

55. ***Miobalaenoptera numataensis***
NFL 18 (holotpe) as described by Tanaka & Watanabe (2019); late Miocene.

56. Shimajiri-kujira
No given number; only specimen described by Kimura et al. (2015); late Miocene.

57. Maesawa-cho
No given number; only specimen described by Oishi (1984); early Pliocene.

58. ***'Megaptera' hubachi***
MB Ma 28570; Dathe (1983); Bisconti (2011); middle Pliocene.

59. ***Megaptera novaeangliae***
AMNH 24679; MSNT 263; USNM 269982, 486175 (1-2), 13656/16252, 21492; ZMA 14964, 14953 (1-2), 14952 (1-2), 14965, 14966, 14967; Winn & Reichley (1985); Recent.

60. ***Diunatans luctoretemergo***
NHG 22279 holotype; Bosselaers & Post (2010); early Pliocene.

61. ***'Balaenoptera' siberi***
No given number; only specimen described by Pilleri (1989); late Miocene.

62. ***'Balaenoptera' bertae***
UCMP 219078 (holotype) as described by Boessenecker (2013); early-to-late Pliocene.

63. ***Norrisanima miocaena***
USNM 10300 (holotype) as described by Kellogg (1925) and Leslie et al. (2019); Late Miocene.

64. ***Balaenoptera omurai***
NSMT-M32505 as described byWada et al. (2003); Yamada et al. (2008); Recent.

65. ***Balaenoptera acutorostrata***
AMNH 181411, 35680; RBINS 1537; MSNT 260, 261; ZMA 12873; Stewart & Leatherwood (1985), True (1904); Recent.

66. ***Balaenoptera bonaerensis***
SKKC 71]2793, 71]2883, AY69B, AY69A as described by Omura (1975); Recent.

67. ***Balaenoptera physalus***
AMNH 35026, 256796; MSNT 251, 252, 253, 258, 255, 257; ZMA 14950 (1-2), 14927 (1-2), 14935 (1-2), 23353, 14947; Gambell (1985*a*); Recent.

68. ***Balaenoptera musculus***
AMNH 234949, 256797, 256798; MSNT 250; ZMA 23356, 23354, 23355, 14946, 14942, 14961; Yochem & Leatherwood (1985), True (1904); Recent.

69. ***Balaenoptera edeni***
USNM 504692, 236680 (1-3); Cummings (1985b); Recent.

70. ***Balaenoptera brydei***
NBC Reg. 4003; NBC RGM 17712; Yamada et al. (2008); Recent.

71. ***Balaenoptera borealis***
USNM 504699, 504698, 504701, 504244, 486174; Gambell (1985b); Recent.

72. ***Nehalaennia devossi***
NMR 14035; this work; late Miocene.

73. MPTAM 207.13307
Bisconti et al. (in prep. a); early Pliocene.

74. NMR 7096
Bisconti & Bosselaers (in prep. a); late Pliocene.

75. RBINS M. 2231
Bisconti & Bosselaers (in prep. b); early Pliocene.

76. RBINS M. 2315
Bisconti & Bosselaers (in prep. c); early Pliocene.

77. MAB002286
Bisconti et al. (in prep b); Late Miocene.

78. SAM55001
Govender et al. (2017); late Miocene.

79. *Incakujira anillodefuego*
GNHM Fs-098-12 (holotype) as described by Marx & Kohno (2016); late Miocene.

80. MHNL 1610
Bisconti et al. (in prep. c); late Miocene.

81. MHNL 1613
Bisconti et al. (in prep. d); early Pliocene.

Outline of undescribed specimens

MPTAM 207-13307
This specimen represents a new genus and species of Balaenopteridae whose description is now complete. It includes an incomplete skull with periotic still in articulation and part of the postcrania. The estimated age is earliest Piacenzian.

NMR 999100007096
This specimen includes skull, periotic and part of the postcrania. Its morphology suggests a close relationship to 'Balaenoptera' portisi. In the remainder of the paper and in the illustrations it is called NMR 7096. The estimated age is early Piacenzian.

RBINS M. 2231
This specimen includes skull, periotics, dentaries and part of the postcrania. It is closely related to *'Balaenoptera' sibbaldina* of which it represents the first reasonably complete skeleton. The specimen is briefly presented in Bisconti & Bosselaers (2014) and a full description is currently in progress. The specimen is currently held by RBINS. The estimated age is Early Pliocene.

RBINS M. 2315
The specimen includes partial skull and postcrania of a Pliocene individual very close to *Protororqualus cuvieri*. The description of this specimen is currently in progress.

MHNL 1613
The specimen includes a large skull with periotics still in articulation. It represents a new balaenopterid genus characterized by wide exposure of parietal at the cranial vertex. A description is currently close to be finished. The estimated age is Late Miocene.

MHNL 1610
The specimen includes a partially prepared skull with fragments of dentary. Its morphology suggests close relationships with *Archaebalaenoptera castriarquati* of which it could be an additional species. A description is currently in progress. The estimated age is Late Miocene.

Table S4

Geographic occurrences and ages of the taxa

Stratigraphic and geographic data for the taxa used in the analyses.

Taxon name	Estimated stratigraphic range		Areas of occurrence	References
Protocetus atavus	48.6	40	Mediterranean	Paleobiology Database
Georgiacetus vogtlensis	40.4	37.2	North Atlantic	Paleobiology Database
Gaviacetus razai	48.6	40.4	Indian	Paleobiology Database
Maiacetus inuus	48.6	40.4	Indian	Paleobiology Database
Basilosaurus cetoides	37.2	33.9	Mediterranean North Atlantic	Paleobiology Database
Cynthiacetus peruvianus	37.2	33.9	South Pacific	Paleobiology Database
Dorudon atrox	37.2	33.9	Mediterranean, North Atlantic	Paleobiology Database
Zygorhiza kochii	37.2	33.9	North Atlantic	Paleobiology Database
Mammalodon colliveri	28.4	23.03	South Pacific	Paleobiology Database
Janjucetus hunderi	23.9	27	South Pacific	Fitzgerald (2006)
Fucaia buelli	33.9	31	North Pacific	Marx et al. (2015)
Aetiocetus weltoni	28.4	23.3	North Pacific	Paleobiology Database
Waharoa ruwhenua	27.3	20.43	South Pacific	Boessenecker & Fordyce (2015)
Yamatocetus canaliculatus	28.4	23.3	North Pacific	Paleobiology Database
Eomysticetus whitmorei	28.4	23.3	North Atlantic	Paleobiology Database
Micromysticetus rothauseni	33.9	28.4	North Atlantic	Paleobiology Database
Horopeta umarere	27.3	25.2	South Pacific	Tsai & Fordyce (2015)
Sitsqwayk cornishorum	28.4	23.03	North Pacific	Peredo & Uhen (2016)
Morenocetus parvus	20.03	15.97	South Atlantic	Buono et al. (2018)
Miocaperea pulchra	11.608	7.246	South Pacific	Bisconti (2012)
Caperea marginata	0.012	0.0	South Pacific	Paleobiology Database
Balaenella brachyrhynus	5.3	5.0	North Sea	Bisconti (2005)
Balaena mysticetus	0.012	0.0	North Atlantic, North Pacific	Paleobiology Database
Eubalaena glacialis	0.012	0.0	North Atlantic	Paleobiology Database
Balaenula astensis	3.4	3.2	Mediterranean	Bisconti (2000)
Titanocetus sammarinensis	15.97	13.81	Mediterranean	Bisconti (2006)
Tiucetus rosae	13.65	7.246	South Pacific	Paleobiology Database
Metopocetus hunteri	11.608	7.246	North Sea	Paleobiology Database
Cophocetus oregonensis	20.43	15.97	North Pacific	Paleobiology Database
Aglaocetus moreni	20.43	15.97	South Atlantic	Paleobiology Database
Mixocetus elysius	11.608	7.246	North Pacific	Paleobiology Database
Uranocetus gramensis	11.608	7.246	North Sea	Paleobiology Database
Isanacetus laticephalus	20.43	15.97	North Pacific	Kimura and Ozawa (2002)
Metopocetus durinasus	15.97	13.65	North Atlantic	Paleobiology Database
Diorocetus hiatus	15.97	13.65	North Atlantic	Paleobiology Database
'Aglaocetus' patulus	15.97	13.65	North Atlantic	Paleobiology Database
Parietobalaena palmeri	15.97	13.65	North Atlantic	Paleobiology Database
Pelocetus calvertensis	15.97	13.65	North Atlantic	Paleobiology Database
Joumocetus shimizui	11.608	7.246	North Pacific	Kimura and Hasegawa (2010)
Parietobalaena campiniana	15.0	13.2	North Sea	Bisconti et al. (2013)
USNM 187416	17.0	15.0	North Atlantic	Gottfried et al. (1994)
Piscobalaena nana	11.608	4.0	South Pacific	Paleobiology Database
Herpetocetus morrowi	3.6	2.6	North Pacific	Paleobiology Database
Cetotherium riabinini	11.6	7.246	Paratethys	Paleobiology Database
Cetotherium rathkei	13.65	7.246	Mediterranean (Paratethys)	Paleobiology Database
Thinocetus arthritus	13.65	11.608	North Atlantic	Paleobiology Database
Halicetus ignotus	13.65	11.608	North Atlantic	Paleobiology Database
Herentalia nigra	11.608	7.246	North Sea	Paleobiology Database

Archaeschrichtius ruggieroi	11.0	7.5	Mediterranean	Bisconti & Varola (2006)
Eschrichtioides gastaldii	5.0	3.0	Mediterranean	Bisconti (2008)
Eschrichtius robustus	0.1	0.0	North Sea, North Atlantic, North Pacific	Paleobiology Database
'Balaenoptera' ryani	11.608	7.246	North Pacific	Paleobiology Database
RBINS M. 2231	5.0	5.0	North Sea	Bisconti & Bosselaers in prep. a
MPTAM 207.13307	3.6	3.2	Mediterranean	Bisconti et al. in prep.
Archaebalaenoptera castriarquati	3.8	2.558	Mediterranean	Bisconti (2007a); Freschi & Cau (2015)
Protororqualus cuvieri	3.1	3.0	Mediterranean	Bisconti (2007b); Freschi & Cau (2015)
'Balaenoptera' cortesii var. *portisi*	3.6	2.588	Mediterranean, North Atlantic, North Pacific	Deméré et al. (2005)
SAM PQL-55001	5.3	5.0	South Atlantic	Govender et al. (2016)
Plesiobalaenoptera quarantellii	11.608	7.246	Mediterranean	Bisconti (2010)
'Balaenoptera' bertae	3.35	2.5	North Pacific	Boessenecker (2013)
Parabalaenoptea baulinensis	7.246	5.332	North Pacific	Zeigler et al. (1997)
Fragilicetus velponi	5.332	5.0	North Sea; South Atlantic	Bisconti and Bosselaers (2016); Govender (2019)
'Megaptera' hubachi	5.332	3.6	South Pacific	Bisconti (2011)
Diunatans luctoretemergo	5.3	2.558	North Sea	Bosselaers & Post (2010)
'Balaenoptera' siberi	7.246	5.332	South Pacific	Paleobiology Database
MHNL 1610	8.0	7.0	South Pacific	Bisconti et al. (in prep. a)
MHNL 1613	7.5	7.3	South Pacific	Bisconti et al. (in prep. b)
UT PU13842/5	3.4	3.2	Mediterranean	Caretto (1970)
Archaebalaenoptera liesselensis	8.2	7.5	North Sea	Bisconti et al. (in prep. c)
RBINS 2315	3.71	2.74	North Sea	Bisconti & Bosselaers (in prep. b)
NMR 7096	3.7	2.7	North Sea	Bisconti & Bosselaers (in prep. c)
Incakujira anillodefuego	7.5	7.3	South Pacific	Marx & Kohno (2016)
Megaptera novaeangliae	0.781	0.0	North Atlantic, North Pacific, South Atlantic, South Pacific, Indian Ocean	Paleobiology Database;
'Balaenoptera' bertae	5.332	2.558	North Pacific	Boessenecker (2013)
Miobalaenoptera numataensis	6.8	6.5	North Pacific	Tanaka & Watanabe (2019)
Norrisanima miocaena	7.6	7.3	North Pacific	Leslie et al. (2019)
Maesawa-Cho	5.3	5.0	North Pacific	Oishi et al. (1985)
Shimajirikujira	9.0	8.0	North Pacific	Kimura et al. (2015)
Balaenoptera borealis	2.6	0.0	North Atlantic, North Pacific, South Atlantic, South Pacific, Indian Ocean	Paleobiology Database
Balaenoptera edeni	0.012	0.0	North Atlantic, North Pacific, South Atlantic, South Pacific, Indian Ocean	Paleobiology Database
Balaenoptera musculus	1.806	0.0	North Atlantic, North Pacific, South Atlantic, South Pacific, Indian Ocean	Paleobiology Database
Balaenoptera omurai	0.012	0.0	North Pacific	Paleobiology Database
Balaenoptera brydei	0.012	0.0	North Pacific	Wada et al. (2007)
Balaenoptera physalus	1.3	0.0	North Atlantic, North Pacific, South Atlantic, South Pacific, Indian Ocean, Mediterranean	Paleobiology Database
Balaenoptera acutorostrata	3.6	0.0	North Atlantic, North Pacific, Mediterranean,	Paleobiology Database

Balaenoptera bonaerensis	0.012	0.0	Indian Ocean<br>South Atlantic, South Pacific	Paleobiology Database

FO, first occurrence; LO, last occurrence. Data in Ma.

Table S5
Matrix for paleobiogeographic analyses
Taxon x character matrix used in paleobiogeographic analyses

**Taxon**	**characters**<br>12345678
Protocetidae	11000100
Cynthiacetus peruvianus	00001000
Basilosaurus cetoides	11000000
Dorudon atrox	11000000
Zygorhiza kochii	01000000
Mammalodontidae	00001000
Fucaia	00010000
Aetiocetus weltoni	00010000
Yamatocetus canaliculatus	00010000
Eomysticetus whitmorei	01000000
Micromysticetus rothauseni	01000000
Waharowa ruwhenua	00001000
Sitsqwayk cornishorum	00010000
Horopeta umarere	00001000
Morenocetus parvus	00100000
Caperea marginata	00101000
Miocaperea pulchra	00001000
Balaenella brachyrhynus	01000000
Balaena mysticetus	00000010
Balaenula	11010000
Eubalaena	01111000
'Aglaocetus' patulus	01000000
Pelocetus calvertensis	01000000
Uranocetus gramensis	01000000
Isanacetus laticephalus	00010000
Joumocetus shimizui	00010000
Parietobalaena palmeri	01000000
Parietobalaena campiniana	01000000
Tiucetus rosae	00001000
Diorocetus hiatus	01000000
USNM187416	01000000
Mixocetus elysius	00010000
Cetotherium rathkii	00000001
Cetotherium riabinini	00000001
Metopocetus durinasus	01000000
Metopocetus hunteri	01000000
Piscobalaena nana	00001000
Herpetocetus morrowi	00010000
Herentalia nigra	01000000
Thinocetus arthritus	01000000
Halicetus ignotus	01000000
Titanocetus sammarinensis	10000000
Cophocetus oregonensis	00010000

Aglaocetus moreni	00100000
Eschrichtius robustus	01010000
Eschrichtioides gastaldii	10000000
Archaeschrichtius ruggieroi	10000000
RBINS M. 2231	01000000
'Balaenoptera' ryani	00010000
Protororqualus cuvieri	10000000
RBINS M. 2215	01000000
UT PU13842/5	10000000
'Balaenoptera' cortesii var. *portisi*	11010000
NMR 7096	01000000
MPTAM 207-13307	10000000
Plesiobalaenoptera quarantellii	10000000
SAM 55001	00100000
Parabalaenoptera baulinensis	00010000
Fragilicetus velponi	01100000
'Megaptera' hubachi	00001000
Incakujira anillodefuego	00001000
MHNL 1613	00001000
Archaebalaenoptera castriarquati	10000000
MHNL 1610	00001000
Archaebalaenoptera liesselensis	01000000
Nehalaennia devossi	01000000
'Balaenoptera' bertae	00010000
Shimajirikujira	00010000
Maesawa-cho	00010000
Miobalaenoptera numataensis	00010000
Diunatans luctoretemergo	01000000
Norrisanima miocaena	00010000
'Balaenoptera' siberi	00001000
Megaptera novaeangliae	01111100
Balaenoptera musculus	01111100
Balaenoptera physalus	01111100
Balaenoptera acutorostrata	01010000
Balaenoptera bonaerensis	00101100
Balaenoptera omurai	00010000
Balaenoptera brydei	00010000
Balaenoptera edeni	01111100
Balaenoptera borealis	01111100

Table S6

Skull measurements

Measurements of the skull of Archaebalaenoptera liesselensis (MAB002286, holotype). Data in mm.

**Character**	**measurements**		
	**left**	**central**	**right**
Skull length[1] measured ventrally		398	
Maximum width of skull at external corners of exoccipitals (by doubling the distance between left exoccipital and longitudinal axis of skull)		330	
Maximum width of skull at posterior apices of lambdoid crest (by doubling the distance between the left apex and the longitudinal axis of skull)		410	
Maximum height of skull[1]		225	
Maximum width of interorbital region of frontal		113	
Maximum length of interorbital region of frontal	7.3	20.6	5.6
Length of base of supraorbital process of frontal[1]	212		159
Maximum dorsoventral height of base of supraorbital process of frontal[1]	61		56
Maximum dorsoventral diameter of alisphenoid	40		
Maximum anteroposterior diameter of alisphenoid	34.8		
Length of interparietal		8	
Width of interparietal		45	
Length of supraoccipital[1]		310	
Anterior width of supraoccipital[1]		95	
Maximum width of supraoccipital across transverse constriction (by doubling the distance between left border and longitudinal axis of skull)		225	
Maximum distance between posterolateral corner of exoccipital and external border of occipital condyle	165		
Dorsoventral diameter of occipital condyle	62		
Lateromedial diameter of occipital condyle	105		
Length of basisphenoid and basioccipital		131	
Maximum width of basisphenoid and basioccipital		105	
Maximum length of vomer		210	
Maximum width of vomer		160	

[1]As preserved.

Table S7

Measurements of the left periotic of the holotype skull

Linear measurements of the left periotic of the holotype skull of Archaebalaenoptera liesselensis *(MAB002286). Data in mm.*

**Character**	**measurement**
Posterior process: length	135
Posterior process: width at proximal end	22
Posterior process: width at mid-length	21
Posterior process: width at distal end	12
Posterior process height at distal end	47
Posterior process: anteroposterior diameter at distal end	17
Anterior process: estimated length	48[3]
Anterior process: proximal width + MEA[1] + LTA[2]	65
Anterior process: proximal width + LTA[2]	54
Anterior process: proximal width	37[3]
Pars cochlearis: anteroposterior diameter	37
Pars cochlearis: transverse diameter	55
Oval window: dorsoventral diameter	3.5
Oval window: anteroposterior diameter	7.3

[1]Medial eminence of anterior process

[2]Lateral tuberosity of anterior process

[3]Estimated value

Table S8

Comparisons between *A. liesselensis*, *A. castriarquati* and *N. devossi*.

Comparisons between Archaebalaenoptera liesselensis *and related species (*A. castriarquati *and* Nehalaennia devossi*).*

**Character**	***Archaebalaenoptera castriarquati***	***Archaebalaenoptera liesselensis***	***Nehalaennia devossi***
Dome on anterior portion of supraoccipital	yes	no	no
Elongated supraoccipital anteriorly to transverse constriction	yes	yes	no
Transversely rounded depressions lateral to the interorbital region of the frontal	yes	yes	no
Strongly developed attach sites for neck muscles on supraoccipital	yes	no	no
External occipital crest reaching anterior border of supraoccipital	no	yes	yes
Transverse constriction of supraoccipital	strong	strong	weak
Narial process	long	absent	short
Anterior border of supraorbital process of the frontal anteriorly concave	no	?	yes

Character list

The following character list is developed from the morphological dataset of *Bisconti et al. (2019)*. In the present dataset, selected character states were commented in order to warrant clear understanding. In defining character states, we made use of personal observations on specimens listed above and of literature. In particular, we need to cite the following papers that we used for character definitions and codings: Boessenecker & Fordyce (2015); Fordyce & Marx (2012); Steeman (2009); Geisler & Sanders (2003); Kimura & Ozawa (2001); Benke (1993); Kellogg (1923); Miller (1925).

ROSTRUM: PREMAXILLA, MAXILLA, NASAL

1) Rostrum length:
(0) Rostrum length shorter or equal to neurocranium length;
(1) Rostrum length longer than neurocranium length.

2) Rostrum width:
Comment: character coded 0 in archaeocetes and Balaenidae; all other mysticetes are coded 1.
(0) Rostrum narrow;
(1) Rostrum wide.

3) Rostrum straight:
Comment: character coded 1 only in Balaenidae and Eschrichtiidae.
(0) Yes;
(1) No, rostrum highly arched.

4) Rostrum arc:
Comment: character coded for Balaenidae only; code 0 is for Balaena *and* Balaenella*; code 1 is for* Eubalaena *and* Balaenula.
(0) Continuous;
(1) Discontinuous.

5) Mesorostral groove:
(0) Absent;
(1) Present.

6) Ventral keel along rostrum:
(0) Absent;
(1) Present.

7) Premaxilla widens at anterior end:
(0) No;
(1) Yes.

8) Premaxillary foramen:
(0) Present;
(1) Absent.

9) Posterior end of premaxilla:
(0) More anterior than frontonasal suture;
(1) At posterior end of nasal;
(2) Anterior to nasal.

10) Sutural contact between rostrum and frontal limited to ascending process of the maxilla:
(0) No;
(1) Yes.

11) Premaxilla and frontal articulation:
(0) Sutured;
(1) Not sutured.

12) External surface of maxilla:
(0) Sub-vertical;
(1) Sub-horizontal.

13) Medial border of maxilla anterior to narial fossa:
(0) Straight;
(1) Sinuous.
14) Lateral border of maxilla:
(0) Uniformly concave;
(1) Straight;
(2) Uniformly convex;
(3) Sinuous
15) Thickness of lateral border of maxilla:
Comment: Chaeomysticeti and Eomysticetidae are coded 1 when rostrum is preserved.
(0) Thin;
(1) Thick.
16) Lateral process of maxilla:
(0) Absent;
(1) Present.
17) Length of lateral process of maxilla:
Comment: a very long lateral process of the maxilla is observed in those taxa where this structure is longer than the transverse diameter of the maxilla at the level of the antorbital notch; a long lateral process is observed in those taxa where this structure is longer 50% of the transverse diameter of the maxilla at the level of the antorbital notch but is shorter than the whole transverse diameter.
(0) Short;
(1) Long.
(2) Very long.
18) Position of external apex of lateral process of maxilla:
(0) Anterior to antorbital corner of orbit;
(1) Anterior and medial to orbit.
19) Infraorbital process of maxilla:
(0) Absent;
(1) Present.
20) Ascending process of maxilla:
(0) Absent;
(1) Present.
21) Width of ascending process of maxilla relative to its length:
(0) Narrow;
(1) Wide.
22) Length of ascending process of maxilla:
Comment: Balaenidae, Neobalaenidae and basal thalassotherian taxa are coded 0; Eschrichtiidae, Cetotheriidae and Balaenopteridae are coded 1.
(0) Short;
(1) Long;
23) Lateral border of ascending process of maxilla:
(0) Forms an evident corner with posterior border of maxilla;
(1) Forms a wide curve with posterior border of maxilla.
24) Position of posterior ends of ascending processes of maxillae:
(0) Posterior ends do not meet along midline;
(1) Posterior ends meet along midline.
25) Meeting of ascending processes of the maxillae along the longitudinal axis of the skull:
(0) Contact limited to posterior corners;
(1) Contact extended to most of medial borders of the ascending processes of the maxillae.

26) Shape of posterior end of ascending process of maxilla at adulthood:
(0) Triangular;
(1) Squared;
(2) Rounded.
27) Shape of posterior end of ascending process of maxilla during late ontogeny:
(0) Triangular;
(1) Squared;
(2) Rounded.
28) Lateral and medial borders of ascending process of maxilla:
(0) Anteriorly diverging;
(1) Parallel;
(2) Anteriorly converging.
29) Position of posterior end of maxilla:
(0) Anterior to nasal;
(1) At level of posterior end of nasal;
(2) Posterior to nasal.
30) Position of posterior ends of maxillae:
Comment: posterior ends of maxillae are transversely far if the nasals and premaxillae have wide transverse diameter. For instance, state 0 is present in living Balaenoptera *species and in Balaenidae; state 0 is present in early-diverging balaenopterids such as* Protororqualus *and in basal thalassotherian taxa where the transverse diameter of the nasals is massively shortened; state 2 is present in Cetotheriidae.*
(0) Transversely far;
(1) Transversely close;
(2) Transversely very close.
31) Numerous dorsal infraorbital foramina:
(0) Absent (only one foramen is present);
(1) Present.
32) Location of dorsal infraorbital foramina:
(0) Scattered along dorsal surface of maxilla;
(1) Mostly located close to the medial border of maxilla.
33) Medial border of maxilla:
(0) not relieved;
(1) relieved and forming a crest.
34) Antorbital notch:
(0) Absent;
(1) Present.
35) Shape of antorbital notch:
(0) Concavity in anterior edge of lateral process of maxilla without medial-projecting groove;
(1) Developed along medial-projecting groove.
36) Articulation between maxilla and frontal:
(0) Tight;
(1) Loose.
37) Maxillary pocket:
(0) Absent;
(1) Present.
38) Infraorbital plate visible in dorsal view:
(0) No;
(1) Yes.

39) Teeth at adulthood in maxilla and premaxilla:
(0) Present;
(1) Absent.
40) Grooves for vasculature of baleen epithelium:
(0) Absent;
(1) Present.
41) Fissure located along posterior border of maxilla in ventral view:
(0) Absent;
(1) Present.
42) Elongation of fissure:
Comment: character coded in Balaenidae and Neobalaenidae only; state 0 is present in Neobalaenidae; state 1 is present in Balaenidae.
(0) Fissure short;
(1) Fissure long.
43) Nasal length:
(0) Nasal reaching the anterior 20% of rostrum;
(1) Nasal reaching approximately rostrum midlength;
(2) Nasal reaching the posterior 20% of rostrum;
(3) Nasal reaching a point close to the anterior border of the supraorbital process of frontal.
(4) Nasal reaching a point located within the interorbital region of the frontal.
44) Anterior border of nasal:
(0) Concave;
(1) Straight;
(2) Convex.
45) Median keel in nasal:
(0) Absent;
(1) Present.
46) Position of anterolateral corner of nasal:
(0) Anterior to anteromedial corner;
(1) Lateral to anteromedial corner;
(2) Posterior to anteromedial corner.
47) Position of frontonasal suture:
(0) At anterior border of interorbital region of frontal;
(1) Well within interorbital region of frontal.
48) Nasal borders:
(0) With a concavity at midlength
(1) Converging anteriorly;
(2) Parallel-to-subparallel;
(3) Diverging anteriorly.
49) Nasal width:
(0) Nasal transversely wide;
(1) Nasal with strong transverse compression along its entire length.

FRONTAL
50) Shape of supraorbital process of frontal:
(0) Flat and forming a dorsal shield;
(1) descending from interorbital region of frontal;
51) Diversity of depressions:
(0) No depression;
(1) Gentle depression from interorbital region of frontal;
(2) Abrupt depression from interorbital region of frontal;

52) Cross-sections of depressions:
(0) No depression;
(1) Triangular;
(2) Laterally concave;
(3) Squared;
(4) Half-circle.
53) Anteroposterior length of supraorbital process of frontal:
Comment: very long anteroposterior length of the supraorbital process of the frontal is observed in Balaenopteridae; a long anteroposterior length is observed in Eschrichtiidae and some Cetotheriidae while all the other mysticetes are coded 0.
(0) Short;
(1) Long;
(2) Very long.
54) Transverse diameter of supraorbital process of frontal with respect to length of neurocranium:
Comment: a short diameter of supraorbital process of frontal with respect to the length of neurocranium is observed in archaeocetes and and early toothed mysticetes; state 1 is observed in Eomysticetidae, basal thalassotherian taxa, neobalaenids and some cetotheriids; state 2 is observed in Balaenidae and Balaenopteridae.
(0) Short;
(1) Long;
(2) Very long.
55) Anterior border of supraorbital process of frontal:
(0) Directed posteriorly;
(1) Directed transversely;
(2) Directed anteriorly.
56) Anterior border of supraorbital process of frontal:
(0) Straight;
(1) Convex;
(2) Concave.
57) Backing of central and distal portions of the anterior border of the supraorbital process of frontal from its anteromedial corner:
(0) Absent;
(1) Present.
58) Posterior border of supraorbital process of frontal:
(0) Uniformly concave;
(1) Medial concavity;
(2) Straight.
59) Posterior border of supraorbital process of frontal:
(0) Directed posteriorly;
(1) Directed transversely;
(2) Directed anteriorly.
60) Supraorbital foramina:
(0) Present;
(1) Absent.
61) Orbitotemporal crest:
(0) Along posterodorsal edge of supraorbital process of frontal;
(1) From postorbital corner to anteromedial end of supraorbital process of frontal;
(2) Forming a curve from postorbital corner onto dorsal surface of supraorbital process of frontal;
(3) Forming a curve along anterior edge of supraorbital process of frontal.

62) Orbitotemporal crest:
(0) Well developed and sharp;
(1) Well developed and rounded;
(2) Highly reduced to a line.
63) Superimposition of parietal on interorbital region of frontal:
(0) Absent;
(1) Present.
64) Long superimposition of posteromedial elements of rostrum on interorbital region of frontal:
(0) Absent;
(1) Present.
65) Posterior border of interorbital region of frontal:
(0) In contact with parietal;
(1) In contact with supraoccipital.
66) Shape of coronal (frontal-parietal) suture:
(0) Straight;
(1) Anteriorly convex;
(2) Anteriorly concave.
67) Coronal suture in dorsal view:
(0) Visible;
(1) Not visible because superimposed by the supraoccipital.
68) Frontal encircles ascending process of maxilla:
(0) No;
(1) Yes.
69) Postorbital process and zygomatic process of squamosal:
Comment: state 0 is observed in those taxa where there is a long space between the anterior end of the zygomatic process of the squamosal and the postorbital process. State 1 is observed in those taxa where the space between the zygomatic process and the postorbital process is strongly reduced and these structures are almost in contact.
(0) Far;
(1) Close;
(2) Superimposed and articulated by dedicate facet.
70) Location of optic canal in ventral surface of supraorbital process of frontal:
(0) Along anterior three-fourth;
(1) Along posterior one-fourth.
71) Length of intertemporal constriction:
Comment: state 0 is observed in archaeocetes and Eomysticetidae; state 1 is observed in basal thalassotherian taxa; state 2 is observed in Cetotheriidae and Eschrichtiidae; state 3 is observed in Balaenidae, Neobalaenidae and Balaenopteridae.
(0) Very long;
(1) Long;
(2) Short;
(3) Very short.
72) Transverse diameter of intertemporal constriction:
Comment: state 0 is observed in archaeocetes and eomysticetiids; state 1 is observed in basal thalassotherian taxa and cetotheriids; state 2 is observed in balaenids, neobalaenids, eschrichtiids and balaenopterids.
(0) Highly constricted;
(1) Moderately constricted;
(2) Wide.

73) Presence of narial process:
(0) Present;
(1) Absent.
74) Length of narial process relative to nasal length:
Comment: the narial process is coded 0 if the anteroposterior length is less than the transverse width and 1 if the anteroposterior length is longer or equal to the transverse width;
(0) Short;
(1) Long.
75) Shape of narial process:
(0) The narial processes form a triangle in dorsal view;
(1) The narial processes form a bilobated protrusion in dorsal view.

PARIETAL
76) Location of frontal border of parietal:
(0) Posterior to posterior apex of ascending process of maxilla;
(1) Anterior to posterior apex of ascending process of maxilla.
77) Anterolateral corner of parietal (for Balaenidae only):
(0) Sharp;
(1) Broad.
78) Anterior portion of external surface:
(0) Visible in dorsal view;
(1) Not visible in dorsal view because overhanged by temporal crest.
79) Posterior portion of external surface:
(0) Visible in dorsal view;
(1) Not visible in dorsal view because overhanged by temporal crest.
80) Post-parietal foramen:
(0) Present;
(1) Absent.
81) Parietal spreading onto emergence of supraorbital process of frontal:
(0) Absent;
(1) Present.
82) Parietal exposed at cranial vertex:
(0) Yes;
(1) No.
83) Length of parietal exposure at vertex:
Comment: state 0 is observed in archaeocetes, Eomysticetidae; state 1 is observed in basal thalassotherian taxa; state 2 is observed in Cetotheriidae and Eschrichtiidae; state 3 is observed in Balaenopteridae.
(0) Long;
(1) Moderate;
(2) Short;
(3) Very short.
84) Sagittal crest at cranial vertex:
(0) Present;
(1) Absent.
85) Attach for temporalis muscle at intertemporal constriction:
Comment: state 0 corresponds to a transversely narrow sagittal crest; state 1 corresponds to a sagittal crest with expanded dorsal surface (as observed, for instance, in Titanocetus sammarinensis); state 2 is observed in Cetotheriidae and Eschrichtiidae; state 3 is observed in Balaenopteridae, Balaenidae and Neobalaenidae.
(0) Very narrow;

(1) Slightly widened;
(2) Moderately widened;
(3) Wide.
86) Shape of sagittal crest:
(0) Sharply-edged;
(1) Forming two opposite concavities.
87) Tubercle at lambdoid suture:
(0) Absent;
(1) Present.
88) Parietal-squamosal suture:
(0) Sinuous;
(1) Straight.

SQUAMOSAL
89) Dorsoventral height of squamosal:
Comment: high dorsoventral height of squamosal in lateral view is observed only in Balaenidae and Neobalaenidae.
(0) Low dorsoventral height;
(1) High dorsoventral height.
90) Anteroposterior length of zygomatic process of squamosal with respect to its height:
Comment: a very long zygomatic process of the squamosal is observed in archaeocetes, Aetiocetidae and Eomysticetidae; state 1 is observed in basal thalassotherian taxa and Balaenopteridae; state 2 is observed in Cetotheriidae and Eschrichtiidae; state 3 is observed in Balaenidae and Neobalaenidae.
(0) Very long;
(1) Long;
(2) Short.
(3) Very short.
91) Height of zygomatic process of squamosal:
(0) Zygomatic process higher than postglenoid process;
(1) Zygomatic process at the same level of postglenoid process;
(2) Zygomatic process much higher than postglenoid process.
92) Projection of anterior portion of zygomatic process of squamosal in dorsal view:
(0) Projecting anteromedially;
(1) Projecting anterolaterally;
(2) Projecting anteriorly.
93) Projection of posterior portion of zygomatic process of squamosal in dorsal view:
(0) Projecting anteromedially;
(1) Projecting anterolaterally
(2) Projecting anteriorly.
94) Zygomatic process of squamosal in dorsal view:
(0) Anteriorly straight;
(1) Anteriorly twisted.
95) Distinctive articular facet for postorbital process of frontal on zygomatic process of squamosal:
(0) Absent;
(1) Present.
96) Projection of apex of zygomatic process in lateral view:
(0) Anterior;
(1) Ventral.

97) Postglenoid process of squamosal:
(0) Projecting ventrally;
(1) Projecting posteroventrally.
98) Twisted postglenoid process of squamosal:
(0) No;
(1) Yes.
99) Lateral surface of squamosal:
(0) Smooth;
(1) With single fossa for sternomastoid muscle;
(2) With double fossa for sternomastoid muscle.
100) Anteroposterior concavity along dorsolateral edge of glenoid fossa of squamosal:
(0) Absent;
(1) Present.
101) Glenoid fossa of squamosal:
(0) Forming a right angle in lateral view;
(1) Slightly concave;
(2) Highly concave (half-moon shaped);
(3) Straight.
102) Location of glenoid fossa of squamosal:
0) posterior to orbit;
1) immediately posteroventral to orbit.
103) Height of squamosal at nuchal crest:
(0) Low;
(1) High.
104) Supramastoid crest:
(0) Present;
(1) Absent.
105) Orientation of supramastoid crest:
(0) Dorsal;
(1) Anterior.
106) Nuchal crest in dorsal view:
Comment: state 0 corresponds to a nuchal crest with wide and round shape; state 1 corresponds to a nuchal crest with round but narrow shape; state 2 corresponds to a triangular nuchal crest.
(0) Wide;
(1) Narrow;
(2) Very narrow.
107) Nuchal crest in dorsal view:
(0) Circular;
(1) Triangular.
108) Nuchal crest in dorsal view:
(0) Reaching a point anterior to occipital condyle;
(1) Reaching a point posterior to occipital condyle;
(2) Reaching a point at the same level as occipital condyle.
109) Squamosal bulging into temporal fossa:
(0) No;
(1) Yes.
110) Extension of temporal fossa with respect to total skull length:
Comment: state 0 is observed in archaeocetes, Aetiocetidae and Eomysticetidae; state 1 is observed in basal thalassotherian taxa; state 2 is observed in Cetotheriidae, Balaenidae, Neobalaenidae and Balaenopteridae.

(0) Very wide;
(1) Wide;
(2) Reduced.
111) Extension of temporal fossa:
(0) Longer than wide;
(1) Wider than long.
112) Shape of temporal fossa in dorsal view:
(0) Oval;
(1) Almond-shaped;
(2) Triangular.
113) Surface of temporal fossa anterior to nuchal crest:
(0) More horizontal than ventral-most portion;
(1) Developed dorsoventrally.
114) Squamosal cleft:
(0) Absent;
(1) Present.
115) Shape of squamosal cleft:
(0) Straight;
(1) Triangular.
116) Length of squamosal cleft:
Comment: state (0) < 50 mm; (1) between 51 and 70 mm; (2) longer than 70 mm.
(0) Short;
(1) Long;
(2) Very long.
117) Origin of squamosal cleft at adulthood:
(0) From parietal-squamosal suture;
(1) From parietal-squamosal-alisphenoid suture;
(2) From squamosal-alisphenoid suture;
(3) From squamosal-pterygoid suture.
118) Origin of squamosal cleft during late ontogeny:
(0) From parietal-squamosal suture;
(1) From parietal-squamosal-alisphenoid suture;
(2) From squamosal-alisphenoid suture;
(3) From squamosal-pterygoid suture.
119) Infundibulum of Foramen ovale:
(0) Absent;
(1) Present.
120) Foramen ovale:
Comment: definitions of complete and incomplete infundibulum are from Fraser and Purves (1960).
(0) Infundibulum complete;
(1) Infundibulum incomplete.
121) Foramen ovale:
(0) Located within squamosal;
(1) Located between squamosal and pterygoid.
(2) Located within pterygoid.
122) Suture present in foramen ovale:
(0) No;
(1) Yes.

123) Squamosal crease:
(0) Absent;
(1) Present.
124) Secondary squamosal crest:
(0) Absent;
(1) Present.
125) Secondary squamosal fossa:
(0) Absent;
(1) Present.
126) Basicranial foramina:
(0) Separate foramina in posterolateral portion of skull;
(1) Foramina confluent into a single and large posterior lacerate foramen.

SUPRAOCCIPITAL
127) Supraoccipital in dorsal view:
(0) Not visible because main development is dorsoventral;
(1) Visible because it superimposes on parietal.
128) Anteroposterior supraoccipital elongation:
(0) No anteroposterior elongation;
(1) Short: supraoccipital superimposed on posterior portion of parietal;
(2) Long: supraoccipital superimposed on most of parietal;
(3) Very long: supraoccipital superimposed on whole parietal and part of interorbital region of frontal.
129) Anteroposterior supraoccipital elongation with respect to zygomatic process of squamosal:
(0) Anterior border of supraoccipital reaching a point located more posteriorly than the anterior apex of the zygomatic process of squamosal;
(1) Anterior border of supraoccipital reaching a point located more anteriorly than the anterior apex of the zygomatic process of squamosal.
130) Shape of anterior border of supraoccipital:
Comment: state (3) is observed when a triangular anterior portion of a supraoccipital shows externally convex and rounded borders rather than straight.
(0) Round;
(1) Triangular;
(2) Squared;
(3) Ogival.
131) Distinctive articular facets for ascending process of the maxilla in anterior border of supraoccipital:
(0) Absent;
(1) Present.
132) Size of anterior border of supraoccipital:
Comment: the anterior border of the supraoccipital is wide in archaeocetes and Titanocetus.
(0) Wide;
(1) Pointed;
(2) Narrow.
133) Elevation of anterior border of supraoccipital in lateral view:
(0) High elevation formed by dorsal protrusion of parietals lateral and in front of the anterior border of supraoccipital;
(1) Low elevation without contribution by the parietal;
(2) No elevation at all.
134) Distinctive depression in front to supraoccipital in lateral view:
(0) Present;
(1) Absent.

135) Dorsal surface of supraoccipital:
(0) Concave;
(1) Flat-to convex.
136) Attach sites for neck muscle attachments:
(0) Not evident;
(1) Well developed.
137) Attach sites for neck muscle attachments:
(0) Shaped as triangular relieves with flat surface;
(1) Shaped as tubercles.
138) External occipital crest:
(0) Absent;
(1) Present.
139) Lateral borders of supraoccipital in dorsal view:
(0) Not visible;
(1) Uniformly convex;
(2) uniformly straight;
(3) uniformly concave;
(4) sinuous because of the presence of a transverse constriction.
140) Position of transverse constriction of supraoccipital:
(0) In anterior-most portion;
(1) At mid-length;
(2) In posterior half.
141) Degree of transverse constriction with respect to maximum transverse width:
Comment: scarce transverse constriction is observed in Eomysticetidae, basal thalassotherian taxa, Cetotheriidae, Eschrichtiidae and Balaenoptera*; moderate constriction is observed in* Protororqualus *and* Nehalaennia*; strong constriction is observed in* Archaebalaenoptera *and* 'Balaenoptera' cortesii *var.* portisi.
(0) Scarce;
(1) Moderate;
(2) Strong.
142) Lateral borders of supraoccipital anterior to the transverse constriction:
(0) Concave;
(1) Straight-to-convex.
143) Length of external occipital protuberance:
Comment: a short external occipital protuberance is observed in Protororqualus*; a long external occipital protuberance is observed in* 'Balaenoptera' cortesii *var.* portisi.
(0) Long;
(1) Moderate;
(2) Short.
144) Anterolateral corner of supraoccipital:
(0) Not distinguishable;
(1) Collapsed into a single anterior point;
(2) Rounded;
(3) Squared.
145) Supraoccipital bent at midlength:
(0) No;
(1) Yes.

INTERPARIETAL
146) Interparietal:
(0) Absent;

(1) Present.
147) Shape of interparietal:
Comment: as shown in Wada et al. (2003), in Balaenopteridae, the interparietal may be anteroposteriorly long and transversely narrow and anteroposteriorly short and transversely wide; characters 147 and 148 relate to this observation.
(0) Short;
(1) Long.
148) Shape of interparietal:
(0) Wide;
(1) Narrow.

JUGAL
149) Jugal elongation:
Comment: elongated and straight jugal is observed in archaeocetes.
(0) Jugal elongated and mostly straight;
(1) Jugal short and rounded.

LACRIMAL
150) Lacrimal exposed in dorsal view:
(0) No;
(1) Yes.
151) Sutured lacrimal:
(0) Yes;
(1) No.

EXOCCIPITAL
152) Exoccipital in posterior view:
(0) Anterolateral border forming a right angle with lateral edge of supraoccipital;
(1) Anterolateral border continuous with lateral edge of supraoccipital.
153) Exoccipital development in posterior view:
Comment: the transverse elongation of the supraoccipital is observed in those taxa where there is a sharp corner between the anterodorsal border of the exoccipital and the posterolateral border of the supraoccipital being the lateral portion of the exoccipital protruded laterally; this character is absent in crown mysticetes and cetotheriids.
(0) Exoccipital transversely elongated;
(1) Transverse elongation of exoccipital reduced.
154) Protrusion of posterolateral corner of exoccipital:
(0) At level of postglenoid process;
(1) Medial to postglenoid process.
155) Protrusion of posterolateral corner of exoccipital:
(0) Reaching a point more anterior than occipital condyles;
(1) Reaching a point more posterior than occipital condyles.
156) Protrusion of posterolateral corner of exoccipital:
(0) More posterior than postglenoid process of squamosal.
(1) More anterior than postglenoid process of squamosal;
157) Occipital condyle:
(0) Convex articular face;
(1) Flat-to-slightly convex articular face.
158) Neck of occipital condyle:
(0) Well developed;
(1) Indistinct.

159) Condyloid foramen:
(0) Present;
(1) Absent.
160) Foramen in jugular notch:
(0) Present;
(1) Absent.

BASIOCCIPITAL
161) Basioccipital crest:
(0) Absent;
(1) Present.
162) Fusion of medial crest of basioccipital crest and falcate process of basioccipital:
(0) Absent;
(1) Present.

ALISPHENOID
163) Alisphenoid exposure in temporal fossa:
(0) Present;
(1) Absent.
164) Size of alisphenoid exposure in temporal fossa:
(0) Large;
(1) Small;
(2) Very small.
165) Alisphenoid borders:
(0) Between frontal, parietal, squamosal and pterygoid;
(1) Between parietal, squamosal and pterygoid;
(2) Between parietal and squamosal;
(3) Between parietal and pterygoid.

PALATINE
166) Palatine reaching a point located close to posterior border of skull:
(0) No;
(1) Yes.

PTERYGOID
167) Pterygoid fossa:
(0) Absent;
(1) Present.
168) Pterygoid hamulus:
Comment: well developed pterygoid hamulus is observed only in Balaenoptera *and* Megaptera.
(0) Short;
(1) Well developed.
169) Ventral lamina of pterygoid:
(0) Absent;
(1) Present.
170) Pterygoid exposure in temporal fossa:
(0) Absent;
(1) Present.

PERIOTIC

171) Posterior process exposure in lateral wall of skull:

(0) Absent;

(1) Present.

172) Posterior process length:

Comment: a short posterior process is observed in archaeocetes, odontocetes and early-diverging chaeomysticetes (Eomysticetidae); a long posterior process is observed in extant Balaenidae and Balaenopteridae.

(0) Short;

(1) Moderate;

(2) Long.

173) Posterior process size and shape:

(0) Prismatic and robust;

(1) Transversely compressed and flattened.

174) Facial sulcus along posterior process:

(0) Absent;

(1) Present.

175) Facial sulcus along posterior process:

Comment: a long facial sulcus is developed along approximately the whole length of the posterior process otherwise it is considered short.

(0) Short;

(1) Long.

176) Position of facial sulcus on posterior process:

(0) Along medial border and hidden in ventral view;

(1) Ventromedial;

(2) Completely ventral.

177) Borders of facial sulcus:

(0) Sulcus bordered by crests;

(1) Sulcus widened and bordered by narrow relieves.

178) Facial sulus completely included in a tube-like structure:

(0) No;

(1) Yes.

179) Shape of posterior border of posterior process:

(0) Clavate;

(1) Squared;

(2) Pointed.

180) Stylomastoid fossa:

(0) Not distinguishable;

(1) Elongated and shallow;

(2) Elongated and covered by a relieved dorsal edge in the posterior process;

(3) Short and included within posterior process as a notch.

181) Anterior process:

(0) Absent;

(1) Present.

182) Anterior process length:

Comment: a short anterior process is observed when the anterior process length is less-to-equal to the posterior process length otherwise the anterior process is long.

(0) Short;

(1) Long.

183) Anterior process thickness:
Comment: a blade-like anterior process is observed in some Cetotheriidae where the anterior process is subtle in medial view; the anterior process is thick in balaenids and in all those taxa where the maximum height of the anterior process is equal-to-longer to the dorsoventral height of the pars cochlearis in medial view otherwise it is thin.
(0) Thick;
(1) Thin;
(2) Blade-like.
184) Origin of anterior process:
(0) Abruptly depressed from dorsal surface of periotic;
(1) Anterior process continuous with dorsal surface of periotic.
185) Anterior process in dorsal (or ventral) view:
(0) Squared;
(1) Irregular shape;
(2) Triangular;
(3) Elliptical.
186) If triangular, medial edge of anterior process:
(0) Convex or straight;
(1) Concave.
187) If triangular, lateral edge of anterior process:
(0) Convex or straight;
(1) Concave.
188) If triangular, apex of anterior process:
(0) Round;
(1) Pointed.
189) Lateral tuberosity:
(0) Absent;
(1) Present.
190) Size of lateral tuberosity:
(0) Small;
(1) Large.
191) Shape of lateral tuberosity:
(0) Protruding and squared or rounded;
(1) Protruding and triangular.
192) Lateral process of anterior process:
(0) Absent;
(1) Present.
193) Length of lateral process of anterior process:
Comment: the lateral process of the anterior process is long if its apex reaches the mid-length of the posterior process; if it does not reach that point then it is short. This character is coded for Balaenidae.
(0) Long;
(1) Short.
194) Shape of lateral process of anterior process:
(0) Broadly triangular;
(1) Sharply triangular.
195) Medial emergence of anterior process:
(0) Absent;
(1) Present.
196) Tensor tympani groove along anterodorsal edge of pars cochlearis:
(0) Present;
(1) Absent.

197) Dorsal surface of periotic:
(0) Highly relieved;
(1) Low.
198) Highly relieved dorsal surface of periotic:
(0) Squared;
(1) Dome-shaped.
199) Dorsal surface of periotic and anterior process forming a straight line in medial view:
(0) No;
(1) Yes.
200) Suprameatal area:
(0) Concave;
(1) Gently descending;
(2) Convex and protruding.
201) Superior process:
(0) Present;
(1) Absent.
202) Size of superior process:
(0) Convex dorsal profile in medial view;
(1) Reduced to a low ridge;
(2) Absent.
203) During late ontogeny, internal acoustic meatus including:
(0) Tractus spiralis foraminosus, foramen singulare and endocranial opening of facial canal;
(1) Tractus spiralis foraminosus and foramen singulare.
204) At adulthood, internal acoustic meatus including:
(0) Tractus spiralis foraminosus, foramen singulare and endocranial opening of facial canal;
(1) Tractus spiralis foraminosus and foramen singulare.
205) Crista transversa during ontogeny:
(0) Septum-like;
(1) Thick.
206) Crista transversa during adulthood:
(0) Septum-like;
(1) Thick.
207) Position of crista transversa at adulthood:
(0) Does not reach medial rim of internal acoustic meatus;
(1) Reaches medial rim of internal acoustic meatus.
208) Fissure in endocranial opening of facial canal during ontogeny:
(0) Absent;
(1) Present.
209) Fissure in endocranial opening of facial canal at adulthood:
(0) Absent;
(1) Present.
210) Vascular groove:
(0) Evident;
(1) Reduced;
(2) Absent.
211) Transverse elongation of pars cochlearis:
Comment: transverse elongation of the pars cochlearis is observed only in Balaenopteridae and Eschrichtiidae.
(0) Short;
(1) Elongated.

212) Anteroposterior elongation of pars cochlearis:
Comment: anteroposterior elongation of pars cochlearis is observed only in Balaenopteridae and Eschrichtiidae.
(0) Short;
(1) Elongated.
213) Inflation of pars cochlearis:
(0) Absent;
(1) Present.
214) Anterior crest along pars cochlearis:
(0) Absent;
(1) Present.
215) Cochlear window (round window) and aperture for cochlear aqueduct (endolymphatic foramen) confluent during late ontogeny:
(0) No;
(1) Yes.
216) Cochlear window (round window) and aperture for cochlear aqueduct (endolymphatic foramen) confluent at adulthood:
(0) No;
(1) Yes.
217) Cochlear window (round window) and aperture for cochlar aqueduct (endolymphatic foramen) opening in a tube-like channel:
(0) No;
(1) Yes.
218) Promontorial groove:
(0) Absent;
(1) Present.
219) Size of promontorial groove:
Comment: a large promontorial groove is observed in Plesiobalaenoptera quarantellii, 'Megaptera' hubachi *and SAM 55001.*
(0) Small;
(1) Large.
220) Endocranial opening of facial canal connected to internal acoustic meatus by a groove:
(0) No;
(1) Yes.
221) Pyramidal process:
(0) Present;
(1) Absent.

TYMPANIC BULLA
222) Shape of posterior border:
(0) Bilobated;
(1) Transversely straight;
(2) Convex;
(3) Keeled.
223) Elongation of portion posterior to conical process:
(0) Present;
(1) Absent.
224) Posterior border fissurated:
(0) Yes;
(1) No.

225) Elliptical foramen:
(0) present;
(1) absent.
226) Ventral keel:
(0) Absent;
(1) Present.
227) Ventral concavity:
(0) Present;
(1) Absent.
228) involucral protrusion in dorsal view:
(0) Absent;
(1) Present.
229) Dorsal border of involucrum in medial view:
(0) Gently descending;
(1) Not descending.
230) Position of Eustachian opening relative to overall height of tympanic bulla:
Comment: the Eustachian opening is located more ventrally in early diverging mysticetes including eomysticetids, basal thalassotherian taxa and cetotheriids; in all the other baleen-bearing mysticetes it is located at a higher position.
(0) Low;
(1) High.
231) Eustachian opening bordered anteriorly:
(0) no;
(1) yes.
232) Flat posterior dorsomedial face:
(0) No;
(1) Yes.
233) Anterolateral expansion:
(0) Absent;
(1) Present.
234) Extension of anterolateral expansion:
Comment: a short anterolateral expansion is observed in Balaenidae and Neobalaenidae.
(0) Short;
(1) Long.
235) Shape of anterolateral expansion in dorsal view:
(0) Round;
(1) Pointed.
236) Tympanic cavity with respect to length of tympanic cavity:
Comment: a low tympanic cavity is observed in Balaenidae and Neobalaenidae only.
(0) High;
(1) Low.
237) Height of tympanic bulla:
Comment: a low tympanic bulla is observed in Balaenidae and Neobalaenidae only.
(0) High;
(1) Low.
238) Anterior border:
(0) Anteriorly convex;
(1) Anteriorly straight-to-concave.

239) Sigmoid process:
(0) Anteroposteriorly elongated;
(1) Transversely elongated.
240) Conical process:
(0) High;
(1) Very reduced.
241) Proportional size of tympanoperiotic complex with respect of head size:
Comment: small-sized tympanoperiotic complex is observed in ‘Balaenoptera’ cortesii *var.* portisi *and* Incakujira anillodefuego.
(0) Large;
(1) Small.
242) Outer lip and dorsal border of involucrum:
(0) Descending parallel toward anterior end;
(1) Posteriorly diverging as the outer lip is more inclined than involucrum.

DENTARY
243) Cranio-mandibular joint:
(0) Tight;
(1) Loose.
244) Teeth on dentary at adulthood:
(0) Present;
(1) Absent.
245) Mental symphysis:
(0) Present;
(1) Absent.
246) Groove for mental ligament:
(0) Absent;
(1) Present.
247) Anterior torsion:
(0) Absent;
(1) Present.
248) Massive elongation of dentary ramus:
(0) Absent;
(1) Present.
249) Coronoid process height:
Comment: state 0 is present in archaeocetes and early mysticetes including Eomysticetidae; state 1 is observed in basal thalassotherian taxa and early-diverging Balaenopteridae; state 2 is observed in Cetotheriidae and Balaenopteridae; state 3 is observed in Neobalaenidae, Balaenidae and Megaptera novaeangliae.
(0) High;
(1) Moderately high;
(2) Low;
(3) Very low-to-absent.
250) Postcoronoid crest:
(0) Absent;
(1) Present.
251) Postcoronoid fossa:
(0) Absent;
(1) Present.

252) Size of postcoronoid fossa:
Comment: a small postcoronoid fossa is observed only in living *Balaenoptera* species.
(0) Wide;
(1) Small.
253) Satellite process:
(0) Absent;
(1) Present.
254) Size of satellite process:
(0) Large;
(1) Small.
255) Orientation of articular surface of mandibular condyle:
(0) Posterodorsal;
(1) Dorsal;
(2) Posterior.
256) Posterodorsal corner of dentary:
(0) Round;
(1) Sharp.
257) Angular process:
Comment: state 0 is observed in archaeocetes and early mysticetes including Eomysticetidae; state 1 is observed in basal thalassotherian taxa; state 2 is observed in Balaenidae, Neobalaenidae and basal balaenopterids; state 3 is present in living balaenopterids.
(0) High;
(1) Moderately high;
(2) low;
(3) Very low.
258) Angular process in lateral view:
(0) Located more anteriorly than articular surface of condyle;
(1) Rounded and not protruded.
(2) Projecting ventrally;
(3) Projecting posteriorly.
(4) Squared and not protruding.
259) Mandibular foramen:
Comment: a small mandibular foramen is observed in Balaenidae, Neobalaenidae, Balaenopteridae and Eschricthiidae.
(0) Wide;
(1) Small.
260) Shape of mandibular foramen:
(0) Posteriorly concave;
(1) Triangular;
(2) Fissurated.
261) Gingival foramina:
(0) Absent;
(1) Present.
262) Mental foramina:
(0) Only one per dentary;
(1) Several mental foramina present per dentary.
263) Dentary curvature in dorsal view:
(0) Dentary with lateral concavity in dorsal view;
(1) Dentary straight;
(2) Dentary moderately bowed;
(3) Dentary strongly bowed.

264) External curvature in dorsal view:
(0) Absent;
(1) Continuous;
(2) Discontinuous.
265) Presence of dorsoventral curvature in dentary in lateral view:
(0) Absent;
(1) Present.
266) Types of dorsoventral curvature in dentary in lateral view:
(0) Absent;
(1) Continuous;
(2) Discontinuous.
267) Mylohyoidal groove:
(0) Absent;
(1) Present.
268) Crest along the ventral border of the dentary with a parallel groove:
(0) Absent;
(1) Present.
269) Medial face of dentary ramus:
(0) Flat;
(1) Convex.
(2) Concave.

VERTEBRAE
270) Cervical vertebrae:
(0) Free;
(1) Fused.
271) Cervical vertebrae:
(0) Elongated;
(1) Shortened.
272) Neural processes of cervical vertebrae:
(0) Free;
(1) Fused.
273) Dorsal process of C3:
(0) Present;
(1) Absent.
274) Dorsal process of C4:
(0) Present;
(1) Absent.
275) Dorsal process of C5:
(0) Present;
(1) Absent.
276) Dorsal process of C6:
(0) Present;
(1) Absent.
277) Dorsal process of C7:
(0) Present;
(1) Absent.
278) Ventral process of C3:
(0) Present;
(1) Absent.

279) Ventral process of C4:
(0) Present;
(1) Absent.
280) Ventral process of C5:
(0) Present;
(1) Absent.
281) Ventral process of C6:
(0) Present;
(1) Absent.
282) Ventral process of C7:
(0) Present;
(1) Absent;
(2) Reduced to a tubercle.
283) Foramen transversarium in C3:
(0) Complete;
(1) Incomplete.
284) Foramen transversarium in C4:
(0) Complete;
(1) Incomplete.
285) Foramen transversarium in C5:
(0) Complete;
(1) Incomplete.
286) Foramen transversarium in C6:
(0) Complete;
(1) Incomplete.
287) Foramen transversarium in C7:
(0) Complete;
(1) Incomplete.
288) Foramen transversarium
(0) Complete in C2;
(1) Incomplete in C2.
289) Fusion of sacral vertebrae:
(0) Present at least in part;
(1) Absent.
290) Number of sacral vertebrae:
(0) >1;
(1) 1.
291) Sharp lateroventral projection of transverse process:
(0) Present;
(1) Absent.
292) Foramen at emergence of transverse process:
(0) In caudal vertebrae;
(1) In last lumbar and caudal vertebrae.

SCAPULA
293) General proportions of scapula:
Comment: state 0 is observed in archaeocetes and Balaenidae; state 1 is observed in all the other chaeomysticetes.
(0) High and short;
(1) Low and wide.

294) Orientation of scapular spine:
(0) Divergent from margo cranialis and directed dorsally;
(1) Parallel to margo cranialis and directed anterodorsally.
295) Development of teres fossa:
(0) Small;
(1) Enlarged.
296) Margo cranialis:
(0) Straight;
(1) Convex;
(2) Concave.
297) Inclination of margo cranialis with respect to horizontal axis:
(0) High;
(1) Scarce.
298) Margo caudalis:
(0) Straight-to-scarcely concave;
(1) Highly concave.
299) Development of supraspinous fossa:
(0) Wide;
(1) Reduced;
(2) Invisible in lateral view.
300) Scapular spine:
(0) Well developed:
(1) Reduced.

HUMERUS
Comment: anatomical terminology from Benke (1993).
301) Orientation of caput humeri:
(0) Along longitudinal axis of humerus;
(1) Located posteriorly to longitudinal axis.
302) Size of tuberculum majus:
Comment: size is assessed with respect to total humeral length: state 0 is if dorsoventral height of tuberculum majus is less than 10% of the total humeral length; state 1 is if the height is more than 15%..
(0) Small;
(1) Large.
303) Direction of tuberculum majus:
(0) Anteroposterior;
(1) Dorsal;
(2) Ventral.
304) Shape or margo ulnaris:
(0) Straight;
(1) Concave.
305) Shape of caput humeri:
(0) Flat;
(1) Highly convex.
306) Lateral edge of caput humeri:
(0) Straight;
(1) Forming a corner.
307) Orientation of lateral edge of caput humeri:
(0) Anteroposterior;
(1) Oblique (from a posterodistal to an anteroproximal position);
(2) Anteroposterior posterodistally and dorsoventral anteroproximally.

308) Lateral expansion of articular surface of caput humeri:
Comment: state 1 is observed in Balaenidae.
(0) Scarce;
(1) Well developed.
309) Deltopectoral crest:
(0) Present;
(1) Absent.
310) Tuberculum deltoideus:
Comment: state 0 is observed in those taxa where the tuberculum forms a long and evident crest; state 1 is observed in those taxa where the tuberculum is reduced to a small-sized relief.
(0) Highly relieved;
(1) Reduced;
(2) Absent.
311) Articulation with radius and ulna:
(0) Rotational;
(1) Non-rotational.
312) Position of ulnar epycondyle:
Comment: state 1 is observed in those taxa where the ulnar epycondyle is located close to the posterodistal corner of the ulna.
(0) High;
(1) Low;
(2) Almost absent.
313) Relative length of humerus:
(0) Longer than radius and ulna;
(1) Humerus length nearly equals that of radius and ulna;
(2) Much shorter than radius and ulna.
314) Proximal surface of tuberculum deltoideus:
(0) Continuous with deltopectoral crest;
(1) Concave;
(2) Straight and projecting posteriorly.

RADIUS
315) Proximal curvature:
(0) Massive;
(1) Reduced-to-absent.
316) Distal expansion:
(0) Absent;
(1) Present.
317) Proximal contact with ulna:
(0) Present;
(1) Absent.
318) Size of radius with respect to ulna:
(0) Anteroposterior diameter similar to that of ulna;
(1) Anteroposterior diameter larger than that of ulna.

ULNA
319) Olecranon: proximal corner:
(0) Directed proximally;
(1) Directed distally.

320) Olecranon: size:
(0) Well developed;
(1) Reduced.
321) Olecranon: dorsal and ventral borders:
(0) Parallel;
(1) Diverging posteriorly;
(2) Forming a right angle.
322) Olecranon: ventral angle:
(0) Right angle-to-obtuse;
(1) Acute.
323) Olecranon: posterior border:
(0) Squared;
(1) Round;
(2) Straight.
324) Proximal articular facet of ulna and upper side of olecranon:
(0) Forming a corner;
(1) Straight.
325) Distal expansion of ulna:
(0) Absent;
(1) Present.

MANUS
326) Articulation of carpals:
(0) Tight articulation;
(1) Loose articulation.
327) Digit number:
(0) Five;
(1) Four.
328) Hyperphalangy:
(0) Absent;
(1) Present.
329) Proportions of manus:
(0) Manus wide;
(1) Manus narrow.
330) Trapezium:
(0) Present;
(1) Absent.
331) Separate cartilagineous fields for trapezoid and unciform:
(0) Yes;
(1) No.

HINDLIMB
332) Pelvis articulated with vertebral column:
(0) Yes;
(1) No.
333) Massive reduction of pelvis size:
(0) No;
(1) Yes.

334) Functional hindlimbs in adults:
(0) Yes;
(1) No.

STERNUM AND RIBS
335) Number or ribs articulated to sternum:
(0) >1;
(1) 1.
336) First rib shape:
(0) Not expanded;
(1) Expanded.
337) Sternum formed by several sternebra:
(0) Yes;
(1) No, only by one manubrium.
338) Head of first rib:
(0) Bifid;
(1) Single.
339) Ribs with bifid head posterior to 5th:
(0) Yes;
(1) No.
340) Pachyosteoschlerotic ribs:
(0) Absent;
(1) Present.

DENTITION
341) Positions of upper premolars and molars:
(0) Close to each other;
(1) Well separated by diastemata.
342) Positions of lower premolars and molars:
(0) Close to each other;
(1) Well separated by diastemata.
343) Number of denticles on posterior upper teeth:
(0) >3 along anterior or posterior borders;
(1) 3 or less along anterior or posterior borders.
344) Dental generations:
(0) Polyophiodonty;
(1) Monophiodonty.
345) Heterodont teeth on dentary:
(0) Present;
(1) Absent.
346) Dentition reduced to a few anterior upper teeth:
(0) No, complete dentition is present;
(1) Yes.
347) Inferred or observed loss of mineralization in teeth (due to C4*orf* gene mutation):
(0) Absent;
(1) Present.

BALEEN
348) Inferred or observed presence of baleen:
(0) Negative;

(1) Positive.

349) Inferred or observed length of baleen:

Comment: long baleen are observed or inferred in Balaenidae and Neobalaenidae.

(0) Short;

(1) Long.

350) Direction of baleen racks:

(0) Limited to posterior part of rostrum;

(1) Parallel;

(2) Anteriorly convergent.

Table S9

ML results

Maximum likelihood values for reconstructions of biogeographic presence at selected nodes.

Node[1]	Medit.[1]	N. Atl.[1]	S. Atl.[1]	N. Pac.[1]	S. Pac.[1]	Indian[1]	Arctic[1]	Parat.[1]
E	0.100	**0.485**	0.000	0.071	0.003	0.000	0.000	0.000
F	0.012	**0.477**	0.000	0.133	0.007	0.000	0.000	0.000
B	0.003	**0.600**	0.000	0.034	0.023	0.000	0.000	0.000
C	0.010	**0.351**	0.000	0.015	0.150	0.000	0.000	0.000
D	0.093	**0.433**	0.000	0.010	0.019	0.000	0.000	0.000

[1]Caption: Medit, Mediterranean basin; N. Atl., North Atlantic; S. Atl., South Atlantic; N. Pac., North Pacific; S. Pac., South Pacific; Indian, Indian Ocean; Arctic, Artic Polar Circle; Parat., Paratethys. Bold: higher probability values; underlined values are probability values immediately lower than the bold values.

Supplementary Figure S1

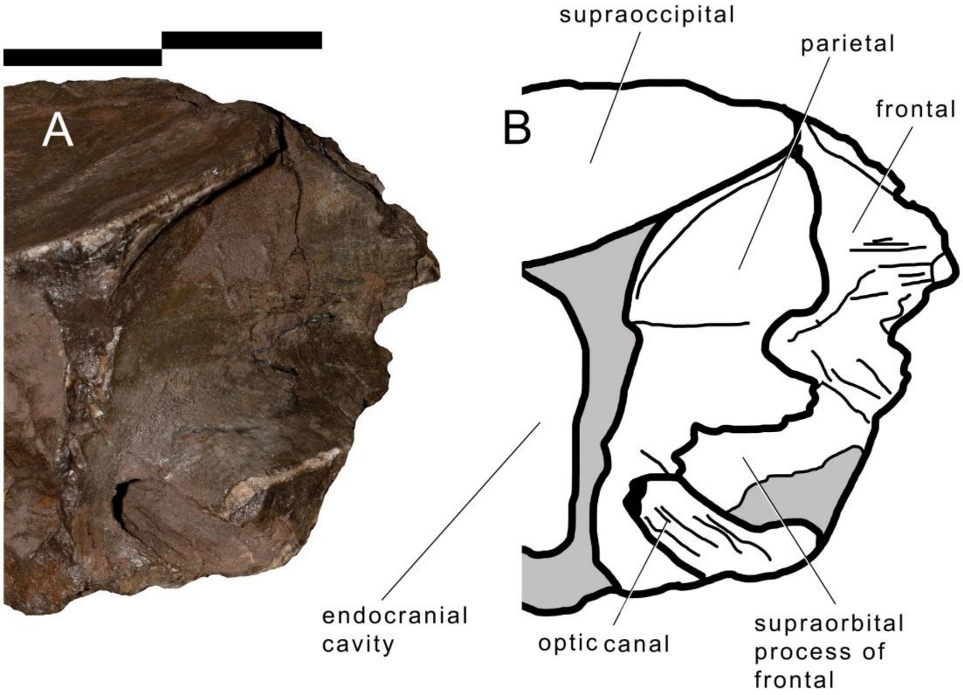

Supplementary Figure S1
Optic canal of *Archaebalaenoptera liesselensis*
Posterolateral view of the optic canal of the holotype skull of Archaebalaenoptera liesselensis *showing the placement of the canal along the posterior border of the supraorbital process of the frontal*. *Damaged areas are in grey. Scale bar equals 10 cm. Photography: Michelangelo Bisconti.*

Supplementary Figure S2

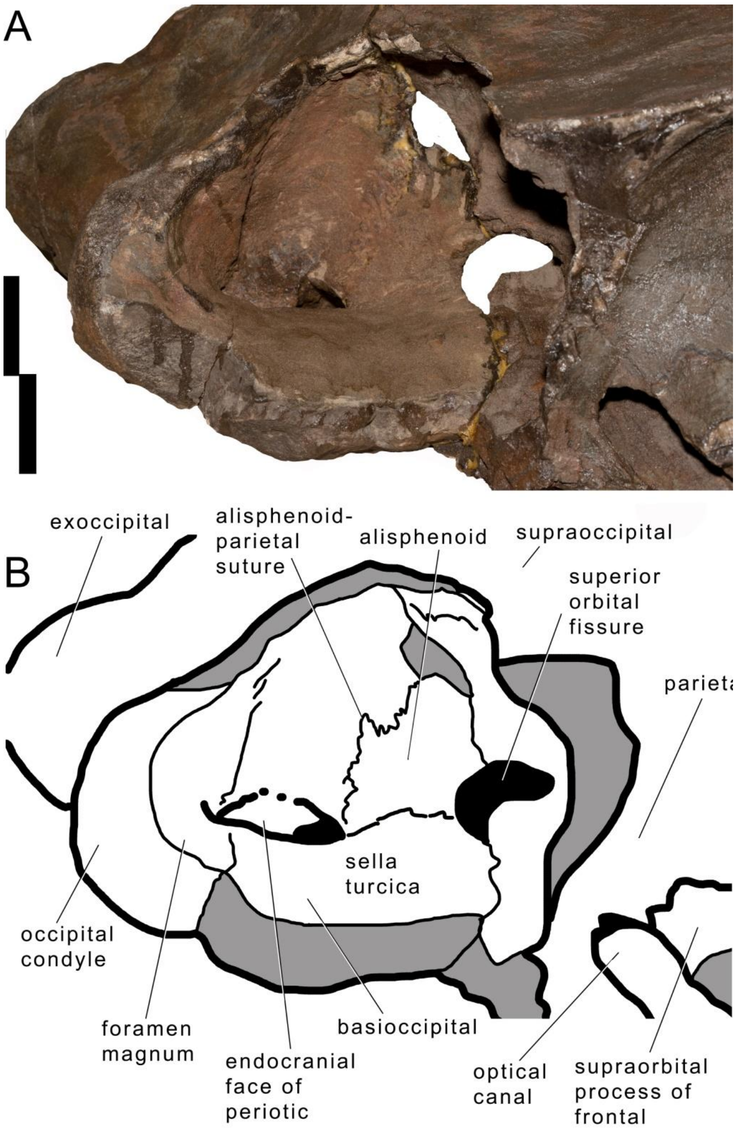

Supplementary Figure S2

Endocranial face of *Archaebalaenoptera liesselensis*

Dorsolateral view of the holotype skull of Archaebalaenoptera liesselensis *showing the internal structures of the neurocranium. (A) photographic representation. (B) interpretation of morphological characters. Damaged areas are in grey. Scale bar equals 10 cm. Photography: Michelangelo Bisconti.*

Supplementary Figure S3

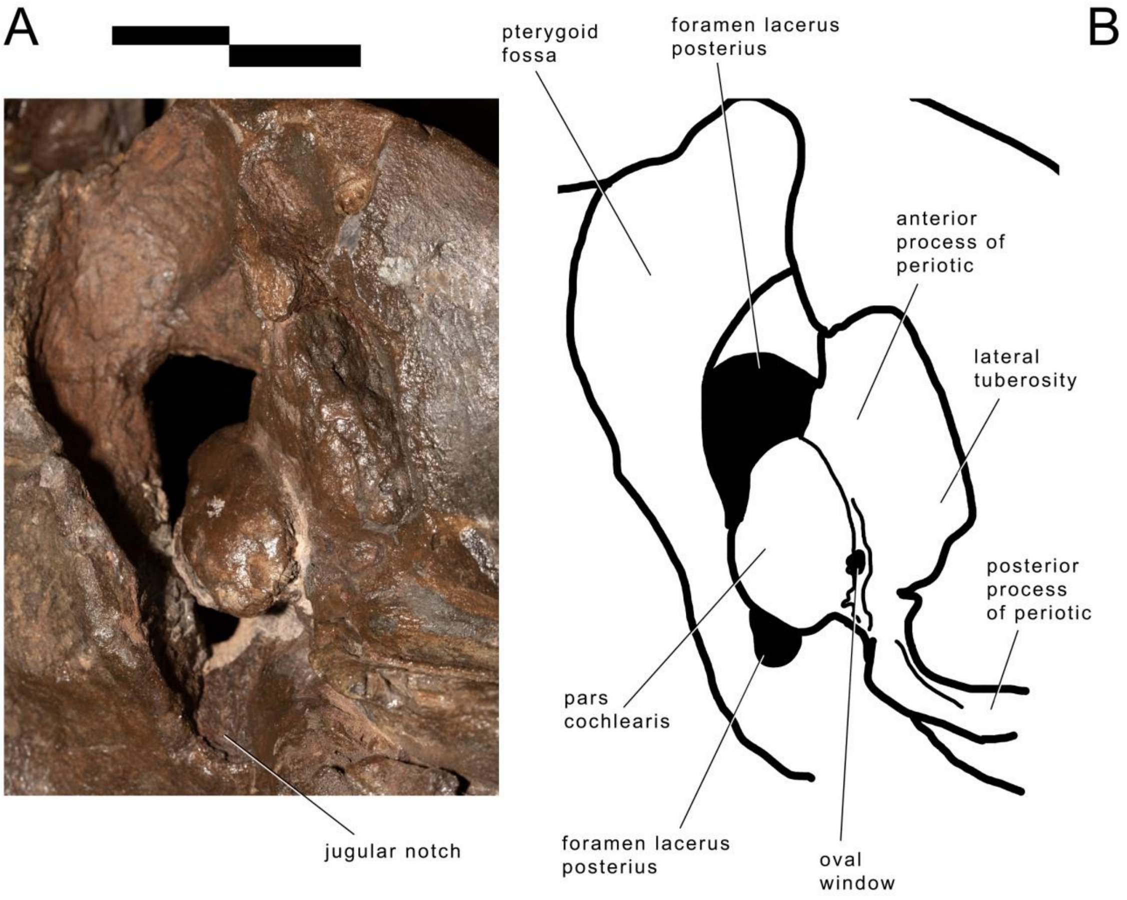

Supplementary Figure S3
Left periotic of *Archaebalaenoptera liesselensis*
Ventrolateral view of the posterolateral portion of the skull of Archaebalaenoptera liesselensis *showing the left periotic in order to represent the tensor tympani groove, the oval window and the ventral protrusion of the pars cochlearis. (A) photographic representation. (B) interpretation of morphological characters. Scale bar equals 10 cm. Photography: Michelangelo Bisconti.*

Supplementary Figure S4

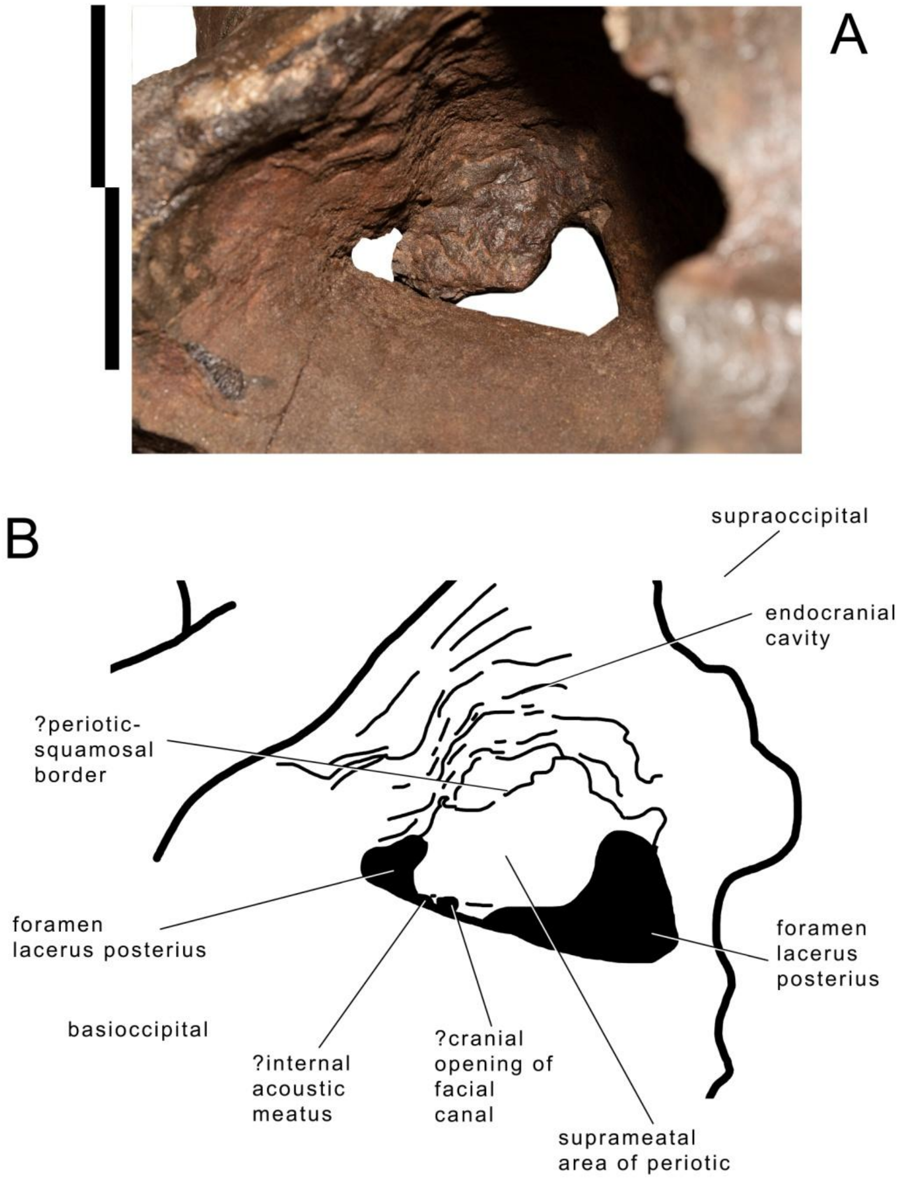

Supplementary Figure S4
Medial side of the periotic of *Archaebalaenoptera liesselensis*
Dorsolateral view of the skull showing the internal face of the neurocranium to represent the medial surface of the periotic of Archaebalaenoptera liesselensis. *(A) photographic representation. (B) interpretation of morphological characters. Scale bar equals 10 cm. Photography: Michelangelo Bisconti.*

Supplementary Figure S5

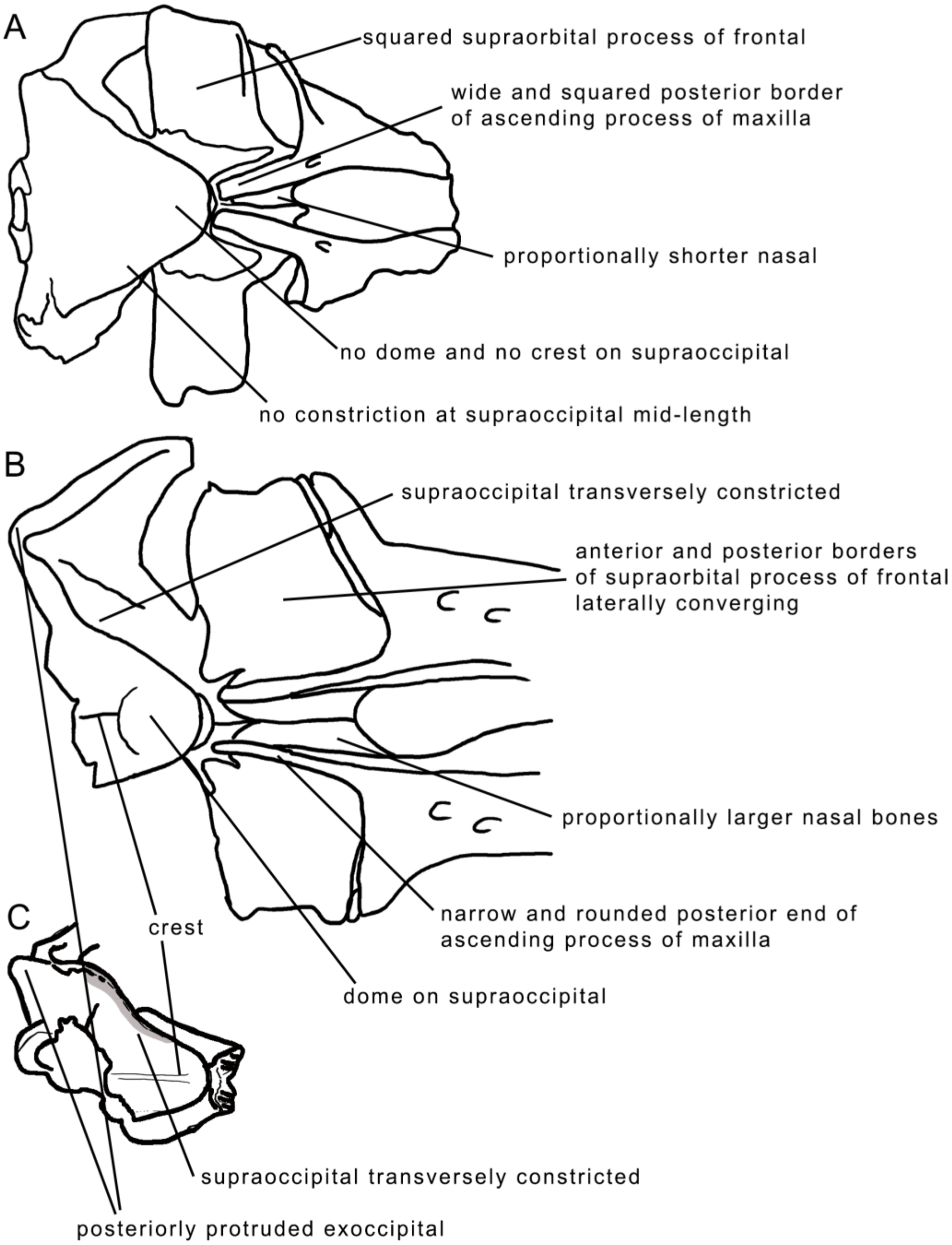

Supplementary Figure S5

Comparative analysis of the *Nehalaennia-Archaebalaenoptera* clade

Comparisons of balaenopterid skulls in dorsal view. Grey area is reconsruction. A, Nehalaennia devossi. *B,* Archaebalaenoptera castriarquati. *C,* Archaebalaenoptera liesselensis. *The skulls are depicted making it constant the supraoccipital length.*

Supplementary Figure S6

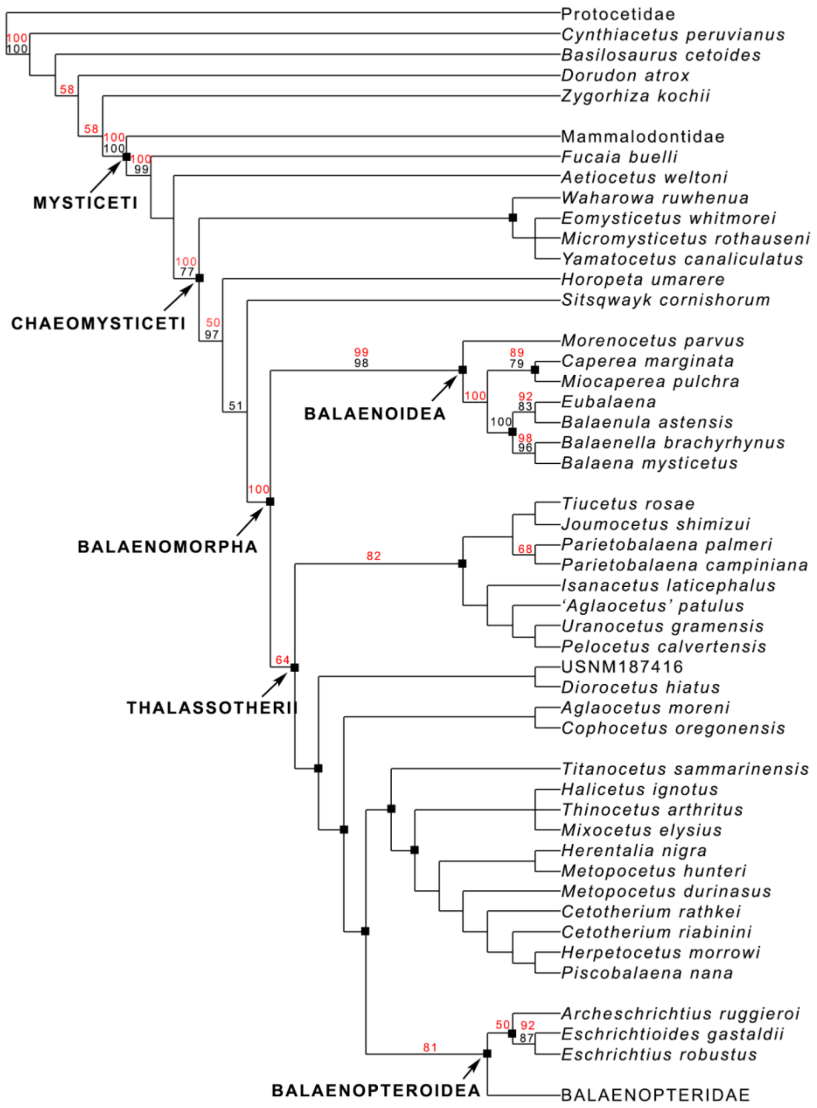

Supplementary Figure S6

Phylogenetic relationships of Mysticeti

Phylogenetic relationships of Mysticeti modified from Bisconti et al. (2019). Black numbers are bootstrap support values, red numbers are symmetric resampling values. See Bisconti et al. (2019) for explanations of methods and discussion of the branching pattern.

Supplementary Figure S7

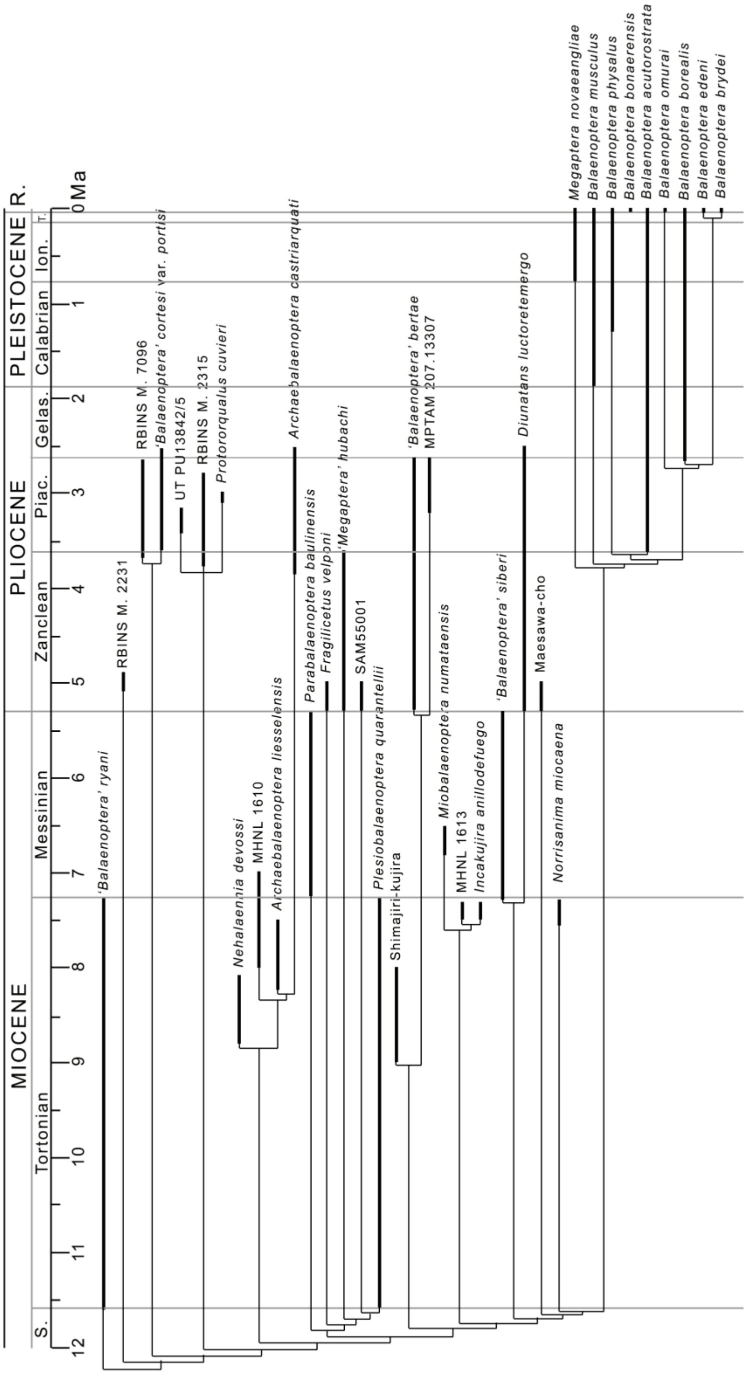

Supplementary Figure S7

Stratigraphic consistency of the balaenopterid phylogeny

Phylogenetic relationships of Balaenopteridae (as from Fig. 16) plotted against a temporal scale to show the agreement between phylogentic position and stratigraphic ages of the OTUs.

Supplementary Figure S8

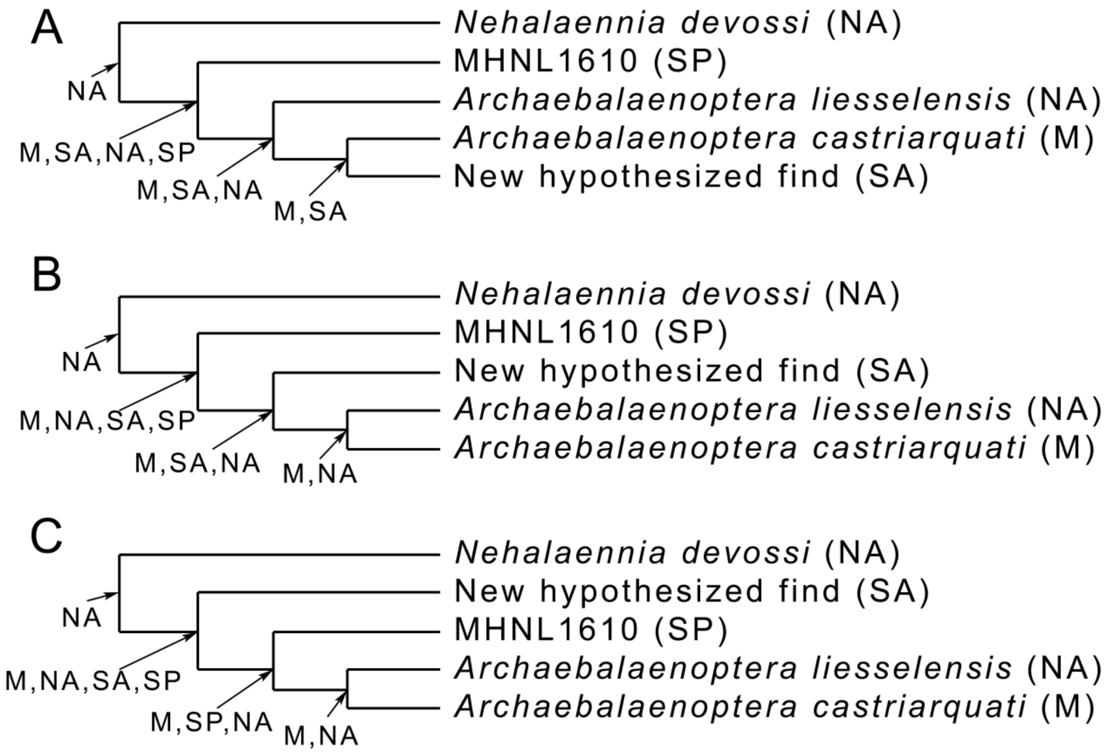

Supplementary Figure S8

Experimental paleobiogeographic analysis
Experimental study of the paleobigeography of Archaebalaenoptera *in the case a new find from South Atlantic is included within the phylogenetic analysis. Method: Fitch's (1985) parsimony (see Methods for explanation). Caption: M, Mediterranean; NA, North Atlantic; SA, South Atlantic; SP, South Pacific.*

Literature cited in the Supplementary Information

Benke H. 1993. Investigations on the osteology and functional morphology of the flipper of whales and dolphins (Cetacea). *Investigations on Cetacea* **24:**9–252.

Bisconti M. 2000. New description, character analysis and preliminary phyletic assessment of two Balaenidae skulls from the Italian Pliocene. *Palaeontographia Italica* **87:**37–66.

Bisconti M. 2005. Morphology and phylogenetic relationships of a new diminutive balaenid from the lower Pliocene of Belgium. *Palaeontology* **48:**793–816.

Bisconti M. 2006. *Titanocetus*, a new baleen whale from the Middle Miocene of northern Italy (Mammalia, Cetacea, Mysticeti). *Journal of Vertebrate Paleontology* **26:**344–354.

Bisconti M. 2007a. A new basal balaenopterid from the Early Pliocene of northern Italy. *Palaeontology* **50:**1103–1122.

Bisconti M. 2007b. Taxonomic revision and phylogenetic relationships of the rorqual-like mysticete from the Pliocene of Mount Pulgnasco, northern Italy (Mammalia, Cetacea, Mysticeti). *Palaeontographia Italica* **91:**85–108.

Bisconti M. 2008. Morphology and phylogenetic relationships of a new eschrichtiid genus (Cetacea: Mysticeti) from the Early Pliocene of northern Italy. *Zoological Journal of the Linnean Society* **153:**161–186.

Bisconti M. 2010. A new balaenopterid whale from the Late Miocene of the Stirone River, northern Italy (Mammalia, Cetacea, Mysticeti). *Journal of Vertebrate Paleontology* **30:**943–958.

Bisconti M. 2011. New description of *'Megaptera' hubachi* Dathe, 1983 based on the holotype skeleton held in the Museum für Naturkunde, Berlin. In: Bisconti M, Roselli A, Borzatti de Loewenstern A, eds. *Climatic Change, Biodiversity, Evolution: Natural History Museum and Scientific Research. Proceedings of the Meeting. Quaderni del Museo di Storia Naturale di Livorno* **23:**37–68.

Bisconti M. 2012. Comparative osteology and phylogenetic relationships of *Miocaperea pulchra*, the first fossil pygmy right whale genus and species (Cetacea, Mysticeti, Neobalaenidae). *Zoological Journal of the Linnean Society* **166:**876–911.

Bisconti M, Bosselaers M. 2016. *Fragilicetus velponi*: a new mysticete genus and species and its implications for the origin of Balaenoperidae (Mammalia, Cetacea, Mysticeti). *Zoological Journal of the Linnean Society* **177:**450–474.

Bisconti M, Lambert O, Bosselaers M. 2013. Taxonomic revision of *Isocetus depawi* (Mammalia, Cetacea, Mysticeti) and the phylogenetic relationships of archaic 'cetothere' mysticetes. *Palaeontology* **56:**95–127.

Bisconti M, Varola A. 2006. The oldest eschrichtiid mysticete and a new morphological diagnosis of Eschrichtiidae. *Rivista Italiana di Paleontologia e Stratigrafia* **119:**447–457.

Boessenecker RW. 2013. A new marine vertebrate assemblage from the Late Neogene Purisima Formation in Central California, part II: Pinnipeds and Cetaceans. *Geodiversitas* **35:**815–940.

Boessenecker RW, Fordyce RE. 2015. Anatomy, feeding ecology, and ontogeny of a transitional baleen whale: a new genus and species of Eomysticetidae (Mammalia: Cetacea) from the Oligocene of New Zealand. *PeerJ* **3**(3):e1129

Bosselaers M, Post K. 2010. A new fossil rorqual (Mammalia, Cetacea, Balaenopteridae) from the Early Pliocene of the North Sea, with a review of the rorqual species described by Owen and Van Beneden. *Geodiversitas* **32:**331–363.

Bouetel V, De Muizon C. 2006. The anatomy and relationships of *Piscobalaena nana* (Cetacea, Mysticeti), a Cetotheriidae s.s. from the early Pliocene of Peru. *Geodiversitas* **28:**319–395.

Buono MR, Fernández MS, Cozzuol MA, Cuitiño JI, and Fitzgerald EMG. 2018. The early Miocene balaenid *Morenocetus parvus* from Patagonia (Argentina) and the evolution of right whales. *PeerJ* **5**:e4148

Caretto PG. 1970. La balenottera delle sabbie plioceniche di Valmontasca (Vigliano d'Asti). *Bollettino della Società Paleontologica Italiana* **9**:3–75.

Clapham PJ. 2002. Humpback whale (*Megaptera novaeangliae*). In: Perrin WF, Wursig B, Thewissen JGM, eds. *Encyclopedia of Marine Mammals*. London: Academic Press, 589–592.

Deméré TA, Berta A, McGowen MR. 2005. The taxonomic and evolutionary history of fossil and modern balaenopteroid mysticetes. *Journal of Mammalian Evolution* **12:**99–143.

Fitzgerald EMG. 2006. A bizarre new toothed mysticete (Cetacea) from Australia and the early evolution of baleen whales. *Proceedings of the Royal Society Series B*, https://doi.org/10.1098/rspb.2006.3664.

Fraas E. 1904. Neue Zeuglodonten aus dem Unteren Mitteleocän vom Mokattam bei Cairo. *Geol. Palaeont. Abh.* **6:**199–220.

Freschi A, Cau S. 2015. La riscoperta di Monte Pulgnasco: nuova collocazione cronostratigrafica e geografica di *Protororqualus cuvieri* (Cetacea: Misticeti, Balaenopteridae). *Quaderni del Museo Civico di Storia Naturale di Ferrara* **3:**21–30.

Gottfried MD, Bohaska DJ, Whitmore FC Jr (1994). Miocene cetaceans of the Chesapeake Group. *Proceedings of the San Diego Society of Natural History* **29:**229–238.

Govender R. 2019. Fossil cetaceans from Duinefontein (Koeberg) an early Pliocene site on the southwestern Cape, South Africa. *Palaeontologia Electronica* **22:**1.6A.

Govender R, Bisconti M, Chinsamy A. 2016. A late Miocene–early Pliocene baleen whale assemblage from Langebaanweg, west coast of South Africa (Mammalia, Cetacea, Mysticeti). *Alcheringa* **40:**542–555.

Horwood J. 2002. Sei whale (*Balaenoptera borealis*). In: Perrin WF, Wursig B, Thewissen JGM, eds. *Encyclopedia of Marine Mammals*. London: Academic Press, 1069–1071.

Hulbert RC Jr. 1998. Postcranial osteology of the North American Middle Eocene protocetid *Georgiacetus*. In: Thewissen JGM, ed. *The emergence of whales*. New York: Plenum Press, 235–267.

Hulbert RC Jr, Petkewich RM, Bishop GA, Bukry D, Aleshire DP. 1996. A new Middle Eocene protocetid whale (Mammalia: Cetacea: Archaeoceti) and associated biota from Georgia. *Journal of Paleontology* **72:**907–927.

Jones ML, Schwartz SL. 2002. Gray whale (*Eschrichtius robustus*). In: Perrin WF, Wursig B, Thewissen JGM, eds. *Encyclopedia of Marine Mammals*. London: Academic Press, 524–537.

Kato H. 2002. Bryde’s whales (*Balaenoptera edeni* and *B. brydei*). In: Perrin WF, Wursig B, Thewissen JGM, eds. *Encyclopedia of Marine Mammals*. London: Academic Press, 171–177.

Kellogg R. 1936. A review of the Archaeoceti. *Carnegie Institution Washington* **482:**1–366.

Kellogg R. 1934a. The Patagonia fossil whalebone whale, *Cetotherium moreni* (Lydekker). *Contributions in Palaeontology, Carnegie Insitution Washington* **447:**63–81.

Kellogg R. 1934b. A new cetothere from the Modelo Formation at Los Angeles, California. *Contributions in Palaeontology, Carnegie Insitution Washington* **447:**85–104.

Kellogg R. 1965. A new whalebone whale from the Miocene Calvert Formation. *United States National Museum Bulletin* **247:**1–45.

Kellogg R. 1968a. Miocene Calvert mysticetes described by Cope. *United States National Museum Bulletin* **247:**103–132.

Kellogg R. 1968b. A hitherto unrecognized Calvert mysticete. *United States National Museum Bulletin* **247:**133–161.

Kellogg R. 1968c. A sharp-nosed cetothere from the Miocene Calvert. *United States National Museum Bulletin* **247:**163–197.

Kellogg R. 1968d. Supplement to description of Parietobalaena palmeri. *United States National Museum Bulletin* **247:**175–197.

Kemper CM. 2002. Pygmy right whale (*Caperea marginata*). In: Perrin WF, Wursig B, Thewissen JGM, eds. *Encyclopedia of Marine Mammals*. London: Academic Press, 1010–1013.

Kenney RD. 2002. North Atlantic, North Pacific, and Southern right whales (*Eubalaena glacialis, E. japonica,* and *E. australis*). In: Perrin WF, Wursig B, Thewissen JGM, eds. *Encyclopedia of Marine Mammals*. London: Academic Press, 806–813.

Kimura T, Hasegawa Y. 2010. A new baleen whale (Mysticeti: Cetotheriidae) from the earliest Late Miocene of Japan and a reconsideration of the phylogeny of cetotheres. *Journal of Vertebrate Paleontology* **30:**577–591.

Kimura T, Ozawa T. 2002. A new cetothere (Cetacea: Mysticeti) from the Early Miocene of Japan. *Journal of Vertebrate Paleontology* **22:**684–702.

Kimura T, Adaniya A, Oishi M, Marx FG, Hasegawa Y. 2015. A Late Miocene balaenopterid ("Shimajirikujira") from the Okamishima Formation, Shimajiri Group, Miyako Island, Okinawa, Japan. *Bulletin of the Gunma Museum of Natural History* **19:**39–48. (in Japanese with English abstract).

Leslie MS, Peredo CM, Pyenson ND. 2019. *Norrisanima miocaena*, a new generic name and redescription of a stem balaenopteroid mysticete (Mammalia, Cetacea) from the Miocene of California. *PeerJ* **7**:e7629 http://doi.org/10.7717/peerj.7629.

Marx FG, Bosselaers MEJ, Louwye S. 2015. A new species of *Metopocetus* (Cetacea, Mysticeti, Cetotheriidae) from the late Miocene of Netherlands. *PeerJ* **4**:e1572; DOI 10.7717/peerj.1572

Marx FG, Kohno N. 2016. A new Miocene baleen whale from the Peruvian desert. *Royal Society Open Science* **3:**160542. http://dx.doi.org/10.1098/rsos.160542.

Oishi M, Kawakami T, Hasegawa, Y. 1985. Pliocene baleen whales and bony-toothed bird from Iwate Prefecture, Japan (Parts I–VI). *Bulletin of the Iwate Prefectural Museum* **3:**143–157.

Packard EL, Kellogg R. 1934. A new cetothere from the Miocene Astoria Formation of Newport, Oregon. *Publication of Carnegie Institution Washington* **447**:1–62.

Peredo CM, Uhen MD. 2016. A new basal Chaeomysticete (Mammalia: Cetacea) from the Oligocene Pysht Formation of Washington, USA. *Papers in Palaeontology* **2016**:1–22.

Perrin WR, Brownell RL Jr. 2002. Minke whales (*Balaenoptera acutorostrata* and *B. bonaerensis*). In: Perrin WF, Wursig B, Thewissen JGM, eds. *Encyclopedia of Marine Mammals*. London: Academic Press, 750–754.

Pilleri G. 1986. *Beobachtungen an den fossilen Cetaceen des Kaukasus*. Ostermundigen: Brain Anatomy Institute.

Pilleri G. 1989. *Balaenoptera siberi*, ein neuer Spätmiozäner Bartenwal aus der Pisco-Formation Perus. In: Pilleri G, ed. Beiträge zur Paläontologie der Cetaceen Perus. Ostermundigen: Hirnanatomisches Institut der Universitat Bern (Schweiz), 63–84.

Rugh DJ, Shelden KEW. 2002. Bowhead whale (*Balaena mysticetus*). In: Perrin WF, Wursig B, Thewissen JGM, eds. *Encyclopedia of Marine Mammals*. London, Academic Press, 12–131.

Sanders AE, Barnes LG. 2002a. Paleontology of the Late Oligocene Ashley and Chandler Bridge Formations of South Carolina, 2: *Mycromysticetus rothauseni*, a primitive cetotheriid mysticete (Mammalia: Cetacea). In: Emry RJ, ed. *Cenozoic mammals of land and sea: tributes to the career of Clayton E. Ray*. *Smithsonian Contributions in Paleobiology* **97:**271–293.

Sanders AE, Barnes LG. 2002b. Paleontology of the late Oligocene Ashley and Chandler Bridge formations of South Carolina, 3: Eomysticetidae, a new family of primitive mysticetes (Mammalia: Cetacea). In: Emry RJ, ed. *Cenozoic mammals of land and sea: tributes to the career of Clayton E. Ray*. *Smithsonian Contribution in Paleobiology* **93:**313–356.

Sears R. 2002. Blue whale (*Balaenoptera musculus*). In Perrin WF, Wursig B, Thewissen JGM, eds. *Encyclopedia of Marine Mammals.* London: Academic Press, 112–116.

Sears R, Calambokidis J. 2002. Assessment and update report Status Report on the Blue Whale *Balaenoptera musculus* Atlantic population Pacific population in Canada. Ottawa: COSEWIC, 1–32.

Steeman ME. 2009. A new baleen whale from the Late Miocene of Denmark and early mysticete hearing. *Palaeontology* **52:**1169–1190.

Tanaka Y, Watanabe M. 2019. An early and new member of Balaenopteridae from the upper Miocene of Hokkaido, Japan. *Journal of Systematic Palaeontology* **17:**1197–1211.

Tsai C-H, Boessenecker RW. 2017. The earliest-known fin whale, *Balaenoptera physalus*, from the Early Pleistocene of Northern California, U.S.A. *Journal of Vertebrate Paleontology* e1306536.

Tsai C-H, Fordyce RE. 2015. The Earliest Gulp-Feeding Mysticete (Cetacea: Mysticeti) from the Oligocene of New Zealand. *Journal of Mammalian Evolution* **23:** 33–59.

Uhen MD. 1998. Middle to Late Eocene basilosaurines and dorudontines. In: Thewissen JGM, ed. *The emergence of whales: evolutionary patterns in in the origin of Cetacea*. New York: Plenum Press, 29–63.

Uhen MD. 2004. Form, function, and anatomy of *Dorudon atrox* (Mammalia, Cetacea): an archaeocete from the middle to late Eocene of Egypt. *University Michigan Paper in Paleontology* **34:**1–222.

Wada S, Oishi M, Yamada TK. 2007. A newly discovered species of living baleen whale. *Nature* **426:** 278–291.

Zeigler CV, Chan GL, Barnes LG. 1997. A new late Miocene balaenopterid whale (Cetacea: Mysticeti), Parabalaenoptera baulinensis, (new genus and species) from the Santa Cruz Mudstone, Point Reyes Peninsula, California. *Proceedings of the California Academy of Sciences* **50**:115–138.
